# New piperazine derivatives helvamides B–C from the marine-derived fungus *Penicillium velutinum* ZK-14 uncovered by OSMAC (One Strain Many Compounds) strategy

**DOI:** 10.1007/s13659-024-00449-9

**Published:** 2024-05-21

**Authors:** Gleb V. Borkunov, Elena V. Leshchenko, Dmitrii V. Berdyshev, Roman S. Popov, Ekaterina A. Chingizova, Nadezhda P. Shlyk, Andrey V. Gerasimenko, Natalya N. Kirichuk, Yuliya V. Khudyakova, Viktoria E. Chausova, Alexandr S. Antonov, Anatoly I. Kalinovsky, Artur R. Chingizov, Ekaterina A. Yurchenko, Marina P. Isaeva, Anton N. Yurchenko

**Affiliations:** 1 G.B. Elyakov Pacific Institute of Bioorganic Chemistry, Far Eastern Branch of the Russian Academy of Sciences, 159 Prospect 100-Letiya Vladivostoka, Vladivostok, 690022 Russian Federation; 2 Far Eastern Federal University, Vladivostok, 690922 Russian Federation; 3 Institute of Chemistry, Far Eastern Branch of the Russian Academy of Sciences, 159 Prospect 100-Letiya Vladivostoka, Vladivostok, 690022 Russian Federation

**Keywords:** *Penicillium velutinum*, OSMAC strategy, Metal ions stress, LC/MS, Metabolite profile, Bioassay

## Abstract

Four extracts of the marine-derived fungus *Penicillium velutinum* J.F.H. Beyma were obtained via metal ions stress conditions based on the OSMAC (One Strain Many Compounds) strategy. Using a combination of modern approaches such as LC/UV, LC/MS and bioactivity data analysis, as well as in silico calculations, influence metal stress factors to change metabolite profiles *Penicillium velutinum* were analyzed. From the ethyl acetate extract of the *P. velutinum* were isolated two new piperazine derivatives helvamides B (**1**) and C (**2**) together with known saroclazin A (**3**) (4*S*,5*R*,7*S*)-4,11-dihydroxy-guaia-1(2),9(10)-dien (**4**). Their structures were established based on spectroscopic methods. The absolute configuration of helvamide B (**1**) as 2*R*,5*R* was determined by a combination of the X-ray analysis and by time-dependent density functional theory (TD-DFT) calculations of electronic circular dichroism (ECD) spectra. The cytotoxic activity of the isolated compounds against human prostate cancer PC-3 and human embryonic kidney HEK-293 cells and growth inhibition activity against yeast-like fungi *Candida albicans* were assayed.

**Graphical Abstract:**

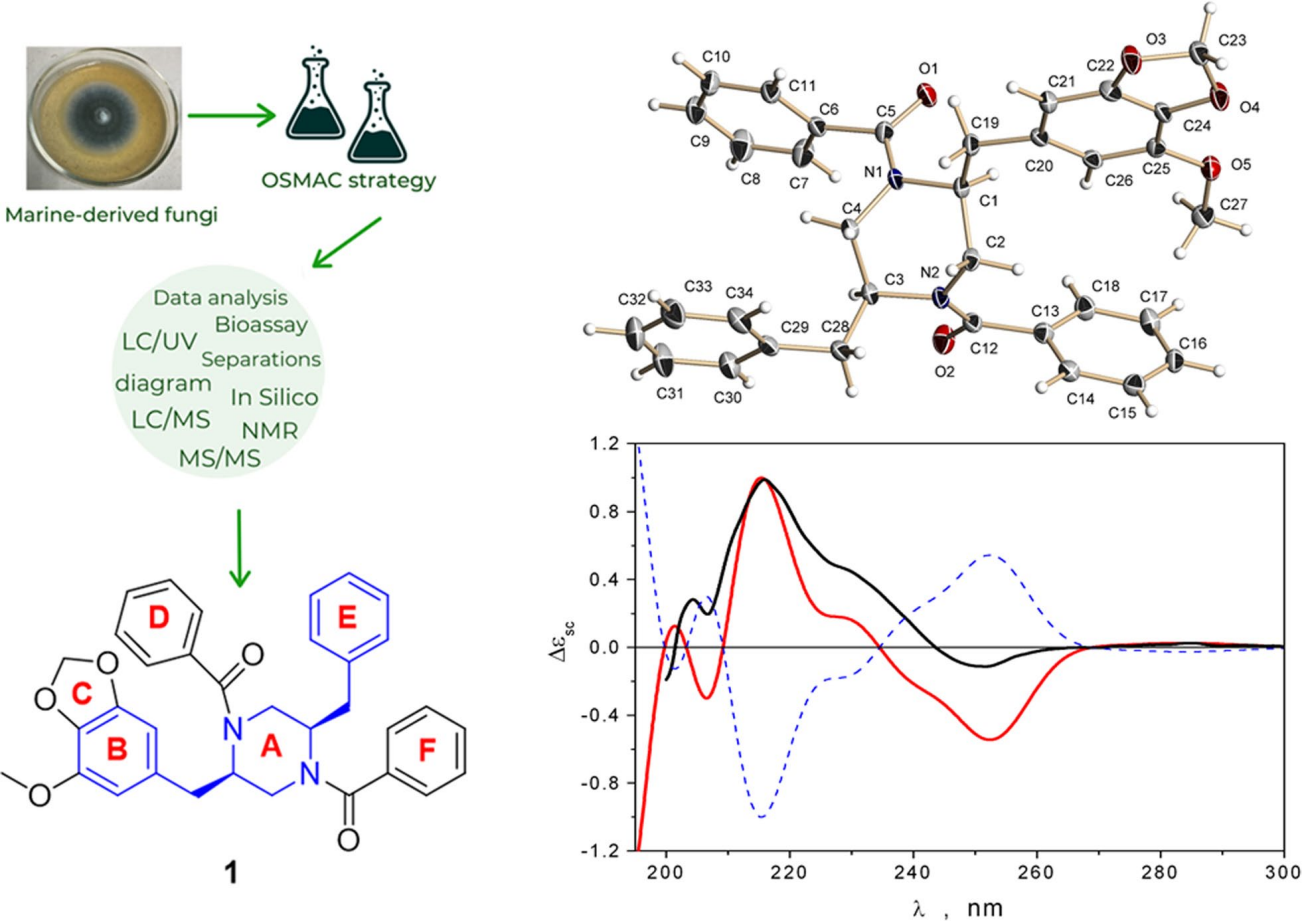

**Supplementary Information:**

The online version contains supplementary material available at 10.1007/s13659-024-00449-9.

## Introduction

Secondary metabolites from marine-derived fungi have a wide range of biological activities due to their structural diversity, and have been proven to be a major source of drug-lead compounds [1, 2]. However, the metabolic pathways of microorganisms during cultivation under conditions close to natural are severely limited, and as a result, many metabolites cannot be formed [3]. The purposeful variation of culture conditions, also known as the OSMAC strategy, is an approach to metabolic pathway activation that has been successfully applied to the discovery of new lead compounds in fungi [3].

Recent advances in untargeted metabolomics, including analytical techniques and data analysis methods, have facilitated the use of mass spectrometry (MS)-based approaches to rapidly estimate microbial chemodiversity and identify specific metabolites overexpressed under varying conditions [4]. LC/MS provides high sensitivity and accuracy, allowing the detection and analysis of metabolites present at low concentrations in complex extracts [5]. The qualitative and quantitative information obtained from LC/MS analysis can be used to compare the metabolomes of distinct microbial extracts grown under different conditions, allowing the assessment of how such conditions affect the metabolite profile [5]. Although the identification of the metabolites detected can be challenging, modern data analysis techniques have enabled the identification of novel chemical scaffolds and analogs of known substances in complex extracts, making MS-based approaches an essential tool for screening large numbers of microorganisms to identify potential sources of novel bioactive metabolites [6].

The fungi of *Penicillium* genus are among the most widespread terrestrial and marine fungal organisms on the planet [7]. *Penicillium* species produce a wide range of secondary metabolites, including terpenes [8–11], polyketides [12, 13], meroterpenoids [8, 14, 15], alkaloids [16], and peptides [17, 18] with various biological activities. Historically, it was from the fungus of the genus *Penicillium* that the first antibiotic was isolated, and the tendency to isolate a variety of low-molecular compounds from fungi of this genus, both terrestrial and marine, has persisted. As of 2014, more than 390 novel natural products have been obtained from marine-derived *Penicillium* fungi [19] isolated from substrates such as sediments, algae and seagrasses, vertebrates and invertebrates. Moreover, 72 compounds from marine-derived *Penicillium* fungi and their antimicrobial activities were reported from 2020 to 2023 [20]. Our investigation of *Penicillium* fungi from marine algae and seagrasses has led to the discovery of new strains that produce antibacterial and anticancer compounds [13, 21–25]. These investigations were continued with the isolation of the *Penicillium velutinum* strain from the rhizome seagrass *Zostera marina*.

*Penicillium velutinum* belonging to the subgenus *Aspergilloides*, section *Exilicaulis*, series *Lapidosa*, that includes species: *Penicillium aotearoae*, *P. atrosanguineum*, *P. burgense*, *P. diabolicalicense*, *P. hemitrachum*, *P. lapidosum*, *P. maclennaniae*, *P. melinii*, *P. namyslowskii*, *P. raciborskii*, *P. smithii*, *P. terrenum*, *P. velutinum* and *P. xanthomelinii*. Earlier from the *Penicillium velutinum* were isolated fulvinol, citromycetin [26], and spirocitromycetin [27] skeletally unprecedented anti-osteoporotic agents that may be of significance for drug discovery. Well-known biologically active polyketides griseofulvin, mevastatin, and their derivatives have been isolated from the *P. namyslowskii* strain [28]. Thus, according to the literature data, the fungi belonging to this section are poorly studied from a chemical point of view, at the same time, they can be producers of unique biologically active metabolites.

Thus, in accordance with the OSMAC strategy, the first step of the present work was to study the metabolite profile of the *Penicillium velutinum* ZK-14 strain and the effect of different inorganic salts on this strain using LC/UV and LC/MS data analysis. The second step was the isolation of individual compounds from the ethyl acetate extract of *P. velutinum* cultured on the medium, which led to the highest yield of various compounds. Our investigation led to discovery new piperazine derivatives helvamides B (**1**) and C (**2**) (Fig. 9). Moreover, the cytotoxicity against human prostate cancer PC-3 and normal human embryonic kidney HEK 293T cells as well as antimicrobial activity against yeast-like fungus *Candida albicans* and DPPH radical scavenging properties of all obtained extracts and individual compounds were assayed.

## Results and discussion

### LC/MS chromatogram of the *Penicillium velutinum* ZK-14

**Fig. 1:**
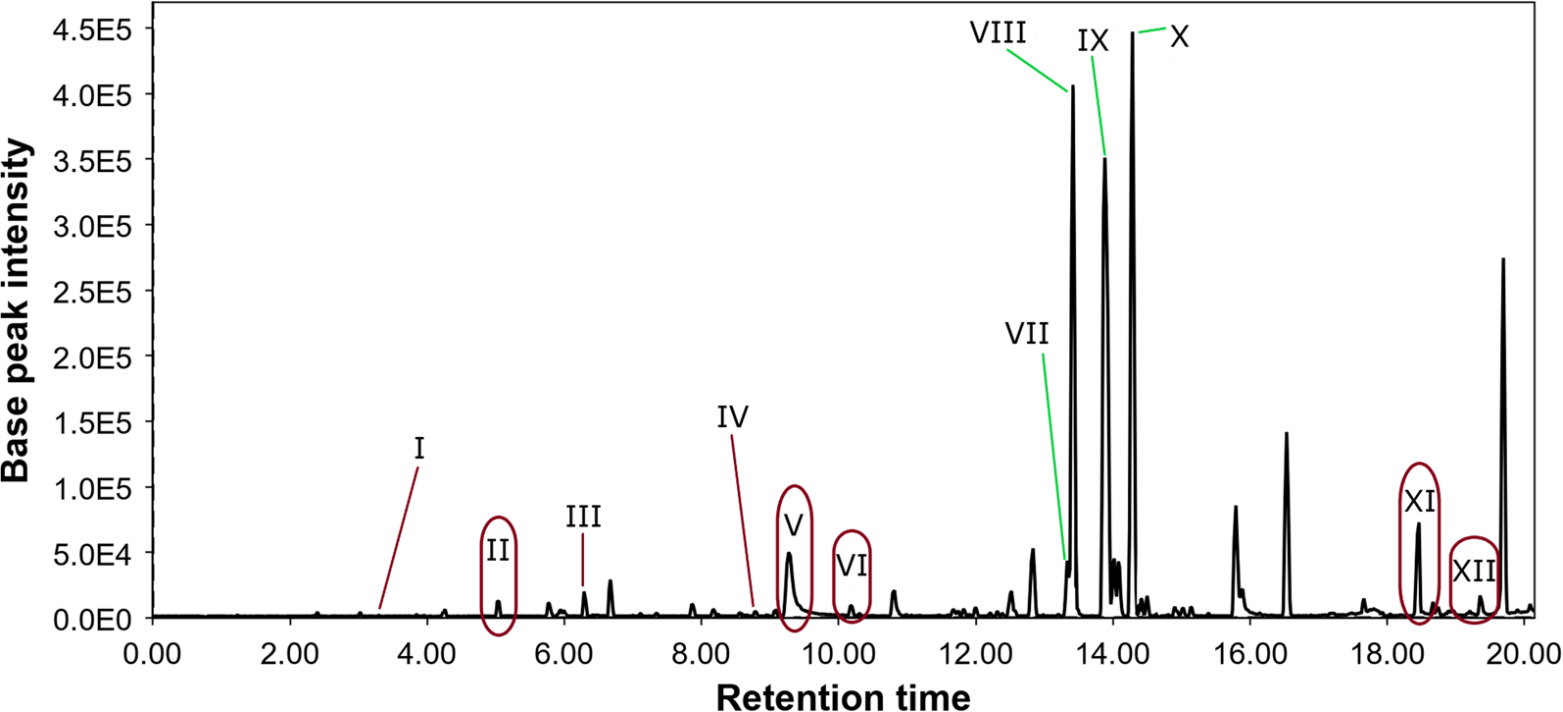
LC/MS chromatogram of the extract of *P. velutinum* ZK-14 (Pv0)

The purified ethyl acetate extract of the fungus *P. velutinum* ZK-14 cultivated on the rice media (Pv0) was investigated by LC/MS, and the LC/MS chromatogram was analyzed using the Global Natural Product Social Molecular Networking (GNPS) database. Totally 12 peaks were annotated at the LC/MS chromatogram of *P. velutinum* ZK-14 (Fig. 1).

The peak **V** detected at 9.4 min with *m/z* 291.0480 ([M-H_2_O + H]^+^) corresponded to the molecular formula C_14_H_12_O_8_. It was identified as a widespread fungal metabolite, fulvic acid, through the comparison of experimental MS/MS spectra with the GNPS database (MQScore 0.87). This is a well-known fungal metabolite that was first isolated from the fungi *P. griseofulvum*, *P. flexuosum,* and *P. brefeldianum* in 1935 [29, 30]. For the other 11 peaks, only the molecular formulas corresponding to the exact masses were suggested (Appendix, Table 5).

**Table 1:** The biological activities of *P. velutinum* ZK-14 extracts

Extract code	Cell viability, % of control		Inhibition of *C. albicans* growth, %	DPPH radical scavenging activity	
	PC-3	HEK-293		% of control	IC_50_, µM
Pv0	52.7 ± 0.8	34.9 ± 3.2	42.8 ± 2.8	40.8 ± 2.3	34.9 ± 3.2
PvMg	49.8 ± 3.0	40.0 ± 0.6	45.3 ± 1.3	74.7 ± 1.4	40.0 ± 0.6
PvFe	80.4 ± 4.2	82.1 ± 1.1	41.5 ± 0.6	96.9 ± 2.1	82.1 ± 1.1
PvZn	49.9 ± 2.1	54.8 ± 0.6	26.9 ± 2.7	41.9 ± 1.2	54.8 ± 0.6
PvNi	54.0 ± 3.5	49.1 ± 0.7	37.5 ± 0.2	42.9 ± 2.9	49.1 ± 0.7
Quercetin				98.7 ± 1.9	41.8 ± 2.9

The concentrations of extracts were 100 μg/mL. All data are presented as a mean ± standard error of the mean (SEM)

### The effect of culture conditions on the metabolic profile of extracts of *P. velutinum* ZK-14

#### LC/UV

**Fig. 2:**
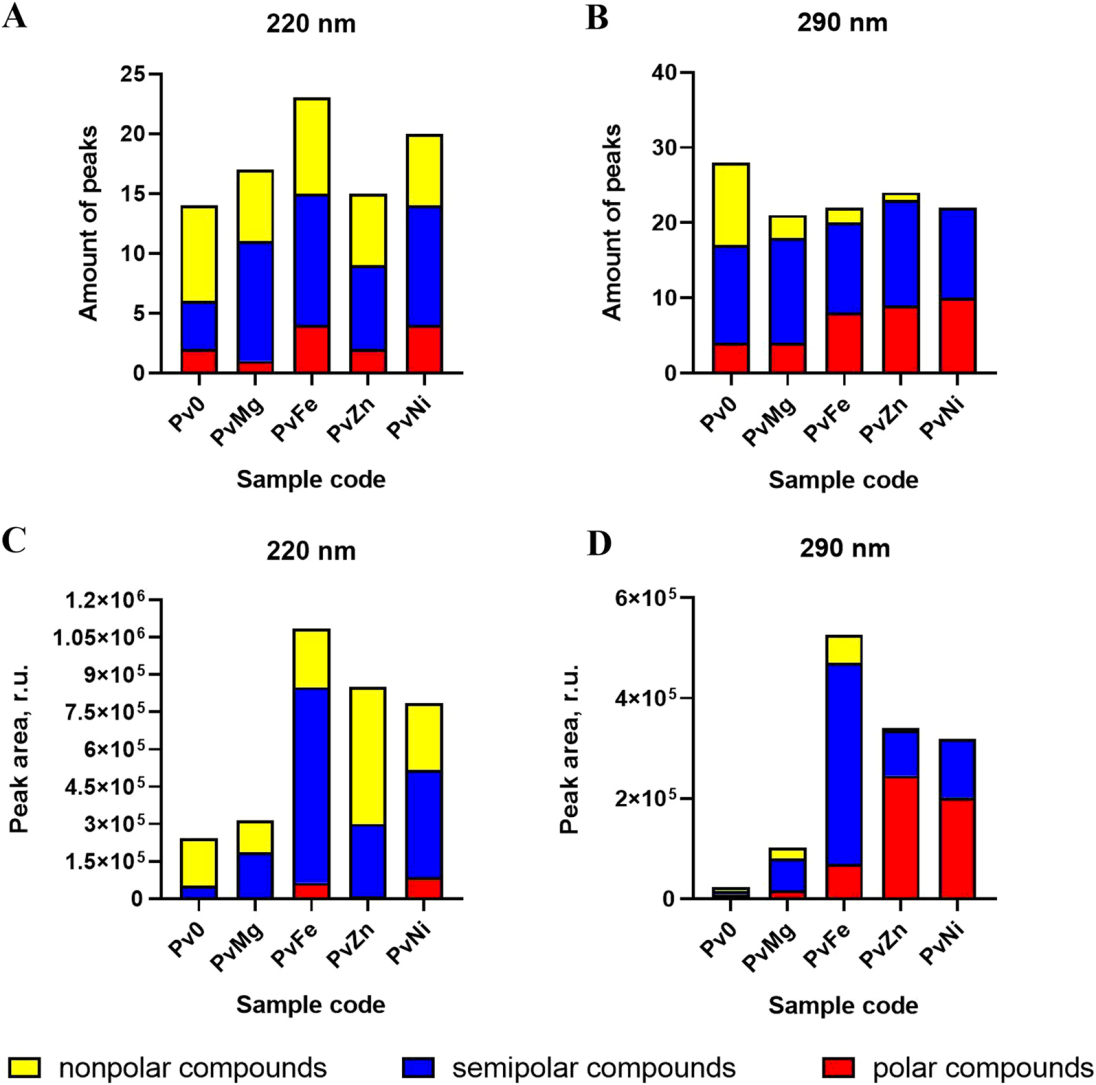
**A** The total number of peaks in LC/UV chromatograms detected at 220 nm; **B** the total number of peaks in LC/UV chromatograms detected at 290 nm; **C** the total area of the peaks detected at 220 nm; **D** the total area of the peaks detected at 290 nm

For purified ethyl acetate extracts PvMg (MgCl_2_·6H_2_O), PvFe (Fe(NO_3_)_3_·9H_2_O), PvZn (ZnCl_2_), and PvNi (Ni(NO_3_)_2_·6H_2_O) LC/UV chromatograms at 220 (Additional file 1: Figs. S31–S35) and 290 nm (Additional file 1: Fig. S30) were obtained. Each chromatogram, based on retention times, was divided into three zones: high polarity (9–21 min, 15–35% MeCN), middle polarity (21–39 min, 35–65% MeCN), and low polarity (> 39 min, 65–100% MeCN). The total number of peaks and total area of the peaks in each zone were calculated and presented in Fig. 2.

The LC/UV chromatogram data of the PvMg extract showed a slight increase in the number of peaks in this extract, comparable to the number of peaks of other extracts; however, the total peak area remained at the same lee control extract. A decrease in highly polar compounds in PvMg compared to the control extract should also be noted.

The addition of ZnCl_2_ and Ni(NO_3_)_2_ led to a slight increase in the number of detected peaks at 290 nm in the middle polarity zone in comparison with the control; however, the peaks in the high polarity zone detected at 290 nm increased markedly.

The greatest changes were observed in the extract obtained by cultivating the fungus with Fe(NO_3_)_3_. The LC/UV chromatogram data of the PvFe extract showed increased peak areas at 220 and 290 nm, corresponding to low, middle, and high polarity metabolites, respectively, compared to the control extract and other extracts. The number of peaks in the middle polarity zone was comparable to that of the other extracts, with the exception of the control.

Thus, the LC/UV chromatogram data showed that the addition of metal salts during cultivation affected the total number of peaks and the total area below the peaks compared to the control extract.

#### LC/MS analysis

LC/MS chromatograms were obtained for a detailed analysis of the changes in the metabolite profiles of *P. velutinum* ZK-14 cultivated based on the OSMAC strategy for all purified ethyl acetate extracts. Metabolite profiling of the analyzed extracts using LC/MS allowed the detection of numerous compounds. In total, 16 peaks were detected, all identified peaks and their corresponding compounds are shown in Table 5.

**Fig. 3:**
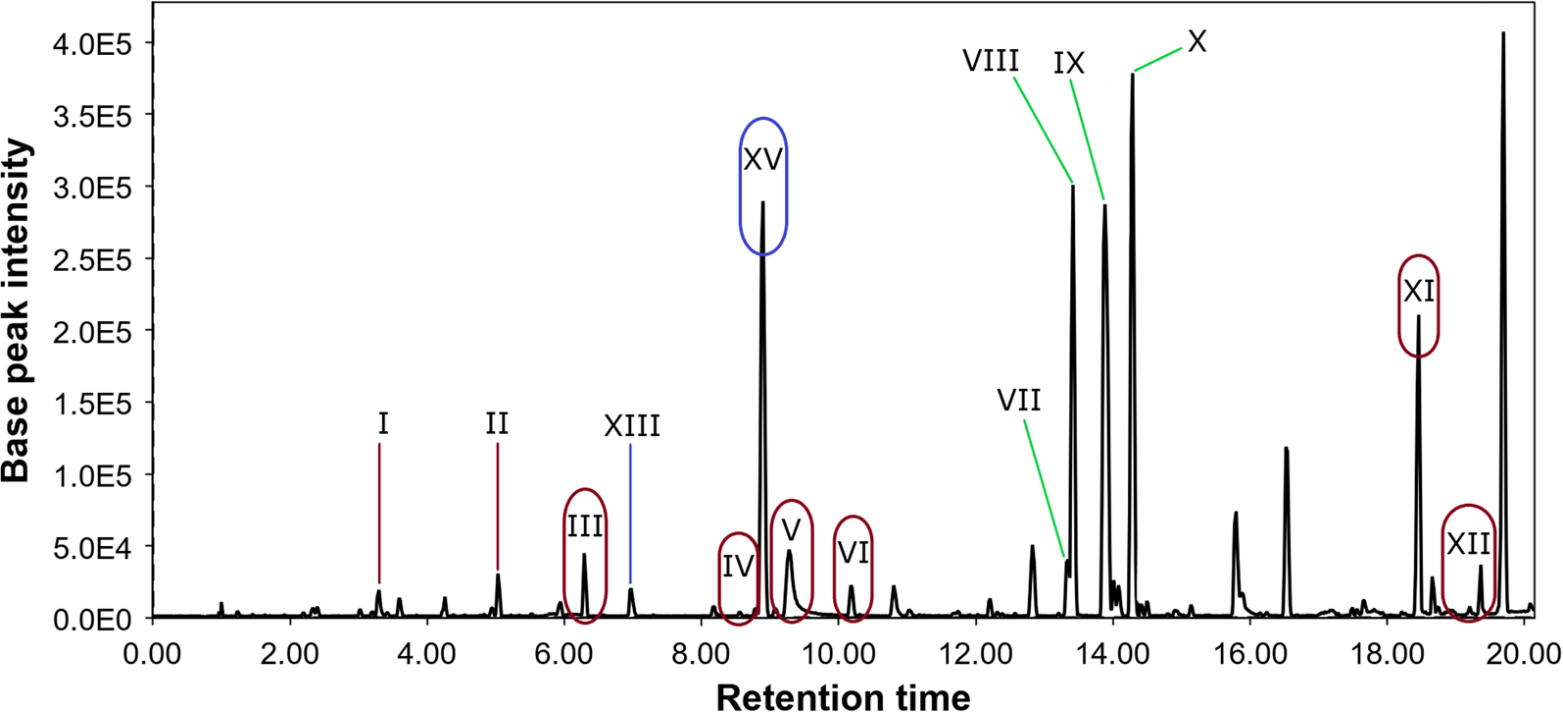
LC/MS chromatogram of the PvMg extract

The PvMg chromatogram (Fig. 3) differed from the control chromatogram in the presence of additional peaks **XIII** and **XV**. The peak **XIII** detected at 7.0 min with *m/z* 301.0699 ([M + H]^+^) corresponded to the molecular formula C_16_H_12_O_6_, which the same as the known anthraquinone polyketide fallacinol [31] isolated from the fermentation extract of *Talaromyces stipitatus* KUFA 0207 [32] and dried roots of *Polygonum cuspidatum* [33]. This was confirmed by comparing the experimental MS/MS spectra with the GNPS database (MQScore 0.92). The peak **XV** at 8.9 min contains two compounds with *m/z* 209.0437 ([M-C_8_H_10_O_2_ + H]^+^) and *m/z* 333.0967 ([M + H]^+^), which correspond to molecular formulas such as C_18_H_18_O_7_ and C_17_H_16_O_7_, which were the same as the widespread fungal metabolites methylsulochrin [34, 35] and sulochrin [35–38]. This was proven through a comparison of experimental MS/MS spectra with the GNPS database (MQScore 0.83 and 0.95, respectively).

**Fig. 4:**
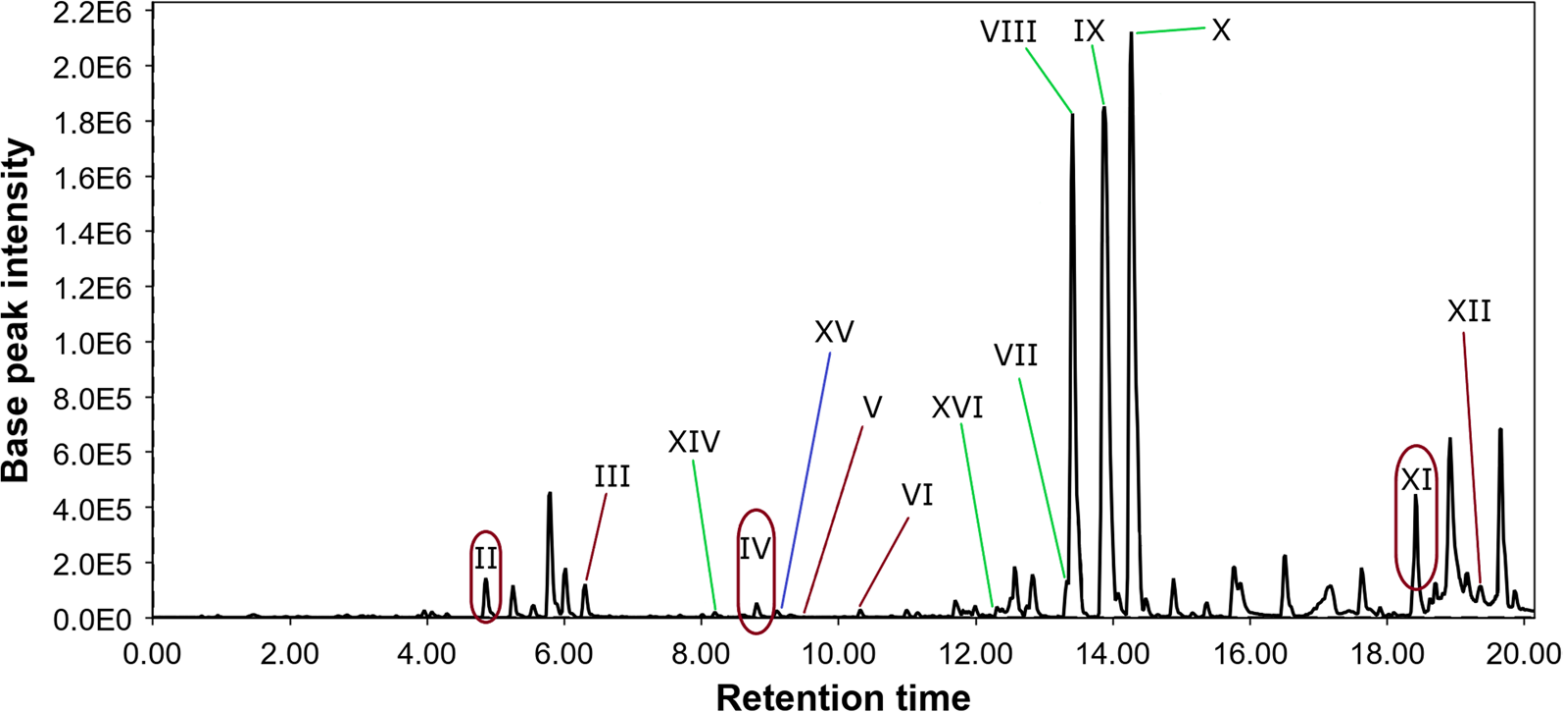
LC/MS chromatogram of the PvFe extract

The PvFe chromatogram (Fig. 4) differs from that of the control by the presence of additional peaks **XIV, XV,** and **XVI**, but by the absence of peaks **I** and **XIII**. It is also worth noting that the intensity of all peaks in the chromatogram increased by approximately five times. The peak **XV** of the PvFe extract contained only methylsulochrine, unlike the PvMg extract, and the intensity of this peak was extremely low.

**Fig. 5:**
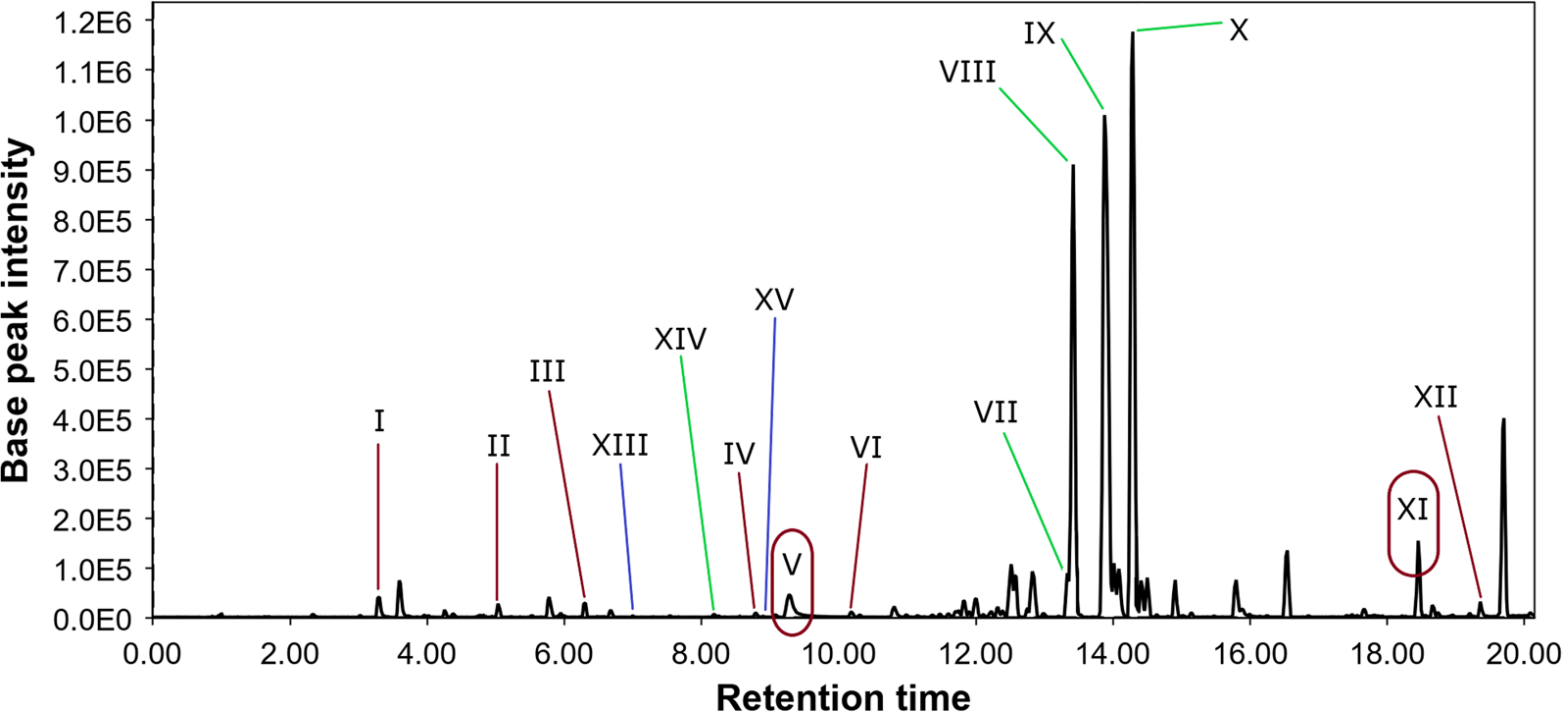
LC/MS chromatogram of the PvZn extract

The PvZn chromatogram (Fig. 5) contains all the detected peaks, except for **XVI**; however, many of them have low intensity. In the PvFe chromatogram, the **XV** peak contains only methylsulochrine.

**Fig. 6:**
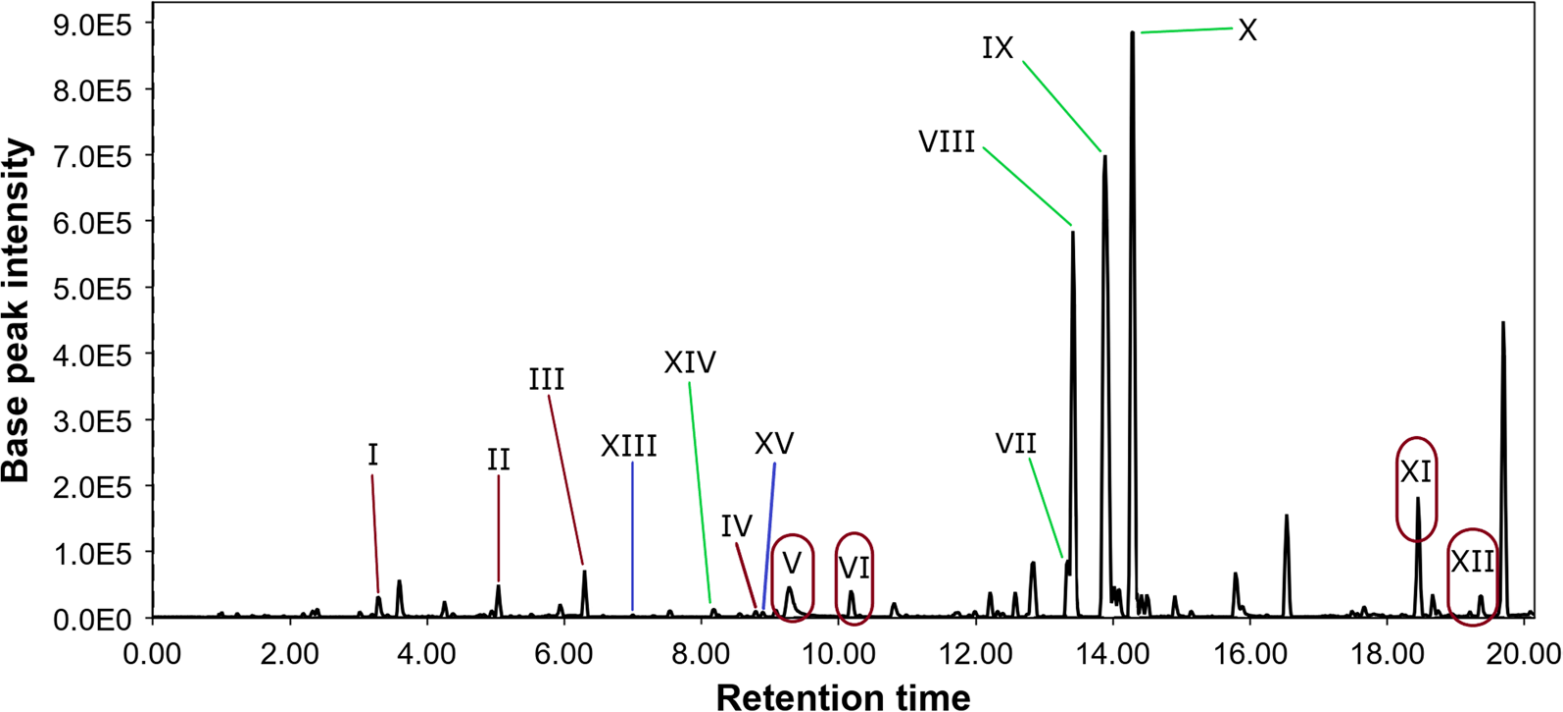
LC/MS chromatogram of the PvNi extract

The PvNi chromatogram (Fig. 6) differed from that of the control owing to the presence of additional low-intensity peaks **XIII, XIV,** and **XV**. The intensity of the other peaks in the PvNi chromatogram increased by two times compared to that of the Pv0 chromatogram.

**Fig. 7:**
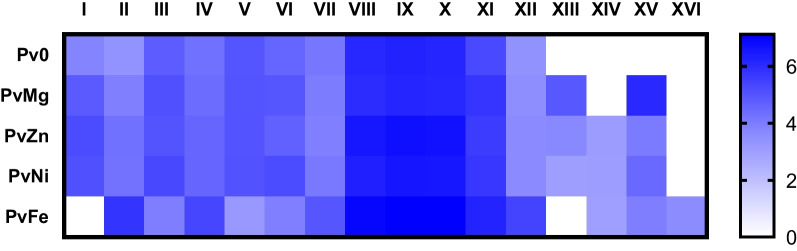
Heat map of the relative content of compounds identified in fungal extracts. Each cell shows the decimal logarithm value of the relative area of the LC/MS peak

To correlate the content of the identified compounds in all analyzed extracts according to the LC/MS profile, a heat map was built and is presented in Fig. 7. The peaks with highest intensity are the peaks **VIII-X**. The largest total area of peaks was observed in extract PvFe and this extract is very different from the others.

**Fig. 8:**
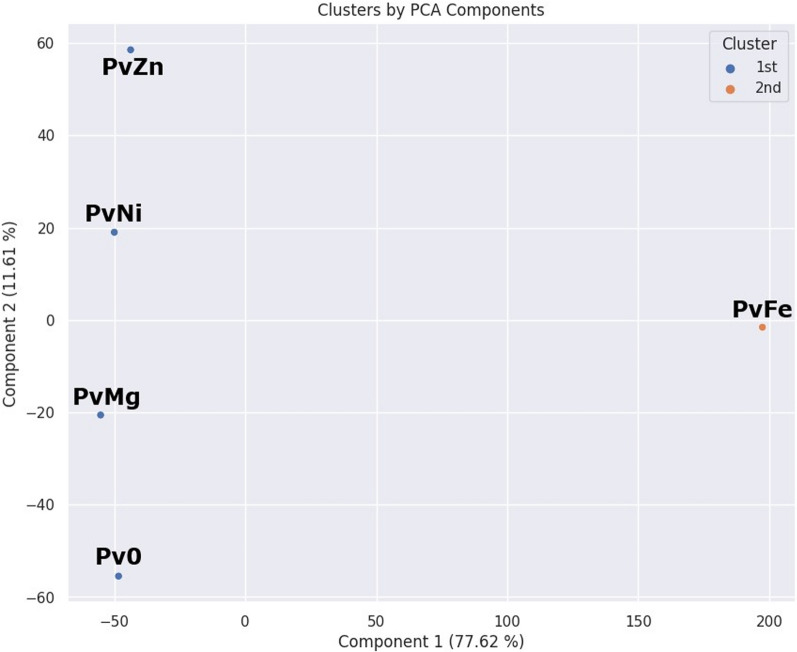
The principal component analysis (PCA) of the fungal extracts. PCA model was created to estimate the distribution of extracts based on the metabolites found in each extract

Principal component analysis (PCA) was used to identify statistical differences in the data between the extracts (Fig. 8). The analysis was performed on all peaks of the LC/MS profiles. To visualize the PCA model, we decided to use two components that described 89.23% of the variance in the extracts.

To highlight the differences between the extracts, we applied cluster analysis using the k-means method. Pv0, PvMg, PvZn, and PvNi were found to be similar to each other, which is consistent with the LC/UV and LC/MS profiles, whereas the PvFe extract occupied another position.

### The bioactivity of extracts *P. velutinum*

The cytotoxic activity of all purified ethyl acetate extracts obtained using the OSMAC strategy toward human prostate cancer PC-3 and human embryonic kidney HEK-293 cells, as well as its effect on yeast-like fungi *Candida albicans* culture growth and DPPH radical scavenging activity, were assayed (Table 1).

Pv0 extract decreased the viability of prostate cancer PC-3 and normal HEK-293 cells by 47.3% and 65.1%, respectively. The effects of PvMg, PvZn, and PvNi extracts on cell viability were similar to those of Pv0. Only the PvFe extract exhibited lower cytotoxicity and decreased the viability of PC-3 and HEK-293 cells by 19.6% and 17.9%, respectively. Pv0 extract inhibited the growth of *C. albicans* culture by 42.8%. PvMg, PvFe, and PvNi extracts inhibited the growth of *C. albicans* culture by 45.3%, 41.5%, and 37.5%, respectively, similar to Pv0. PvZn was less effective in this test. Pv0 extract scavenged half of DPPH radicals at 34.9 µM and PvMg, PvZn, and PvNi make it at 40.0 µM, 54.8 µM, and 49.1 µM, respectively. PvFe extract was less effective in this test, and scavenged half of the DPPH radicals at 82.1 µM.

Thus, the changing the conditions of the cultivation of the fungus *P. velutinum* significantly affected the metabolite profile as well as biological activity of the fungal extracts. The results of both PCA and bioassays allowed us to choose a variant of fermentation of the fungus with the addition of Fe(NO_3_)_3_·9H_2_O for preparative cultivation and the isolation of individual compounds.

**Fig. 9:**
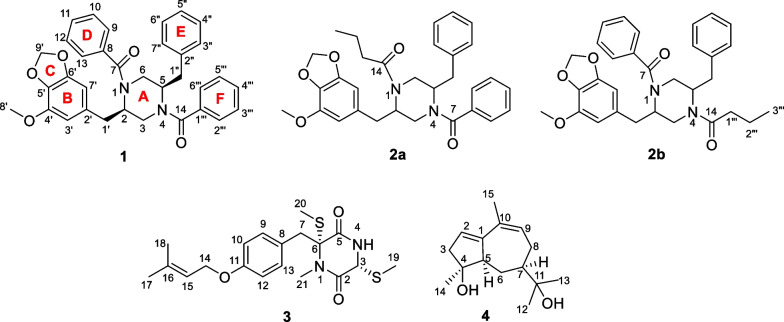
Metabolites isolated from *Penicillium velutinum* ZK-14

### Structure determination of the individual compounds from the EtOAc extract ***P. velutinum*** ZK-14 cultivated with Fe(NO_3_)_3_·9H_2_O

**Fig. 10:**
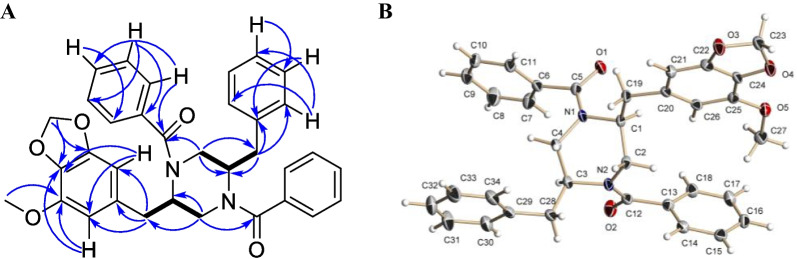
**A** The key COSY (bold lines) and HMBC (arrows) correlations of **1. B** Single-crystal X-ray diffraction structure of **1**, with displacement ellipsoids drawn at the 50% probability level

The molecular formula of **1** was established to be C_34_H_32_N_2_O_5_ from a HRESIMS peak at *m/z* 547.2215 [M *–* H]^−^ and *m/z* 571.2193 [M + Na]^+^ with 20 degrees of unsaturation. The ^13^C and ^1^H NMR data (Table 2) of **1** were similar to those of the known piperazine derivative helvamide [39] and iizukine E [40] except for the absence of the H-1' *sp*^2^ methine proton and *sp*^2^ C-2 carbon signals, together with the absence of the methyl group C-15 compared to the spectral data of the helvamide and iizukine E. However, in the ^13^C and ^1^H NMR data, including HSQC and DEPT-135 experiments, of the compound **1** showed the presence of the C-1' (*δ*_C_ 37.5, *δ*_H_ 2.83, *δ*_H_ 2.77) methylene and C-2 (*δ*_C_ 55.7, *δ*_H_ 4.43) methine groups (Table 3, Fig. 10A) in **1**. The COSY correlations (Fig. 10) from H-2 (*δ*_H_ 4.43) to H-3b (*δ*_H_ 3.07), H-1’a (*δ*_H_ 2.81), and H-1’b (*δ*_H_ 2.77) confirmed the absence of the ∆^1,2^ double bond in **1**. Moreover, the ^1^H NMR spectra contain signals of the 15 protons in the range *δ*_H_ 7.11–7.45 ppm that corresponds to aromatic (phenyls). Thus, this information and the HMBC correlations from *δ*_H_ 7.31 (that corresponding to four protons: H-9/H-13 and H-2'''/H-6''') to C-7 (*δ*_C_ 171.7) and C-14 (*δ*_C_ 171.7) confirmed the presence of a new phenyl at C-14 (ring F in Fig. 9). These data were confirmed by the difference of 64 mass units between **1** and helvamide and iizukine E.

**Table 2:** ^13^C and ^1^H NMR data for compounds **1** and** 2** in acetone-*d*_6_

No	1^a^		2a/2b^b^	
	*δ*_C_, type	*δ*_H_, mult. (*J* in Hz)	*δ*_C_, type	*δ*_H_, mult. (*J* in Hz)
2	55.7, CH	4.43, brs	56.4, CH	4.34, brs
3	44.1, CH_2_	3.81, brm 3.07, dd (14.3, 10.8)	43.9, CH_2_^c^	Overlapped
5	55.6, CH	4.50, brs	54.4, CH	4.41, brs
6	44.1, CH_2_	3.86, brm 3.14, dd (14.3, 11.0)	43.9, CH_2_^c^	3.90, m 3.18, m
7	171.7, C	–	171.6, C	–
8	137.5, C	–	137.5, C	–
9/13	127.5, CH	7.31, d (7.1) (2H)	129.3, CH	7.28, m
10/12	129.3, CH	7.40, m (2H)	129.4, CH	7.39, m
11	130.2, CH	7.42, m	129.3, CH	7.24, m
14	171.7, C	–	174.6, C	–
1'	37.5, CH_2_	2.83, td (13.5, 5.1) 2.77, dd (13.5, 7.8)	36.8, CH_2_	2.78, brs 2.75, brs
2'	132.4, C	–	132.3, C	
3'	109.6, CH	6.29, brs	109.9, CH	6.41, brs
4'	144.6, C	–	144.7, C	–
5'	134.9, C	–	135.0, C	–
6'	149.9, C	–	149.9, C	–
7'	103.9, CH	6.26, brs	104.1, CH	6.31, brs
8'	56.8, CH_3_	3.78, s	57.0, CH_3_	3.80, s
9'	102.1, CH_2_	5.93, d (1.1) 5.92, d (1.1)	102.1, CH_2_	5.92, brs (2H)
1''	37.5, CH_2_	2.95, td (13.5, 5.0) 2.90, dd (13.5, 8.0)	37.8, CH_2_	2.96, brs (2H)
2''	138.1, C	–	138.2, C	–
3''/7	130.3, CH	7.11, d (7.1) (2H)	130.4, CH	7.16, brs
4''/6''	129.3, CH	7.23, m (2H)	130.2, CH	7.42, m
5''	127.4, CH	7.20, m	127.4, CH	7.24, m
1'''	137.6, C	–	30.3, CH_2_	Overlapped
2'''	127.5, CH	7.31, d (7.1)	19.5, CH_2_	Overlapped
3'''	129.3, CH	7.40, m	20.5, CH_3_	1.02, brs
4'''	130.3, CH	7.42, m	–	–
5'''	129.3, CH	7.40, m	–	–
6'''	127.5, CH	7.31, d (7.1)	–	–

^a^Measured at 75 and 300 MHz; ^b^ Measured at 175 and 700 MHz; ^c^ low-intensity signals adjacent to nitrogen atoms

**Fig. 11:**
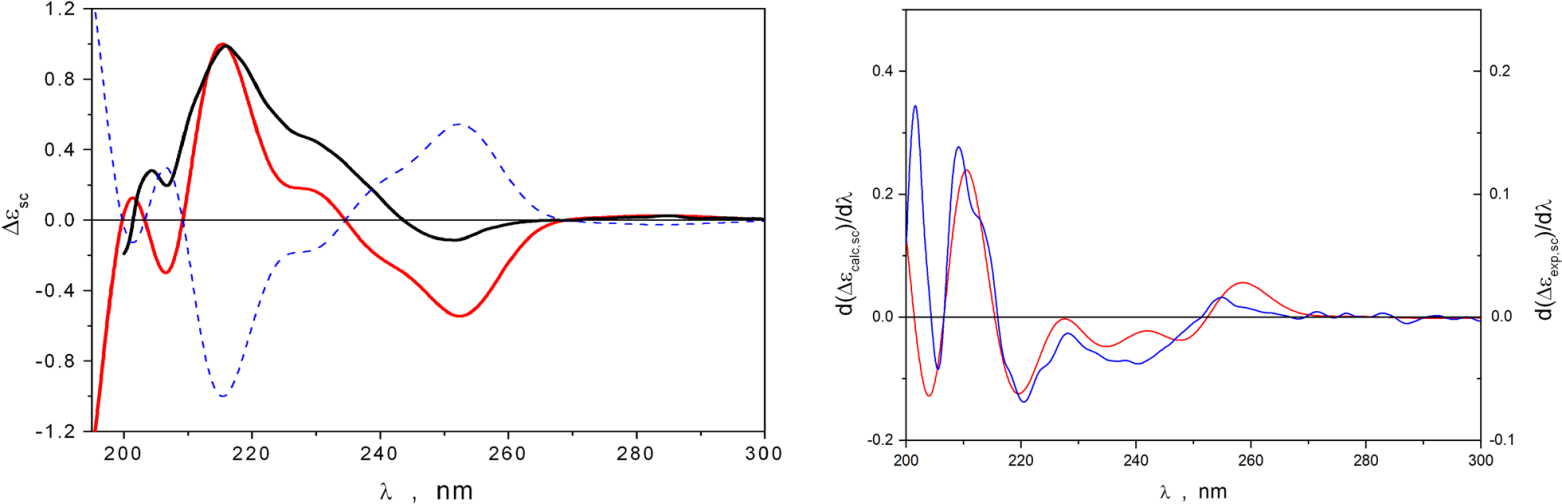
**A** ECD spectra calculated for different conformations of **1**. Red line** –** calculated for *R,R*-**1**, blue dotted line– calculated for *S,S*-**1**, black line – experimental. **B** The first derivative functions d(Δε_exp_(λ))/dλ and d(Δε_calc_(λ))/dλ for *R,R*-**1**. Red line– calculated, blue line—experimental

The planar structure and relative configurations of the stereogenic centers of **1** were unequivocally determined by X-ray analysis, which was carried out for a single crystal obtained by recrystallization from MeOH – H_2_O (70:30) (Fig. 10B, Additional file 1: Fig. S25). Flack parameter and uncertainty was relatively high because of the use of MoKa radiation for data collection on a structure with no heavy atoms, so the absolute configurations of the stereogenic centers of **1** were established by standard approach – via comparison of calculated (averaged over conformational rearrangements) ECD spectra of different stereoisomers with the experimental ECD spectrum of **1**. The performed conformational analysis allowed the selection of eight most stable conformations, for which ECD spectra were then calculated and averaged using corresponding statistical weights. A comparison of the ECD spectra, calculated for the *R,R*- and *S,S*- stereoisomers of **1**, with the experimental ECD spectrum is presented in Fig. 11A. The experimental ECD spectrum of **1** contained two characteristic bands: a broad intensive positive band in the region 210 ≤ λ ≤ 245 nm and a less intensive negative band in the region 245 ≤ λ ≤ 265 nm. The theoretical ECD spectrum for *R,R*-1 represents the main qualitative features of the experimental spectrum well, which can be confirmed by comparing the properties of the first derivative’s functions d(Δε_exp_(λ))/dλ and d(Δε_calc_(λ))/dλ (Fig. 11B). Thus, the minima and maxima of both functions are realized at nearly the same λ values.

**Fig. 12:**
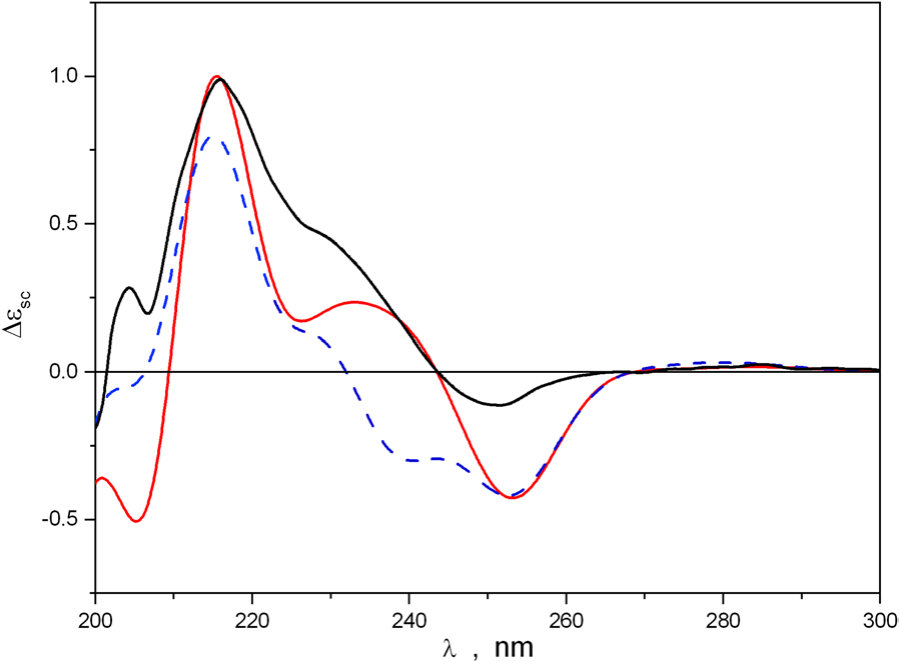
The ECD spectra for two main conformations of *R,R*-**1**, compared with experimental ECD spectrum of **1**. Dotted blue line – calculated for *R,R*-1-i1rc, red line – calculated for *R,R*-1-i1c (Additional file 1: Fig. S27), black line – experimental

The intensities of the positive bands in the 195 ≤ λ ≤ 210 nm and 230 ≤ λ ≤ 245 nm regions were, to some extent, underestimated, perhaps, due to the insufficiency of the PCM approach, used for modeling the interaction of **1** with the solvation shells. Thus, the performed analysis showed that a shoulder on the long-wave side of the positive band in the experimental ECD spectrum (230 ≤ λ ≤ 245 nm) was caused by contributions to the total spectrum, which were obtained from the conformations of the 1-i1c and 1-i1t types (Additional file 1: Fig. S26). In addition, conformations 1-i1rt and 1-i1rc generate spectra in which the shoulder on the positive band is absent, leading to a large bandwidth of the negative band in the region 230 ≤ λ ≤ 265 nm. The structure of **1** is very flexible owing to its many large-amplitude motions (LAM), which can proceed simultaneously (Fig. 12).

Conformational analysis showed that more stable conformations (with a total amount exceeding 99%) are realized when the piperazine cycle has a boat conformation, in which hydrogen atoms at C(2) and C(5) have axial orientations. The most stable of them were 1-i1c, 1-i1t, 1-i1rc and 1-i1rt, which total amount is ≈ 86% (Additional file 1: Fig. S27). According to the data obtained from the X-ray experiments, the crystal under study was built with molecules that exist exclusively in the 1-i1t conformation (and its variant 1-i1t-2). In the gaze phase and in the solutions, a large number of other conformations may be realized, and the inversion of the nitrogen-containing six-ring may also occur, leading to conformations of the 1-i2 type (Additional file 1: Fig. S28).

According to performed calculations, the amounts of {1-i1c, 1-i1t}and {1-i1rc, 1-i1rt} conformations relay approximately as 2:3. It seems that amounts of {1-i1c, 1-i1t} conformations relative to {1-i1rc, 1-i1rt} conformations, calculated in the framework of the PCM approach, are underestimated to some extent. Nevertheless, based on all obtained experimental and experimental data, we can conclude that the absolute configuration of the compound under study is *R,R*-**1**. Thus, the structure of **1** was determined and named helvamide B.

The molecular formula of **2** was established to be C_31_H_34_N_2_O_5_ from a HRESIMS peak at *m/z* 513.2405 [M *–* H]^−^ and *m/z* 537.2358 [M + Na]^+^ with 16 degrees of unsaturation. The ^13^C and ^1^H NMR data (Table 2) of **2** were similar to those of **1,** except for the presence of 12 aromatic proton signals in **2** compared with the 17 aromatic proton signals in **1**. Unfortunately, there were no signals of H_2_-3, H_2_-1′′′, and H_2_-2′′′ in the ^1^H NMR spectrum, as well as the absence of HMBC cross-peaks from H-3/H-6 to C-7/C-14; therefore, it was not possible to unambiguously determine the position of butyryl and benzoyl substituents at the nitrogen atoms of the piperazine ring. The 2D NMR spectra of **2** were not informative because of the breadth of most signals, which negatively affected the accumulation of the HMBC and COSY correlations. The presence of butyryl and benzoyl substituents in compound **2** was suggested by fragmentation of the cationized molecule at *m/z* 537 in CID MS (tandem mass spectrometry) (Fig. S11 and S12). The presence of the N-butyryl substituent was confirmed by the appearance of fragment ion peaks at *m/z* 301.1330 in the MS/MS of the cationized molecule at *m/z* 537.2354, which resulted from the cleavage of the N(4)–C(14) or N(1)–C(14) bond (Additional file 1: Figs. S11 and S12, Fig. 9).

**Fig. 13:**
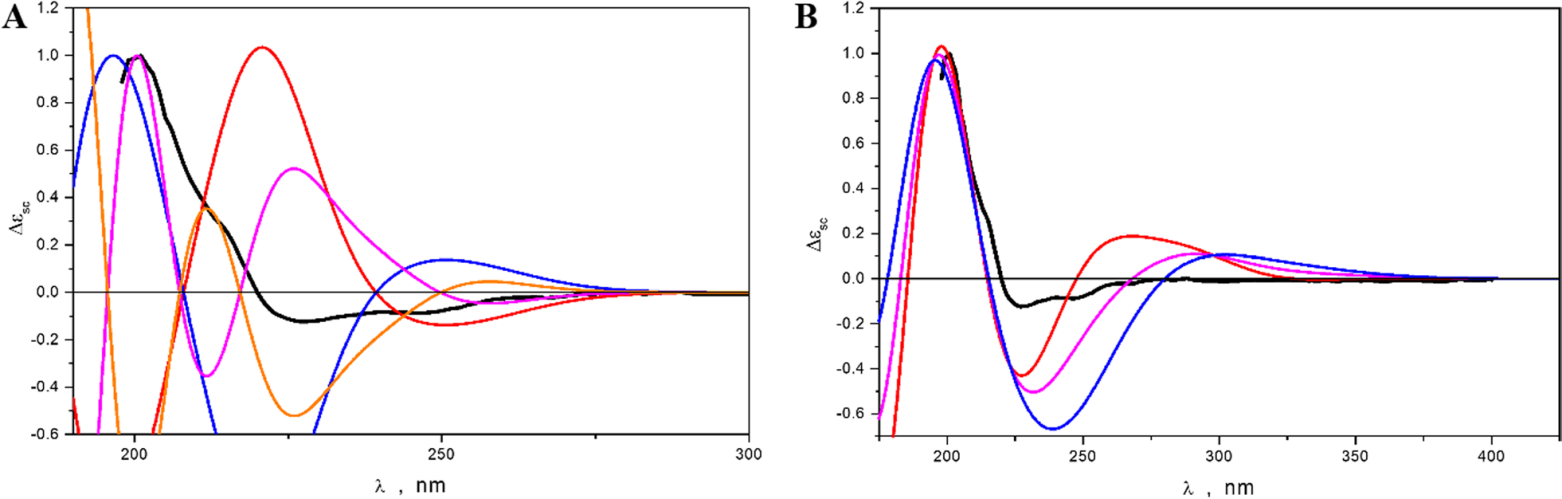
**A** ECD spectra, calculated for pure enantiomers of *R*,*R*-**2a** (red), *S*,*S*-**2a** (blue), *R*,*R*-**2b** (pink), *S*,*S*-**2b** (orange), and experimental ECD spectrum (black) of **2**.** B** ECD spectra, simulated for mixtures of the *R*,*R*-**2a** and *S*,*S*-**2b** isomers (red – 70:30 ratio, pink – 50:50, blue – 30:70), compared to the experimental ECD spectrum (black) of **2**

Quantum chemical calculations of the ECD spectra were used to accurately determine the position of the N-substituents, as well as the stereo configuration of the piperazine ring. Conformational analysis of compound **2** showed that the prevailing conformation of the nitrogen-containing cycle is the “boat-1” conformation, in which the hydrogen atoms H-2 and H-5 remain in the axial position. The most stable conformations of **2** realize when the carbonyl groups C-7 and C-14 are oriented toward neighboring amino acid residues. Both “chair-type” conformations and the “boat-2” conformation, in which atoms H-2 and H-5 stay in equatorial orientation loses to most stable “boat-1” conformation for about ∆G ≥ 3 kcal/mol. For further investigations, we selected four main conformations for each isomer (Additional file 1: Fig. S29). The calculated ECD spectra of enantiomers **2a** and **2b** compared with the experimental ECD spectrum of **2** are presented in Fig. 13A. The theoretical ECD spectra of the pure stereoisomers poorly described the shape of the experimental spectrum. It was assumed that the sample under study was composed of a mixture of different stereoisomers. The most appropriate result, in our opinion, achieves when *R,R*-**2a** is mixed with *S,S*-**2b** in any proportion. The theoretically simulated ECD spectra for this case are presented in Fig. 13B. The obtained theoretical spectra are in better qualitative agreement with the experimental spectra.

For the first time, compounds with a 2,5-dibenzylpiperazine moiety and named brasiliamides were isolated from the fungus *Penicillium brasilianum* Batista JV-379 [41, 42]. In addition, such compounds were isolated from *Aspergillus nidulans* BF-0142 [39] and *Aspergillus iizukae* [40]. The biosynthetic source of 2,5-dibenzylpiperazine derivatives is L-phenylalanine [43].

The molecular formula of **3** was established to be C_19_H_26_N_2_O_3_S_2_ from a HRESIMS peak at m/z 393.1313 [M – H]^–^ with eight degrees of unsaturation. This information and close inspection of the ^13^C and ^1^H NMR spectra (Additional file 1: Table S2), including HSQC, HMBC, COSY, and DEPT experiments, established compound **3** as a known thio-diketopiperazine saroclazin A (Fig. 1) [44]. This compound was previously isolated from the mangrove-derived fungus *Sarocladium kiliense* HDN11-84 [44] and possesses a free amide group, which was first found in sulfur-containing aromatic diketopiperazine derivatives (DKPs).

The molecular formula of **4** was established to be C_15_H_24_O_2_ from a HRESIMS peak at *m/z* 235.1701 [M – H]^−^ with four degrees of unsaturation. This information and close inspection of the ^13^C and ^1^H NMR spectra (Additional file 1: Table S3), including HSQC, HMBC, COSY, and DEPT experiments, established compound **4** as a known terpene (4*S*,5*R*,7*S*)-4,11-dihydroxy-guaia-1(2),9(10)-dien *(*Fig. 1*)* [45]. A terpene featuring a 1(2),9(10)-conjugated double bond earlier was isolated from the stems of *Solanum erianthum *[45] and the flower buds of *Daphne genkwa* [46]. According to the literature, no terpenes have been isolated from the fungus *Penicillium velutinum* or other fungi of the section *Exilicaulis* [26–28].

Helvamide B (**1**) was detected as peak VII in the LC/MS chromatogram of all investigated fungal culture extracts, but the PvFe extract had the highest amount of this new compound. Helvamide C (**2**) may be detected with peak IX in HPLC MS of all investigated fungal culture extracts, and the PvFe extract had the highest amount of **2**. Helvamides B (**1**) and C (**2**) were detected in all fungal extracts, but their relative amounts may change as Pv0 ≤ PvMg < PvZn ≤ PvNi < PvFe and Pv0 ≤ PvMg < PvNi ≤ PvZn < PvFe, respectively. It should be noted that Mg^2+^ and Zn^2+^ have been added to the cultivation media as chlorides, while Ni^2+^ and Fe^3+^ have been added as nitrates. Therefore, both Fe(NO_3_)_3_ and Ni(NO_3_)_2_ may be sources of nitrogen to enhance the production of nitrogen-containing helvamides B (**1**) and C (**2**). Moreover, an increase in the relative amounts of nitrogen-containing compounds was detected in the VIII and XI peaks in the PvFe extract. Also the nitrogen-containing thio-diketopiperazine alkaloid saroclazine A (**3**) was announced with peak XVI and detected only in in Fe(NO_3_)_3_·9H_2_O treated fungal culture. These findings led to the assumption that this strain is capable of producing a variety of alkaloids in sufficient quantities if sufficient nitrogen sources are present in the nutrient medium. Compound (4*S*,5*R*,7*S*)-4,11-dihydroxy-guaia-1(2),9(10)-dien (**4**) was announced with peak XIV and was detected only in PvZn, PvNi and PvFe extracts with ZnCl_2,_ Ni(NO_3_)_2_·6H_2_O, and Fe(NO_3_)_3_·9H_2_O, respectively. Moreover, fallacinol was announced with peak XIII only in the PvMg, PvZn, and PvNi extracts. In addition, sulochrin and methylsulochrin were announced with peak XV only in the PvMg, PvZn, PvNi, and PvFe extracts.

The metabolite profile of *P. velutinum* was studied for the first time in the present work, and this fungal strain was found to be a source of various alkaloids. Moreover, the change in cultivation media and the addition of the four new components caused variations in the metabolite profiles of the new extracts. This observation is fully consistent with numerous results from other research teams. For example, a new anticancer caryophyllene sesquiterpenes 6-O-demethylpestalotiopsin A and 6-O-demethylpestalotiopsin C with a five-membered hemiacetal structural moiety were isolated from the marine-derived fungus *Ascotricha* sp. ZJ-M-5 cultivated with MgCl_2_ using the OSMAC strategy [47]. The antibiotic (1*S*,6*S*)-3-((3*S*,4*R*,5*E*,7*E*)-8-cyclopropyl-3-hydroxy-6-methyl-2-oxoocta-5,7-dien-4-yl)-7-oxa-3-azabicyclo[4.1.0]heptan-2-one was isolated from a Ni-stressed *Streptomyces* sp. WU20 [48], and the authors reliably demonstrated the absence of this metabolite in the control extract.

Fungal response to metal stress includes several aspects as extracellular response upon metal toxicity and intracellular processes and transport [49]. These processes are aimed at both directly binding and removing excess amounts of metals from the cell and responding to metal-induced oxidative stress [50]. Earlier, 3-methyl-5,6(7),8-trihydroxy-2-aza-anthraquinone tolypocladin as ion chelator was isolated from *Tolypocladium inflatum* DSM 915 fungus and its production was Zn-dependent [51]. The study of isolates of *Pyrenophora avenae* led to the observation that there is a correlation between the production of 1,4,5,8-tetrahydroxyanthraquinones by different isolates and their resistance to phenylmercuric acetate. Moreover, the authors showed that these pigments removed phenyl-Hg^+^ ions from aqueous solution [52]. Sulochrin and related benzophenones ( detected in the extracts of metal-treated *P. velutinum*) have been reported as radical scavengers and antioxidants [53]. This indicates that the production of these compounds by the fungus can occur because of activation of the metal defense system.

Therefore, the addition of Fe(NO_3_)_3_·9H_2_O to fungal culture media caused a significant increase in the production of nitrogen-containing secondary metabolites, making it possible to isolate the compounds in sufficient amounts and study their chemical structures and bioactivities.

### Bioactivity of isolated compounds

#### The cytotoxic and antimicrobial activity

The effects of compounds **1***–***4** on the viability of human prostate cancer cells PC-3 and normal human embryonal kidney cells HEK-293, as well as their influence on *C. albicans* growth and DPPH radical scavenging effect were evaluated, and the data are presented in Table 3.

**Table 3:** The cytotoxic and antimicrobial activities of the isolated compounds

Compounds	Cell viability, % of control		Inhibition of *C. albicans* growth, %	DPPH radical scavenging activity,
	PC-3	HEK-293		%
**1**	65.97 ± 1.62	83.36 ± 0.39	9.73 ± 1.02	4.92 ± 0.50
**2**	60.59 ± 1.62	71.58 ± 2.13	31.54 ± 1.66	4.54 ± 0.51
**3**	85.14 ± 1.38	100	12.31 ± 0.12	–
**4**	95.05 ± 2.75	100	7.12 ± 0.89	–

The concentrations of the compounds were 100 μM. All data are presented as a mean ± standard error of mean (SEM)

Compounds **3** and **4** did not influence on HEK-293 cell viability and had no statistically significant effect on PC-3 cell viability. Compound **1** decreased the viability of PC-3 cells by 34.03%, while the viability of HEK-293 was diminished only by 16.64%. Compound **2** decreased the viability of PC-3 cells by 39.41% and HEK-293 cells by 28.42%.

The radical scavenging activities of the isolated compounds **1***–***4** at a concentration of 100 µM were assayed. Compounds **1** and** 2**, statistically significantly scavenged the DPPH radicals by 4.92% and 4.54%, respectively. The other isolated compounds were inactive in this test.

Compounds **1, 3**, and **4** inhibited the growth of *C. albicans* test-culture by approximately 7–12%, whereas compound **2** significantly inhibited the growth of these yeast-like fungi by 31.54%. The yeast-like fungus *Candida albicans*, a nosocomial infection, significantly complicates the treatment of other diseases and recovery after surgical interventions. Data on new anti-candidosis compounds with low toxicity to human cells are promising.

## Materials and methods

### General experimental procedures

Optical rotations were measured on a Perkin-Elmer 343 polarimeter in MeOH. UV spectra were recorded on a Shimadzu UV-1601PC spectrometer in MeOH. ECD spectra were measured on a Chirascan-Plus CD Spectrometer in MeOH. ^1^H and ^13^C NMR spectra were recorded in aceton-d_6_ on a Bruker Avance-300, Avance-500 and Avance III-700 spectrometers (Bruker BioSpin GmbH) operating at 300 and 75, 500 and 125 MHz and 700 and 176 MHz, respectively. HRESIMS spectra were obtained on a Bruker maXis Impact II mass spectrometer (Bruker Daltonics GmbH). Low-pressure liquid column chromatography was performed using C_18_-SiO_2_ Gel ODS-A (12 nm, S—75 um, YMC Co., Ishikawa, Japan) and a Buchi B-688 Chromatography Pump on a Buchi glass column using Si gel KSK (50/100 μm, Imid Ltd., Russia). Plates precoated with Si gel (5–17 μm, 4.5 × 6.0 cm, Imid Ltd., Russia) and Si gel 60 RP-18 F254S (20 × 20 cm, Merck KGaA, Germany) were used for thin-layer chromatography. Preparative HPLC was carried out on a Shimadzu LC-20 (Shimadzu, Kyoto, Japan) and Agilent 1100 (Agilent Technologies, Santa Clara, CA, USA) chromatographs using a Shimadzu RID-20A and Agilent 1100 refractometers and YMC ODS-AM (YMC Co, 5 μm, 250 × 10 mm), Ultrasphere Si (5 μm, 250 × 4.6 mm), and Hydro-RP (Phenomenex, 4 μm, 250 × 10 mm) columns.

### Fungal strain

The fungal strain ZK-14 was isolated from the superficial mycobiota of the rhizome seagrass *Zostera marina* (the Sea of Japan) and stored in the Collection of Marine Microorganisms (PIBOC FEB RAS, Vladivostok, Russia).

The ZK-14 strain was identified as *Penicillium velutinum* based on four molecular genetic markers: ITS, beta-tubulin (*BenA*), calmodulin (*CaM*), and RNA polymerase II second largest subunit (*RPB2*) regions/gene sequences. BLAST analysis showed that the strain ZK-14 was 100% similar to *Penicillium velutinum* NRRL2069 in the GenBank. The sequences were deposited in the GenBank database under accession numbers OQ427361 for ITS and OQ466610 for partial β-tubulin.

### DNA extraction and amplification

Genomic DNA was isolated from fungal mycelia (mycelium) grown on malt extract agar (MEA) at 25 °C for 7 days using the MagJET Plant Genomic DNA Kit (Thermo Fisher Scientific, Waltham, MA, USA), according to the manufacturer’s protocol. PCR was conducted using GoTaq Flexi DNA Polymerase (Promega, Madison, WI, USA). For amplification of the ITS region were used the primer pair 1400-F (5’-CTGCCCTTTGTACACACCGCCCGTC-3’) [54] and D3B*-R (5’- ACTTCGGAGGGAACCAGCTAC-3’) [55]. The reaction profile was 95 °C for 300 s, 35 cycles of 94 °C for 20 s, 60 °C for 20 s, and 72 °C for 90 s, and finally 72 °C for 300 s. For amplification of the partial *BenA* gene were used the standard primer pair Bt-2a and Bt-2b [56]. The reaction profile was 95 °C for 300 s, 35 cycles of 94 °C for 20 s, 55 °C for 20 s, and 72 °C for 60 s, and finally 72 °C for 300 s. For amplification of the partial *CaM* gene were used the degenerate primer pair cal_P/A_F (5’-TCYGAGTACAAGGAGGCSTT-3’) and cal_P/A_R (5’-CCRATGGAGGTCATRACGTG-3’). The reaction profile was 95 °C for 300 s, 35 cycles of 95 °C for 20 s, 60 °C for 30 s, and 72 °C for 90 s, and finally 72 °C for 300 s. For amplification of the partial *RPB2* gene were used the degenerate primer pair rpb2_Pen_F (5’-GAGACYAAYCGBGARATYTA-3’) and rpb2_Pen_R (5’-GTCATSACAATCATRATDGT-3’). The reaction profile was 95 °C for 300 s, 35 cycles of 95 °C for 20 s, 50 °C for 30 s, and 72 °C for 90 s, and finally 72 °C for 300 s. The amplified *ITS*, *BenA, CaM* and *RPB2* genes were purified with the ExoSAP-IT™ PCR Product Cleanup Reagent (Thermo Fisher Scientific, Waltham, MA, USA). Sequencing was bidirectional performed with the same primers on an Applied Biosystems SeqStudio Genetic Analyzer (Thermo Fisher Scientific, Waltham, MA, USA) using the Big Dye Terminator reagent kit, version 3.1. Gene sequences were deposited in GenBank under the accession numbers OQ427361 for ITS, OQ466610 for the partial *BenA*, OR356207 for the partial *CaM* and OR356208 for the partial *RPB2* (Additional file 1: Table S1).

### Phylogenetic analysis

The ITS region, the partial *BenA*, *CaM* and *RPB2* gene sequences the fungal strain ZK-14 and members of genus *Penicillium* section *Exilicaulis*, series *Lapidosa* were aligned by MEGA X software version 11.0.9 [57] using the Clustal W algorithm. The ex-type homologs were searched in the GenBank database (http://ncbi.nlm.nih.gov) using the BLASTN algorithm (http://www.ncbi.nlm.nih.gov/BLAST, accessed on 20 February 2024). The phylogenetic analysis was conducted using MEGA X software [57]. The ITS region and partial *BenA*, *CaM,* and *RPB2* gene sequences were concatenated into one alignment. A phylogenetic tree was constructed according to the Maximum Likelihood (ML) algorithm based on the Kimura 2-parameter model [58]. The tree topology was evaluated by 1000 bootstrap replicates. *Talaromyces marneffei* CBS 388.87^T^ was used in the phylogenetic analysis as an outgroup (Additional file 1: Table S1).

### Molecular identification of the fungal strain

The strain ZK-14 was identified using molecular markers such as ITS and partial *BenA, CaM,* and *RPB2* regions. Approximately 1600 bp fragment of the ITS region, about 500 bp fragments of the partial *BenA* and *CaM* regions, and a 770 bp fragment of the partial *RPB2* gene were successfully amplified. BLAST search showed that the ITS and partial *BenA* regions sequences were 100% identical with the sequences of the ex-type strain *Penicillium velutinum* NRRL 2069, whereas partial *CaM* and *RPB2* genes were more than 99% identical. The phylogenetic ML tree of the concatenated ITS-*BenA*-*CaM-RPB2* gene sequences clearly showed that the strain ZK-14 clusters with the ex-type strain *Penicillium velutinum* NRRL 2069 (Additional file 1: Fig. S1).

### Cultivation of fungus *Penicillium velutinum*

The fungal strains were cultured for 21 days at 22 °C in Erlenmeyer flasks (500 mL) for each condition on the rice medium (RM) and various amounts of natural sea salt or metal ions (the fungus was cultured in two flasks for each culture condition). RM containing rice (20.0 g, white round-grain polished rice *Oryza sativa* grade extra "Japonka", Primorsky region, Russia), yeast extract powder (0.02 g, Himedia RM027, HiMedia Laboratories LLC., India), KH_2_PO_4_ (0.01 g, Lenreactiv Ltd., Russia), KNaC_4_H_4_O_6_*4H_2_O (0.01 g, Reakhim Ltd., Russia), natural seawater (40 mL, Vodolaznaya bay, Troitsa bay, the Sea of Japan, September 2022), metal salt (100 μM) [48]. The natural salinity of used water was 37.8 g/L. ZnCl_2_ (Lenreactiv Ltd., Russia), Ni(NO_3_)_2_·6H_2_O (Lenreactiv Ltd., Russia), Fe(NO_3_)_3_·9H_2_O (Lenreactiv Ltd., Russia), MgCl_2_·6H_2_O (Lenreactiv Ltd., Russia). The fermentation conditions are listed in Table 4. Control of fungus was cultivated on the RM.

**Table 4:** Cultivation conditions for *Penicillium velutinum* ZK-14

No	Cultivation conditions	Extract code	Number of Erlenmeyer flasks	Diametric growth rate of a fungus colony, mm (7/14 days)	Crude extract amount, mg	Purified fraction amount, mg
1	RM (control of fungus)	Pv0	2	57/89	399.0	279.3
2	RM + MgCl_2_·6H_2_O (100 μM)	PvMg	2	54/90	503.0	472.3
3	RM + Fe(NO_3_)_3_·9H_2_O (100 μM)	PvFe	2	54/90	813.4	746.0
4	RM + ZnCl_2_ (100 μM)	PvZn	2	53/90	523.3	371.9
5	RM + Ni(NO_3_)_2_·6H_2_O (100 μM)	PvNi	2	53/90	396.0	270.1

### Extraction of the *Penicillium velutinum* and extract preparation

At the end of the incubation period, each fungal culture, together with the medium, was extracted with EtOAc (2 × 100 mL). The obtained extracts were filtered, concentrated to dryness using a rotary evaporator, and then weighed (Table 4). For the obtained crude extracts, extract preparation was carried out for further LC/UV analysis. The dry crude extract residues were dissolved in methanol (30 mL) and purified by flash column chromatography in 100% methanol (total volume of 250 mL for each extract). Low-pressure liquid column chromatography was performed using an empty glass column (2 × 7 cm) and the Gel ODS-A (Table 4).

### LC/UV

Analysis was performed using an Agilent 1260 Infinity II chromatograph with UV detector Agilent 1260 VWD (Agilent Technologies, USA) using column YMC ODS-AM C18 (YMC Co, 5 μm, 250 × 10 mm). The mobile phases were H_2_O (eluent A) and MeCN (eluent B). The gradient program was as follows: from 0 to 100% eluent B from 0 to 60 min. Chromatographic separation was performed at a 1.5 mL/min flow rate at 40 °C. The extract concentration was 30 µg/µL. The injection volume was 80 μL. The detection was performed at 220 and 290 nm. A qualitative and quantitative analysis of LC/UV chromatograms was carried out using Agilent OpenLab software (version 2.4). Obtained LC/UV data were exported into MS Excel software and calculated total area peaks and their number. Peaks with a relative area of less than 1% were considered noise and were eliminated.

### LC/MS

Analysis was performed using Bruker Elute UHPLC chromatography (Bruker Daltonics, Bremen, Germany) connected to a Bruker Impact II Q-TOF mass spectrometer (Bruker Daltonics, Bremen, Germany). InfinityLab Poroshell 120 SB-C18 column (2.1 × 150 mm, 2.7 μm, Agilent Technologies, Santa Clara, CA, USA) was used for chromatographic separation. The mobile phases were 0.1% formic acid in H_2_O (eluent A) and 0.1% formic acid in MeCN (eluent B). The gradient program was same as in previews work [59].

The mass spectrometry detection has been performed using an ESI ionization source in positive ion mode. Optimized ionization parameters for ESI were as follows: a capillary voltage of 4.5 kV, nebulization with nitrogen at 2.5 bar, and dry gas flow of 6 L/min at a temperature of 200 °C. The mass spectra were recorded within the *m/z* mass range of 50–2000 (scan time 1 s). Collision-induced dissociation (CID) product ion mass spectra were recorded in auto-MS/MS mode with a collision energy ranging from 15 eV at 100 m*/z* to 120 eV at 1500 m*/z* (an exact collision energy setting depended on the molecular masses of precursor ions). The precursor ions were isolated with an isolation width of 4 Th.

The mass spectrometer was calibrated using the ESI-L Low Concentration Tuning Mix (Agilent Technologies, Santa Clara, CA, USA). The instrument was operated using the otofControl (ver. 4.1, Bruker Daltonics, Bremen, Germany) and data were analyzed using the Data Analysis Software (ver. 4.4, Bruker Daltonics, Bremen, Germany).

LC/MS data were converted from Bruker “.d” to “mzXML” format using MSConvert 3.0 (part of ProteoWizard 3.0 package, Palo Alto, California, USA) [60] and further processing was performed with MZmine (version 2.53) [61]. The MZmine processing settings are given in Additional file 1: Fig. S24. Resulted in data was exported to GNPS by the Export/Submit module with the Merge MS/MS function for the identification of detected features.

PCA analysis, hierarchical dendrogram, and visualization of the resulting graphs were performed using the “google colab” web resource based on Python 3.8 using Pandas, Seaborn, and Matplotlib libraries. Below is a link to the notepad with the code used in the analysis (https://drive.google.com/drive/folders/120W5BaIs0p4gLrhpEnx18EY6afy85NU2).

### Separation of the extract fungus Penicillium velutinum ZK-14 cultivated with Fe(NO_3_)_3_·9H_2_O

At the end of the incubation period, the mycelia and medium were extracted with EtOAc (5 L). The obtained extract was concentrated to dryness. The residue was dissolved in H_2_O − EtOH (4:1) (300 mL) and was extracted with *n*-hexane (0.2 L × 3) and EtOAc (0.2 L × 3). After evaporation of the EtOAc layer, the residual material (7 g.) was purified via low-pressure liquid column chromatography on a Buchi B-688 Chromatography Pump using a Buchi glass column (49 × 230 mm) passed over a Si gel column, which was eluted, followed by a step gradient from 100% *n*-hexane to *n*-hexane – EtOAc (50:50) (total volume 25 L). Fractions (250 mL) were collected, dried on a rotary evaporator, weighed, and combined based on the TLC results.

The *n*-hexane – EtOAc (75:25) eluate (493 mg) was purified via low-pressure liquid column chromatography using an empty glass column (2 × 7 cm) and the Gel ODS-A with MeOH – H_2_O (90:10) to yield subfraction ZK-14 + Fe-25 (425 mg).

After then this subfraction was purified via preparative HPLC on a Shimadzu LC-20 (Shimadzu, Kyoto, Japan) chromatograph using a Shimadzu RID-20A (Shimadzu, Kyoto, Japan) refractometer on a YMC ODS-AM (YMC Co, 5 μm, 250 × 10 mm) column eluting with CH_3_CN – H_2_O (80:20) at 1.5 ml/min to yield subfractions ZK-14 + Fe-25–0 (292 mg) and ZK-14 + Fe-25–1 (224 mg).

Subfraction ZK-14 + Fe-25–0 (292 mg) was purified by low-pressure liquid column chromatography using an empty glass column (2 × 7 cm) and Gel ODS-A with EtOH – H_2_O (50:50; 100:0) to yield subfractions ZK-14 + Fe-25–0-50 (163.6 mg) and ZK-14 + Fe-25–0-100 (125.2 mg). After then ZK-14 + Fe-25–0-100 (125.2 mg) was purified via preparative HPLC on an Agilent 1100 chromatograph using an Agilent 1100 refractometer on a Hydro-RP column eluting with CH_3_CN − H_2_O (70:30) and Ultrasphera Si column eluting with toluene-isopropanol (9:1) at 1 ml/min to yield **4** (0.9 mg). Subfraction ZK-14 + Fe-25–0-50 (163.6 mg) was purified via preparative HPLC on a Shimadzu LC-20 chromatograph using a Shimadzu RID-20A refractometer on a YMC ODS-AM column eluting with CH_3_CN – H_2_O (80:20) at 1.5 ml/min to subfraction ZK-14 + Fe-25–0-50–2 (52 mg). Subfraction ZK-14 + Fe-25–0-50–2 was purified on an Agilent 1100 chromatograph using an Agilent 1100 refractometer on a Hydro-RP column eluting with CH_3_CN − H_2_O (70:30) at 2.5 ml/min to yield subfraction ZK-14 + Fe-25–0-50–2-3 (1.5 mg) which was purified using HyperClone column eluting with CH_3_CN − H_2_O (50:50) at 0.8 ml/min to yield **3** (0.5 mg).

Subfraction ZK-14 + Fe-25–1 (224 mg) was purified via preparative HPLC on a Shimadzu LC-20 chromatograph using a Shimadzu RID-20A refractometer on a YMC ODS-AM column eluting with CH_3_CN – H_2_O (80:20) at 2.0 ml/min to subfraction ZK-14 + Fe-25–1 (130 mg) and this subfraction was purified on an Agilent 1100 chromatograph using an Agilent 1100 refractometer on a Hydro-RP column eluting with CH_3_CN − H_2_O (70:30) 2.5 ml/min to yield compound **2** (5.1 mg) and compound **1** (21.7 mg).

### Spectral data of isolated compounds

Helvamide B (**1**): colorless crystal needles (MeOH); mp 144 − 145 °C; $[\alpha]_{\text{D}}^{20}$ + 68.7 (*c* 0.115 MeOH); UV (MeOH) λ_max_ (log ε) 210 (4.80), 199 (4.83), 205 (4.79) nm; CD (c 0.000217 M, MeOH) λ_max_ (∆ε) 204 (+ 3.50), 216 (+ 12.05), 252 (−1.37), 283 (+ 0.31) nm; ^1^H and ^13^C NMR data, see Table 3, Additional file 1: Figs. S5–S9; HRESIMS [M + Na]^+^ *m/z* 571.2193 (calcd for C_34_H_32_N_2_O_5_Na 571.2203, ∆ + 1.8 ppm), [M − H]^−^ *m/z* 547.2215 (calcd. for C_34_H_31_N_2_O_5_ 547.2238, ∆ + 4.2 ppm) (Additional file 1: Fig. S2).

Helvamide C (**2**): colorless amorphous; $[\alpha]_{\text{D}}^{20}$ + 67.6 (*c* 0.327 MeOH); UV (MeOH) λ_max_ (log ε) 198 (4.78), 204 (4.75), 209 (4.75) nm; CD (c 0.000175 M, MeOH) λ_max_ (∆ε) 201 (+ 23.83), 227 (-2.92), 246 (-2.07) nm; ^1^H and ^13^C NMR data, see Table 3, Additional file 1: Figs. S15–S17; HRESIMS [M + Na]^+^ *m/z* 537.2358 (calcd for C_31_H_34_N_2_O_5_Na 537.2360, ∆ + 0.4 ppm), [M − H]^−^ *m/z* 513.2405 (calcd. for C_31_H_34_N_2_O_5_ 513.2395, ∆ − 1.9 ppm) (Additional file 1: Fig. S10).

Saroclazin A (**3**): white powder; $[\alpha]_{\text{D}}^{20}$ − 4.5 (*c* 0.044 MeOH); ^1^H and ^13^C NMR data, see Additional file 1: Table S2, Additional file 1: Figs. S19, S20; HRESIMS [M + Na]^+^ *m/z* 417.1278 (calcd for C_19_H_26_N_2_O_3_S_2_Na 417.1278, ∆ −0.2 ppm), [M − H]^−^ *m/z* 393.1313 (calcd for C_19_H_25_N_2_O_3_S_2_ 393.1312, ∆ − 0.3 ppm) (Additional file 1: Fig. S18).

(4*S*,5*R*,7*S*)-4,11-Dihydroxy-guaia-1(2),9(10)-dien (**4**): colorless oil; $[\alpha]_{\text{D}}^{20}$ + 4.4 (*c* 0.023 MeOH); ^1^H and ^13^C NMR data, see Additional file 1: Table S3, Figs. S22–S24; HRESIMS [M + Na]^+^ *m/z* 259.1669 (calcd. for C_15_H_24_O_2_Na 259.1669, ∆ 0.0 ppm), [M − H]^−^ *m/z* 235.1701 (calcd for C_15_H_23_O_2_ 235.1704, ∆ + 1.3 ppm) (Additional file 1: Fig. S21).

### X-ray crystallographyc data of 1

X-ray analysis which was carried out for a single light-yellow crystal obtained by recrystallization from MeOH–H_2_O (70:30). Experimental intensity data for **1** were collected at T = 100(2)K on a BRUKER Kappa APEX2 diffractometer with graphite monochromated Mo Kα radiation (λ = 0.71073 Å). Intensity data were corrected for absorption using the multi-scan method. The structure was solved using direct methods and refined by least-squares calculation in anisotropic approximation for non-hydrogen atoms. Hydrogen atoms were placed in geometrically idealized positions and refined in the riding-model approximation. Data collection, reduction, and refinement of the lattice parameters were performed using the Apex2 software package (Bruker. APEX 2 V.7, Bruker AXS Inc., Madison, Wisconsin, U.S.A. (2010)). All calculations were performed with SHELXL/PC program [62, 63]. Main crystallographic data and details of refinement of the crystal structure of **1** are shown in Additional file 1: Tables S4-S8, Figs. S25.

The crystallographic data (accession numbers CCDC 2326660) can be obtained free of charge from the Cambridge Crystallographic Data Center via http://www.ccdc.cam.ac. uk/data_request/cif (or from the Cambridge Crystallographic Data Centre, 12 Union Road, Cambridge, UK; fax: + 44 1223 336 033 or email: deposit@ccdc.cam.uk).

C_34_H_32_N_2_O_5_, M = 548, Crystal size: 0.48 × 0.13 × 0.11 mm3, monoclinic, space group C2, a = 46.095(4) Å, b = 6.1121(6) Å, c = 20.2447(19) Å, β = 99.780(5)°, V = 5620.8(9) Å3, Z = 8, Dcalc. = 1.297 g/cm3, μ = 0.087 mm-1. F000 = 2320, θmax = 27.242°, 44,378 reflections collected, 12,335 unique (R(int) = 0.0374). Final GooF = 1.029; for I > 2 σ (I) R1 = 0.0424, wR2 = 0.0957; for all data R1 = 0.0597, wR2 = 0.1050, |Δρ|max = 0.214 e/Å3.

### Quantum-chemical modeling

All quantum-chemical calculations were performed using the B3LYP exchange–correlation functional, the polarization continuum model (PCM) and 6-311G(d) basis set implemented in the Gaussian 16 package of programs [20]. The statistical weights (gim) of the individual conformations were calculated according to equation:

1 $${g}_{im}={e}^{\frac{-\Delta {G}_{im}}{RT}}/\sum_{i}{e}^{\frac{-\Delta {G}_{im}}{RT}}$$

where ΔGim = Gi – Gm are the relative Gibbs free energies and index “m” denotes the most stable conformation.

The ECD spectra were calculated using time-dependent density functional theory (TDDFT), B3LYP functional, PCM model, and 6-311G(d) basis set for conformations, where the relative Gibbs free energies satisfied the relation ΔGim ≤ 4 kcal/mol. To describe well the short-wave region of ECD spectra 95 electronic transitions were calculated for each conformation of 1. The Gauss-type functions were used to simulate the individual bands in the theoretical spectra. The bandwidths ζ = 0.16 eV and the UV shifts Δλ =  + 1 nm were used for best correspondence between experimental and calculated spectra for **1**.

The scaled theoretical and experimental ECD spectra were obtained according to equation:

2 $$\Delta {\varepsilon }_{sc}\left(\lambda \right)=\Delta \varepsilon \left(\lambda \right)/\left|\Delta \varepsilon \left({\lambda }_{peak}\right)\right|$$

where the denominator |Δε (λpeak)| is the modulo of the peak value for the positive characteristic band at λ ≈ 216 nm in the corresponding ECD spectrum.

### Bioassays

#### Cell culture

The human prostate cancer cells PC-3 CRL-1435 and the human embryonic kidney cells HEK-293 CRL-1573™ were purchased from ATCC (American Type Culture Collection, Manassas, VA, USA). PC-3 and HEK-293 cells were cultured in DMEM medium containing 10% fetal bovine serum (Biolot, St. Petersburg, Russia) and 1% penicillin/streptomycin (Biolot, St. Petersburg, Russia) at 37 °C in a humidified atmosphere with 5% (*v/v*) CO_2_. The cells were incubated in cultural flasks until sub-confluent (~ 80%).

#### Cell viability assay

The PC-3 cells (5 × 10^3^ cells/well) and HEK293 cells (7 × 10^3^ cells/well) were seeded in a 96-well plate and incubated overnight. Then, the extracts at a concentration of 100 μg/mL or compounds **1**–**4** at concentrations of 100 µM were added and the cells were further incubated for an additional 24 h. After that, cell viability was determined by MTT (3-(4,5-dimethylthiazol-2-yl)-2,5-diphenyltetrazolium bromide) method based on the manufacturer’s instructions (M5655-500MG, Sigma-Aldrich, St. Louis, MO, USA). The absorbance of the converted formazan was measured using a Multiskan FC microplate photometer (Thermo Scientific, Waltham, MA, USA) at λ = 570 nm. The results were presented as percentages of control data. All experiments were carried out twice in triplicate.

#### Antimicrobial assay

Antimicrobial activity was determined against yeast-like fungi *Candida albicans* KMM 455 from the Collection of Marine Microorganisms PIBOC FEBRAS in liquid nutrient media. Test culture of *C. albicans* was cultured in a Petri dish at 37 °C for 24 h on solid medium Mueller Hinton broth with agar—16.0 g/L.

The assays were performed in 96-well microplates in appropriate Mueller Hinton broth. Each well contained 90 µL of yeast-like fungi *C. albicans* suspension (10^9^ CFU/mL). Then, was added 10 µL of the extracts diluted at 10% DMSO at final concentrations of 100 μg/mL or compounds **1**–**4**. Culture plates were incubated overnight at 37 °C, and the OD_620_ was measured using a Multiskan Spectrum spectrophotometer (Thermo Labsystems Inc., Beverly, MA, USA). All experiments were carried out twice in triplicate. The antimicrobial activity of the extracts was evaluated in comparison with the negative control by the change in optical density and expressed as % inhibition of bacterial growth. Nitrofungin was used as a positive control in a concentration of 1 mg/mL; 1% DMSO solution in dH_2_O was used as a negative control.

#### Radical scavenging assay

DPPH radical scavenging activity of compounds or fungal extracts or compounds **1**–**4** were tested as described [64] with minor modifications. The compounds or fungal extracts were dissolved in DMSO, and the solutions the compounds, fungal extracts, or quercetin (Sigma-Aldrich, Steinheim, Germany) as a positive control (120 µL) were dispensed into wells of a 96-well microplate. In all of them, 30 µL of the DPPH (Sigma-Aldrich, Steinheim, Germany) solution in MeOH (0.75 mM) was added to each well. The concentrations of compounds and quercetin in mixture were 0.1–100.0 µM. The concentrations of fungal extracts in mixture were 0.1–100.0 µg. The plates were incubated in the dark at room temperature for 30 min, and then the absorbance was measured at 517 nm with a Multiskan FC microplate photometer (Thermo Scientific, Waltham, MA, USA). The negative control contained no test compound. The results are presented as percentages of the negative control (DMSO) data.

#### Statistical data evaluation

All data were obtained twice in three independent replicates and calculated values were expressed as a mean ± standard error mean (SEM). Student’s t-test was performed using SigmaPlot 14.0 (Systat Software Inc., San Jose, CA, USA) to determine statistical significance. The differences were considered statistically significant at p < 0.05.

## Conclusions

In summary, the addition of Fe(NO_3_)_3_·9H_2_O to the culture media of the fungus *Penicillium velutinum* caused a significant increase in the production of nitrogen-containing secondary metabolites. Using OSMAC strategy led to the discovery of two new 2,5-dibenzylpiperazine derivatives, helvamides B (**1**) and **C** (**2**), together with known saroclazine A (**3**) and (4S,5R,7S)-4,11-dihydroxy-guaia-1(2),9(10)-dien (**4**). The absolute configuration of helvamide B (**1**) was determined using a combination of X-ray analysis and time-dependent density functional theory (TD-DFT) calculations of the ECD spectra. Previously, compounds **3** and **4** were not reported as metabolites of the fungus *P. velutinum*. Saroclazine A (**3**), helvamide B (**1**), and especially helvamide C (**2**) can inhibit the growth of *Candida albicans*. These new data open interesting possibilities for further research on these compounds as antifungal agents.

### Supplementary Information

**Additional file 1.** Additional tables and figures.

## Data Availability

All data generated or analyzed during this study are included in this published article and itsinformation files.

## Appendix

See Table 5

**Table 5:** The identified metabolites of the *P.velutinum* ZK-14

№	Name	Structure	RT	Exact mass (measured)	Exact mass (calcd)	∆, ppm	MQScore (GNPS)	Refs.
I	–*	C_14_H_10_O_6_	3.3	275.0546 ([M + H]^+^)	275.0550	− 1.45	–	–
II	–*	C_13_H_10_O_5_	4.8	247.0594 ([M + H]^+^)	247.0601	− 2.83	–	–
III	–*	C_15_H_22_O_3_	6.3	251.1622 ([M + H]^+^)	251.1642	− 7.96	–	–
IV	–*	C_15_H_24_O_2_	8.8	237.1837 ([M + H]^+^)	237.1849	− 5.06	–	–
V	Fulvic acid*		9.4	291.0480 ([M−H_2_O + H]^+^)	291.0499	− 6.53	0.87	[29, 30]
VI	–*	C_15_H_22_	10.2	203.1787 ([M + H]^+^)	203.1794	− 3.45	–	–
VII	Helvamide B**		13.3	571.2193 ([M + Na]^+^)	571.2203	1.8	–	–
VIII	–**	C_27_H_44_NO_6_	13.4	500.2982 ([M + Na]^+^)	500.2983	0.2	–	–
IX	–**	C_28_H_37_N_2_O_5_	13.9	503.2519 ([M + Na]^+^)	503.2516	− 0.6	–	–
	Helvamide C**		13.9	537.2358 ([M + Na]^+^)	537.236	0.4	–	–
X	–**	C_31_H_35_N_2_O_5_	14.3	537.2363 ([M + Na]^+^)	537.236	− 0.6	–	–
XI	–*	C_25_H_41_N_7_O_2_	18.4	472.3396 ([M + H]^+^)	472.3395	0.21	–	–
XII	–*	C_27_H_48_O_7_	19.4	484.3387 ([M]^+^)	484.3395	− 1.65	–	–
XIII	Fallacinol*		7.0	301.0699 ([M + H]^+^)	301.0707	− 2.66	0.92	[32]
XIV	(4*S*,5*R*,7*S*)-4,11-dihydroxy-guaia-1(2),9(10)-dien**		8.2	259.1666 ([M + Na]^+^)	259.1669	− 1.2	–	[45, 46]
XV	Sulochrin*		8.9	333.0967 ([M + H]^+^)	333.0969	− 0.60	0.95	[35, 36]
	Methylsulochrin*		8.9	209.0437 ([M−C_8_H_10_O_2_]^+^)	209.0445	− 3.8	0.83	[34, 35]
XVI	Saroclazin A*		12.1	417.1278 ([M + Na]^+^)	417.1277	− 0.2	–	[44]

*Compound was identified based on an exact *m/z* value

** Compounds were identified by comparison of MS/MS data and retention times with those obtained for authentic standards isolated from this fungal strain
