# Supplementary material for: New piperazine derivatives helvamides B–C from the marine-derived fungus Penicillium velutinum ZK-14 uncovered by OSMAC (One Strain Many Compounds) strategy
Source: Nat Prod Bioprospect. 2024 May 21;14(1):32. doi: 10.1007/s13659-024-00449-9 (PMC11106049; doi:10.1007/s13659-024-00449-9)
Supplement: Supplementary file 1 — Additional file 1. Additional tables and figures. [file 13659_2024_449_MOESM1_ESM.pdf]

Supporting Information

Regular Article

New Piperazine Derivatives Helvamides B–C from the Marine-Derived Fungus *Penicillium velutinum* ZK-14 Uncovered by OSMAC (One Strain Many Compounds) Strategy

Gleb V. Borkunov [a,b†], Elena V. Leshchenko [a,b,†,*] Dmitrii V. Berdyshev [a], Roman S. Popov [a], Ekaterina A. Chingizova [a], Nadezhda P. Shlyk [b], Andrey V. Gerasimenko [c], Natalya N. Kirichuk [a], Yuliya V. Khudyakova [a], Viktoria E. Chausova [a], Alexandr S. Antonov [a], Anatoly I. Kalinovsky [a], Artur R. Chingizov [a], Ekaterina A. Yurchenko [a], Marina P. Isaeva [a] and Anton N. Yurchenko[a]

[a] G.B. Elyakov Pacific Institute of Bioorganic Chemistry, Far Eastern Branch of the Russian Academy of Sciences, 159 Prospect 100-letiya Vladivostoka, Vladivostok 690022, Russian Federation;

[b] Far Eastern Federal University, Vladivostok 690922, Russian Federation;

[c] Institute of Chemistry, Far Eastern Branch of the Russian Academy of Sciences, 159 Prospect 100-letiya Vladivostoka, Vladivostok 690022, Russian Federation;

* Correspondence: leshchenko.ev@dvfu.ru, ORCID 0000-0002-9429-5684

† These authors contributed equally to this work.

Table S1. The strains of the species used in multi-locus phylogenetic analysis and GenBank accession numbers. 3

Figure S1. Molecular identification of the fungal strain 4

Figure S2. HRESIMS data for helvamide B (**1**)........ 5

Figure S3. UV data of **1** 7

Figure S4. CD data for **1** 8

Figure S5. ^{1}H NMR spectrum of **1** measured at 300 MHz in acetone-d$_6$........ 9

Figure S6. ^{13}C NMR spectrum of **1** measured at 300 MHz in acetone-d$_6$........ 11

Figure S7. DEPT-135 spectrum of **1** measured at 500 MHz in acetone-d$_6$........ 12

Figure S8. COSY spectrum of **1** measured at 500 MHz in acetone-d$_6$ 13

Figure S9. HMBC spectrum of **1** measured at 500 MHz in acetone-d$_6$ 15

Figure S10. HRESIMS data for helvamide C **(2)**........ 16

Figure S11. Fragmentation of the cationized molecule at *m/z* 537 in CID MS (tandem mass spectrometry) for helvamide C **(2)** 18

Figure S12. Fragmentation of the cationized molecule at *m/z* 537 in CID MS (tandem mass spectrometry) HRESIMS data for helvamide C **(2)** 19

Figure S13. UV data for **2**........ 20

Figure S14. CD data for **2** 21

Figure S15. ^{1}H NMR spectrum of **2** measured at 700 MHz in acetone-d$_6$........ 22

Figure S16. ^{13}C NMR spectrum of **2** measured at 175MHz in acetone-d$_6$........ 23

Figure S17. DEPT-135 spectrum of **2** measured at 175 MHz in acetone-d$_6$........ 24

Table S2. ^{13}C and ^{1}H NMR spectroscopic data for saroclazin A (**3**)........ 25

Figure S18. HRESIMS data for for saroclazin A (**3**) 26

Figure S19. ^{1}H NMR spectrum of **3** measured at 500 MHz in acetone-d$_6$........ 28

Figure S20. ^{13}C NMR spectrum of 3 measured at 500 MHz in acetone-d$_6$........ 29

Table S3. ^{13}C and ^{1}H NMR spectroscopic data for (4S,5R,7S)-4,11-dihydroxy-guaia-1(2),9(10)-dien (**4**)....................................... 30

Figure S21. HRESIMS data for **4** 31

Figure S22. ^{1}H NMR spectrum of **4** measured at 500 MHz in acetone-d_6 33

Figure S23. ^{13}C NMR spectrum of **4** measured at 125 MHz in acetone-d_6 34

Figure S24. DEPT spectrum of **4** in acetone-d_6 35

Figure S24. X-ray data of 1 36

Table S4. Crystal data and structure refinement for Tib2023_04b_1_0m. 39

Table S5. Atomic coordinates (x 10^4) and equivalent isotropic displacement parameters (E^{2}x 10^3) 40

Table S6. Bond lengths [E] and angles [°] for Tib2023_04b_1_0m. 43

Table S7. Anisotropic displacement parameters (E^{2}x 10^3) for Tib2023_04b_1_0m. 54

Figure S26. The large-amplitude motions (LAM), proceeding in *R*,*R*-**1**. 59

Figure S27. The most stable conformations of *R*,*R*-**1**.. 60

Figure S28. The conformational rearrangement, proceeding due to inversion of the piperazine ring. 61

Figure S29. The most stable conformations of *R*,*R*-**2a** and *S*,*S*-**2a,** *R*,*R*-**2b** and *S*,*S*-**2b**. 62

Figure S30. LC-UV chromatograms of extracts from fungus *Penicillium velutinum* ZK-14 cultivated with metal ions. 64

Figure S31. HPLC UV chromatogram of the extract of the fungus *Penicillium velutinum* (ZK-14) cultivated on rise medium..... 65

Figure S32. HPLC UV chromatogram of the extract of the fungus *Penicillium velutinum* (ZK-14) cultivated on rise medium with 100 µm Mg^{2+} salt concentration (**PvMg)**. 66

Figure S33. HPLC UV chromatogram of the extract of the fungus *Penicillium velutinum* (ZK-14) cultivated on rise medium with 100 µm Fe^{3+} salt concentration (**PvFe**). 67

Figure S34. HPLC UV chromatogram of the extract of the fungus *Penicillium velutinum* (ZK-14) cultivated on rise medium with 100 µm Zn^{2+} salt concentration (**PvZn**). 68

Figure S35. HPLC UV chromatogram of the extract of the fungus *Penicillium velutinum* (ZK-14) cultivated on rise medium with 100 µm Ni^{2+} salt concentration (**PvNi**). 69

Figure S36. HPLC MS retention time and MS/MS of compound **1** 70

Figure S37. HPLC MS retention time and MS/MS of compound **2** 71

Figure S38. HPLC MS retention time and MS/MS of (4S,5R,7S)-4,11-dihydroxy-guaia-1(2),9(10)-dien **4**. 72

Figure S39. HPLC MS retention time and MS/MS of peak **VIII.** 73

Figure S40. HPLC MS retention time and MS/MS of peak **IXa**. 74

Figure S41. HPLC MS retention time and MS/MS of peak **X**. 75

Figure S42. The MZmine processing settings 76

Table S1. The strains of the species used in multi-locus phylogenetic analysis and GenBank accession numbers.

Species	Strain Number	GenBank Accession Numbers			
		ITS	*BenA*	*CaM*	*RPB2*
Penicillium aotearoae Visagie et. Seifert	KAS 3088 T	KT887874	KT887835	KT887796	MN969174
Penicillium atrosanguineum B.X. Dong	CBS 380.75 T	JN617706	KJ834435	KP016771	JN406557
Penicillium burgense Quintan. ex Visagie	CBS 325.89 T	KC411736	KJ834437	KP016772	JN406572
Penicillium diabolicalicense Visagie & Seifert	KAS 1726 T	KT887840	KT887801	KT887762	MN969175
Penicillium hemitrachum Visagie & K. Jacobs	CBS 139134 T	FJ231003	JX141048	JX157526	KP064642
Penicillium Penicillium lapidosum Raper & Fennell	CBS 343.48 T	MN431392	KJ834465	FJ530984	JN121500
Penicillium maclennaniae H.Y. Yip	CBS 198.81 T	KC411689	KJ834468	KP016791	KP064648
Penicillium melinii Thom	NRRL 2041 T	AF033449	KJ834471	KP016792	JN406613
Penicillium namyslowskii K.W. Zaleski	NRRL 1070 T	AF033463	JX141067	KP016795	JF417430
Penicillium raciborskii K.W. Zaleski	NRRL 2150 T	AF033447	JX141069	KP016800	JN406607
Penicillium smithii Quintan	CBS 276.83 T	KC411723	KJ834492	KP016806	JN406589
Penicillium terrenum D.B. Scott	CBS 313.67 T	AM992111	KJ834496	KP016808	JN406577
Penicillium velutinum J.F.H. Beyma	NRRL 2069 T	AF033448	JX141170	MT478037	KP064682
Penicillium velutinum	**ZK-14**	**OQ427361**	**OQ466610**	**OR356207**	**OR356208**
Penicillium xanthomelinii Visagie & K. Jacobs	CV 1677 T	JX140921	JX141120	JX157495	KP064683
Talaromyces marneffei (Segretain, Capponi *et* Sureau) Samson, N. Yilmaz, Frisvad *et* Seifert	CBS 388.87 T	JN899344	JX091389	KF741958	KM023283

Figure S1. Molecular identification of the fungal strain

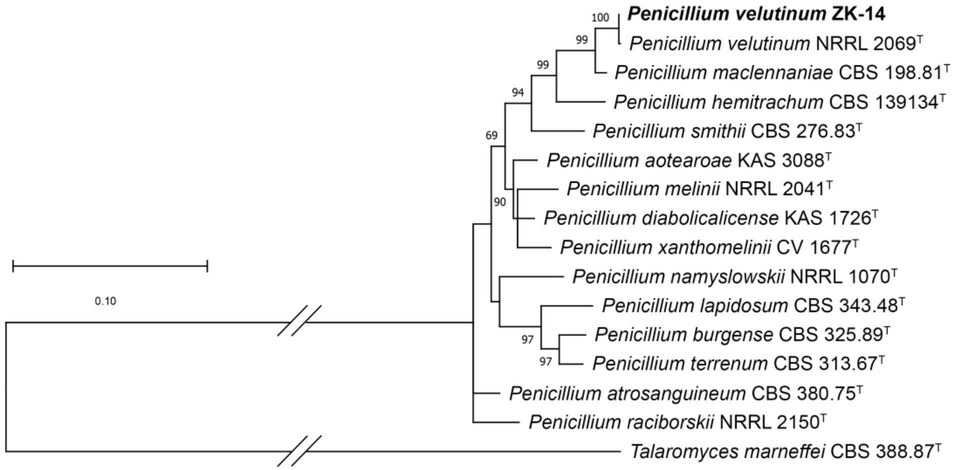

ML tree based on concatenated ITS-*BenA*-*CaM*-*RPB2* gene sequences showing phylogenetic position of the strain ZK-14 among members of genus *Penicillium* section *Exilicaulis*, series *Lapidosa*. Bootstrap values (%) of 1000 replications. Nodes with confidence values greater than 50% are indicated. The scale bars represent 0.10 substitutions per site.

Figure S2. HRESIMS data for helvamide B (**1**)

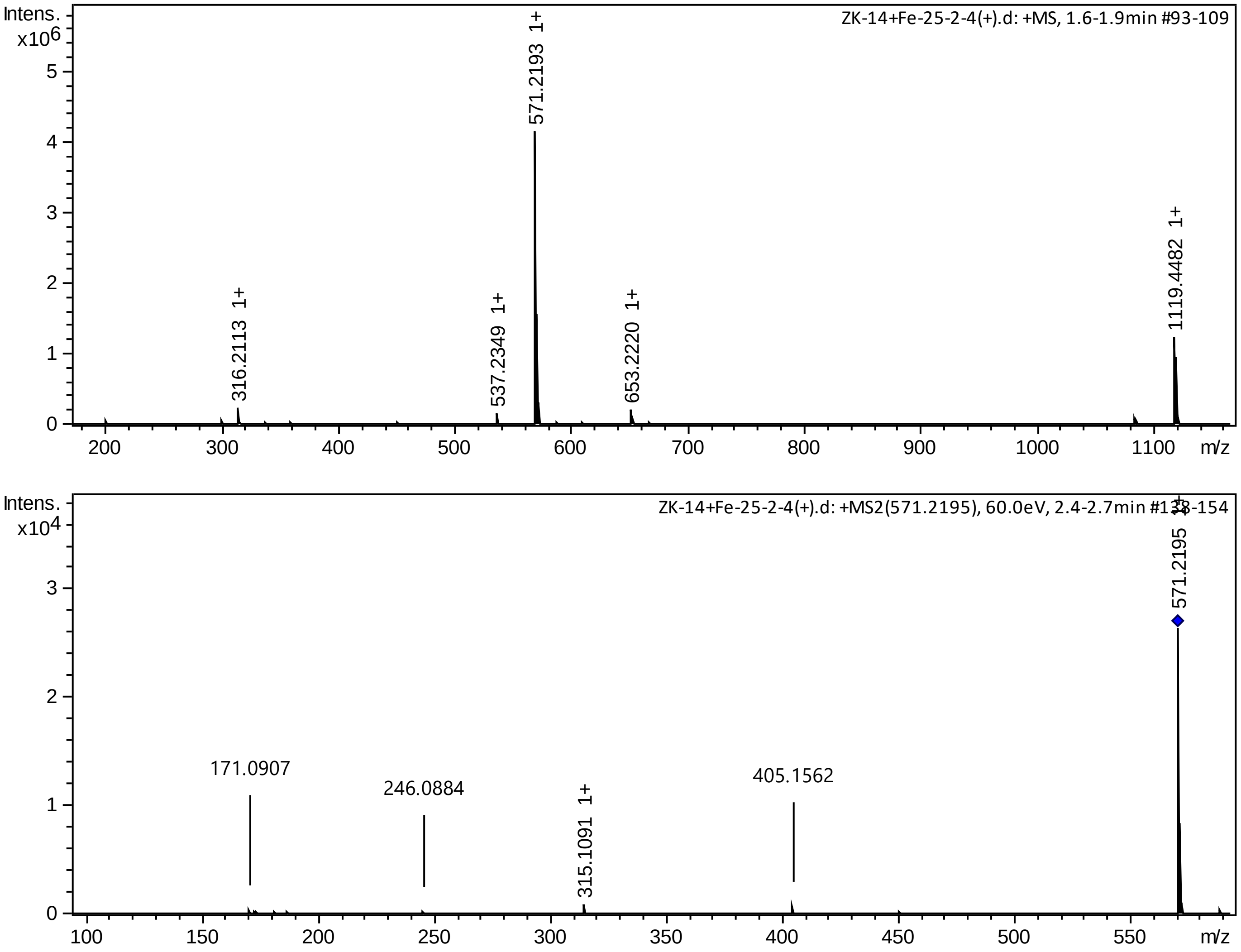

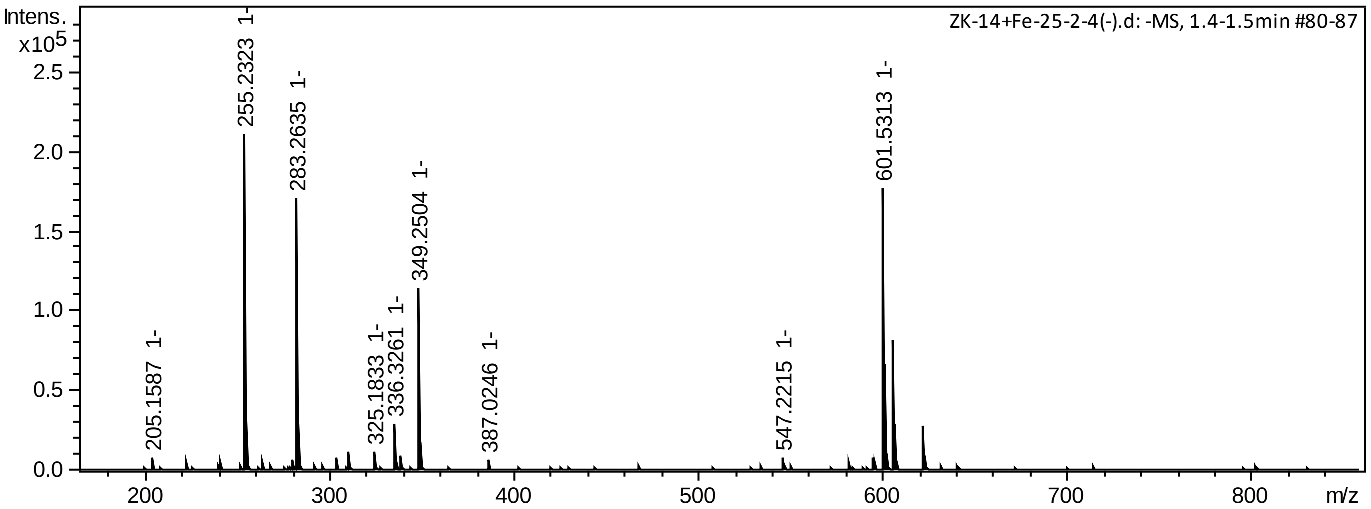
ZK-14+Fe-25-2-4(-).d: -MS, 1.4-1.5min #80-87
Intens. x10^5
205.1587 1-
255.2323 1-
283.2635 1-
325.1833 1-
336.3261 1-
349.2504 1-
387.0246 1-
547.2215 1-
601.5313 1-
m/z

Figure S3. UV data of **1**

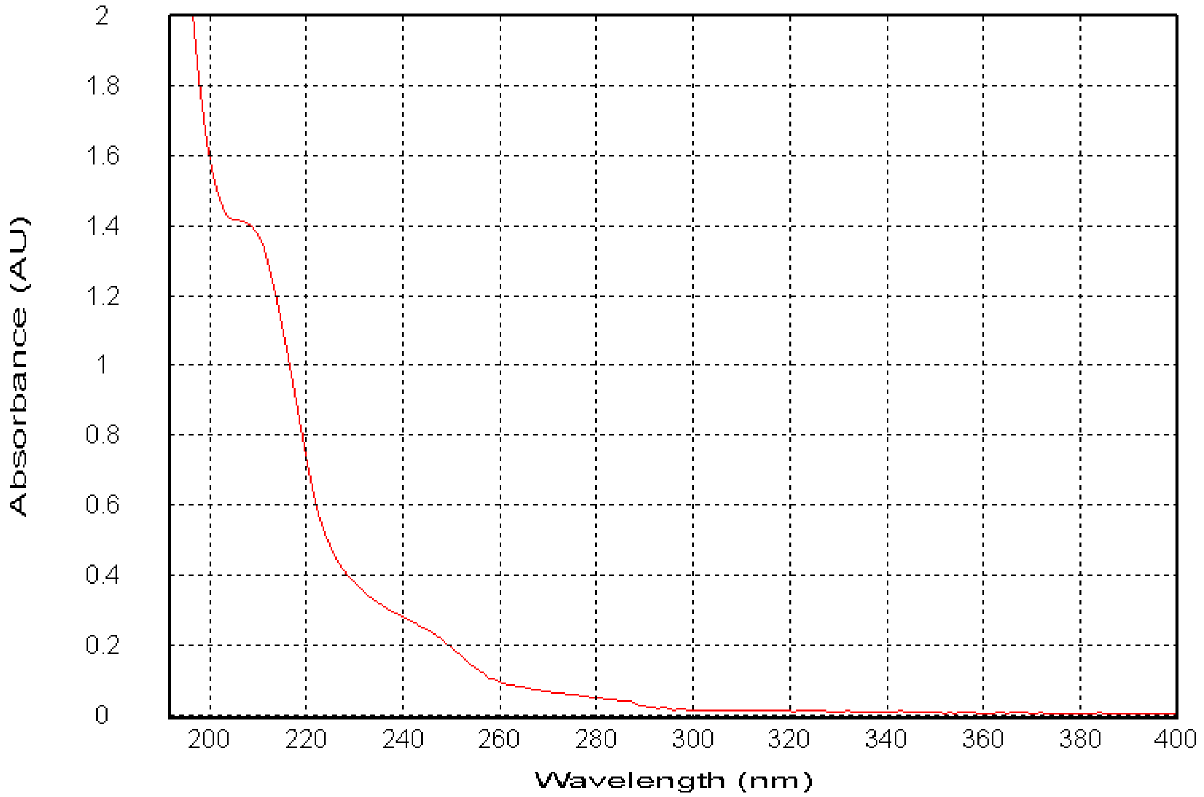

Figure S4. CD data for **1**

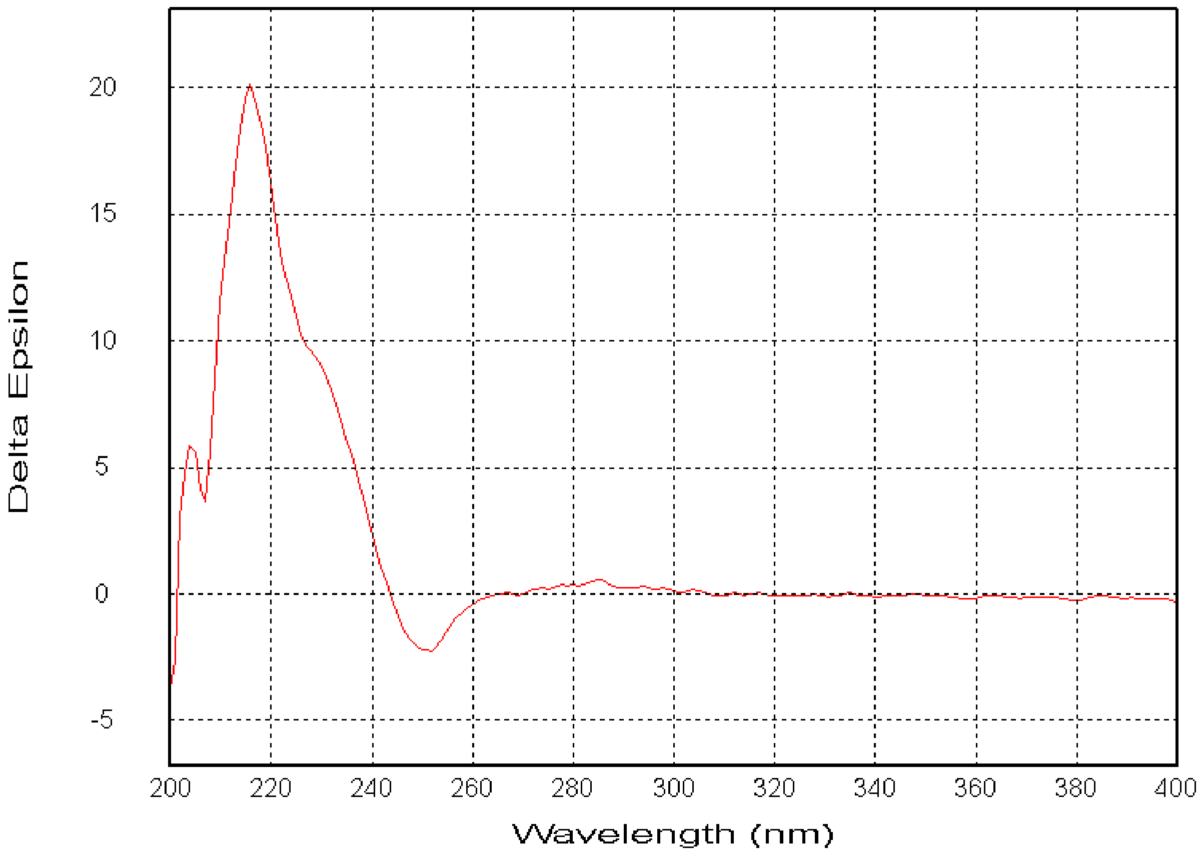

Figure S5. ^{1}H NMR spectrum of **1** measured at 300 MHz in acetone-d_6

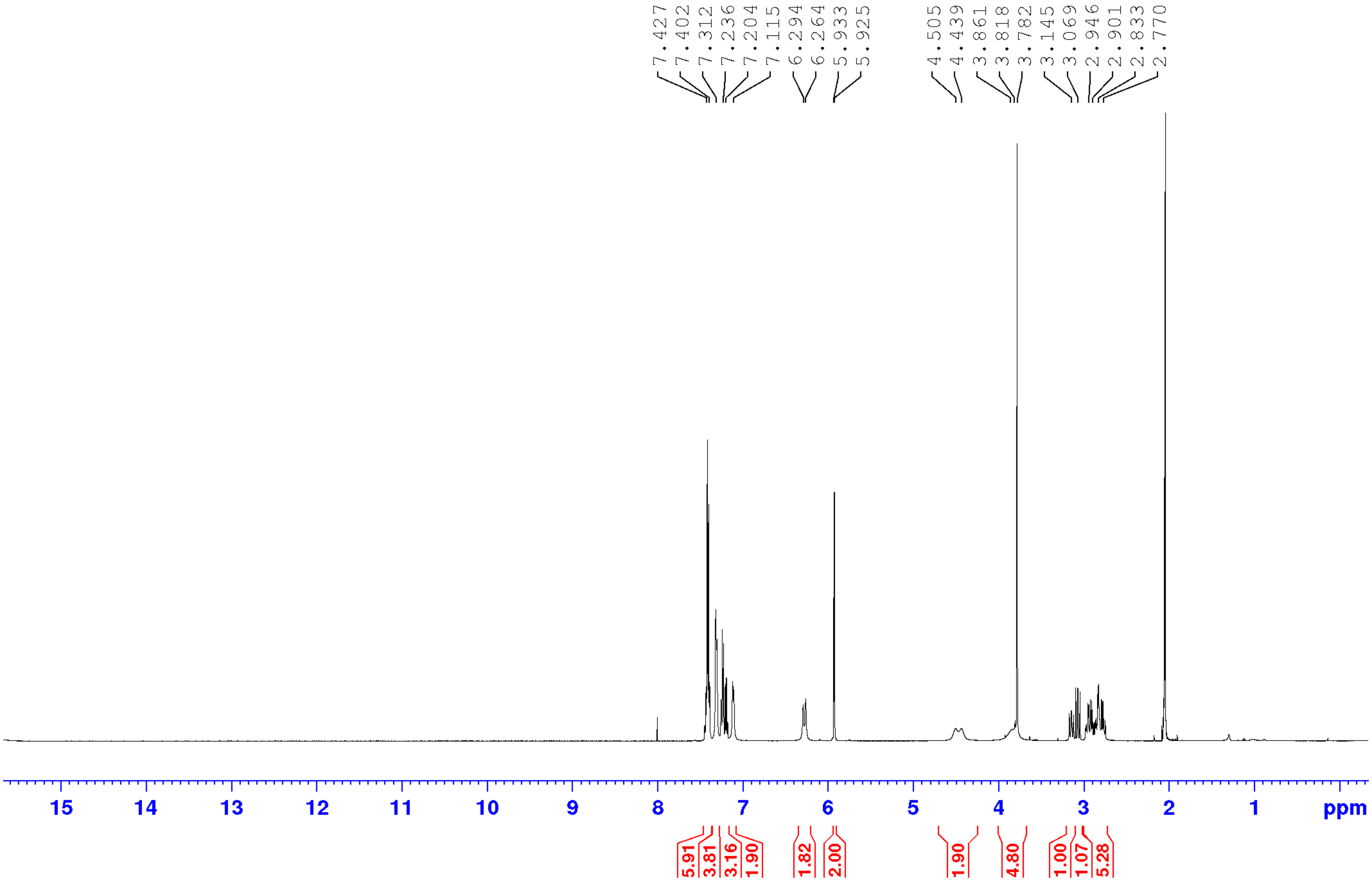

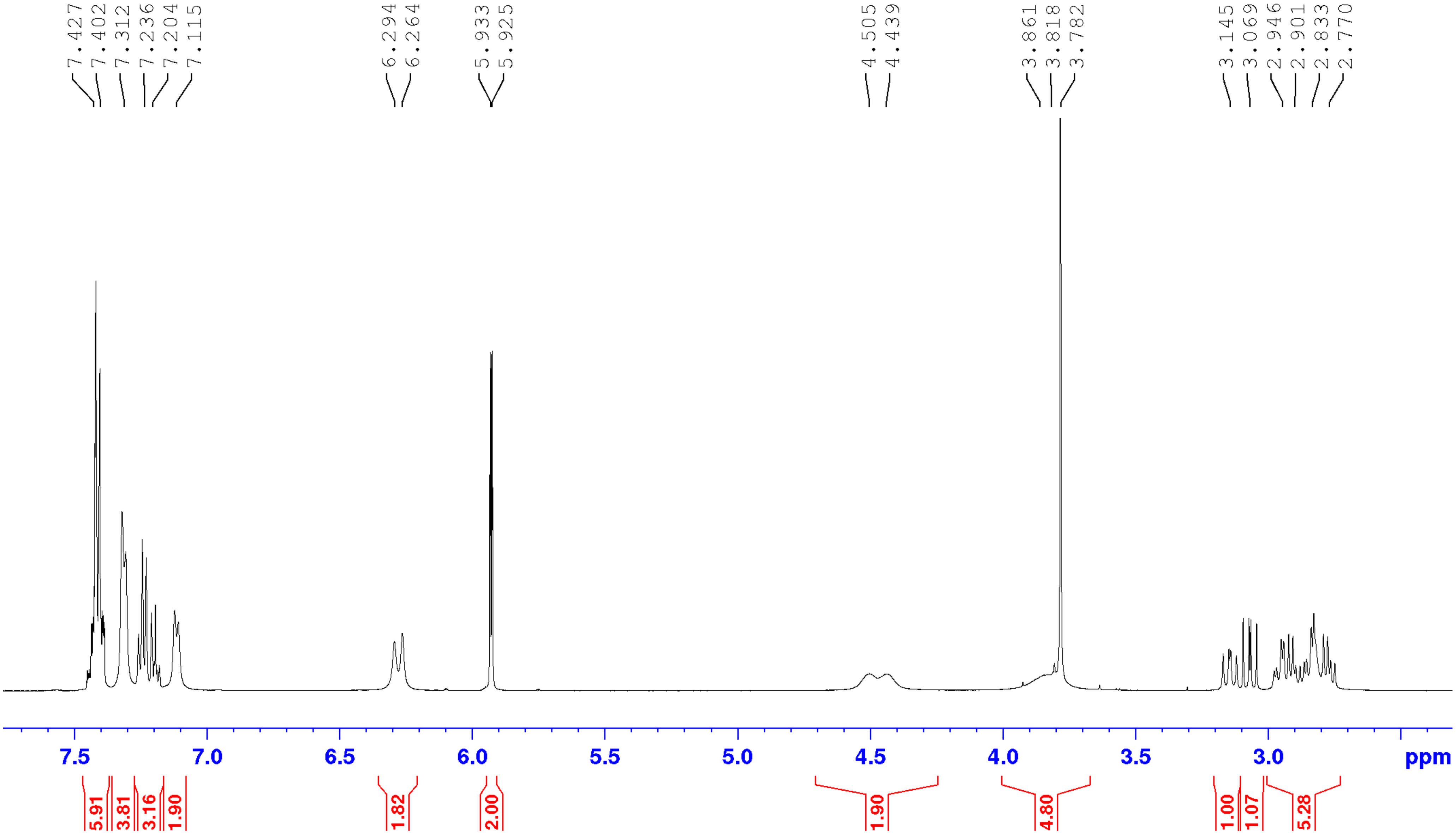
7.427
7.402
7.312
7.236
7.204
7.115
6.294
6.264
5.933
5.925
4.505
4.439
3.861
3.818
3.782
3.145
3.069
2.946
2.901
2.833
2.770
7.5
7.0
6.5
6.0
5.5
5.0
4.5
4.0
3.5
3.0
ppm
5.91
3.81
3.16
1.90
1.82
2.00
1.90
4.80
1.00
1.07
5.28

Figure S6. ^{13}C NMR spectrum of **1** measured at 300 MHz in acetone-d_6

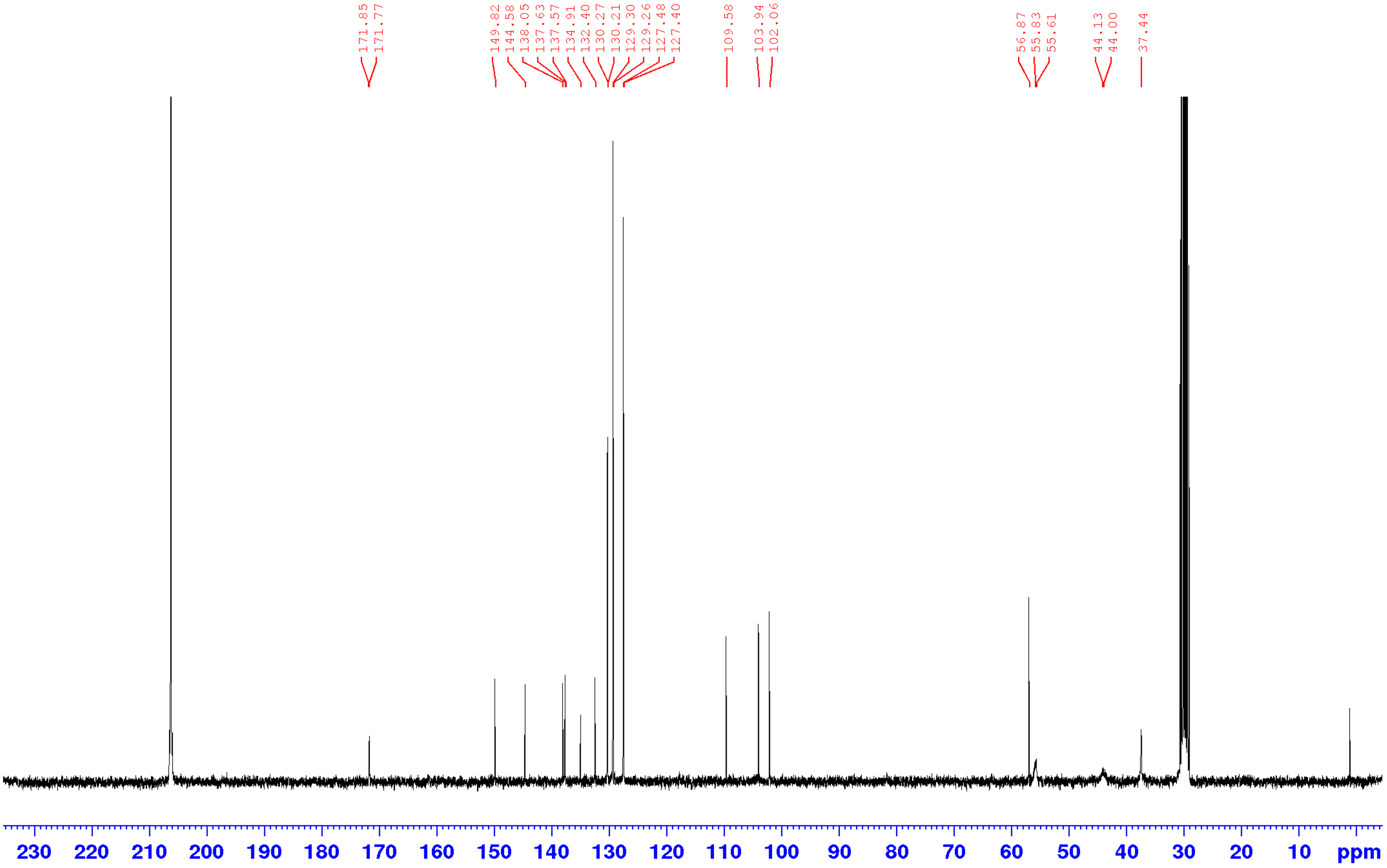

Figure S7. DEPT-135 spectrum of **1** measured at 500 MHz in acetone-d_6

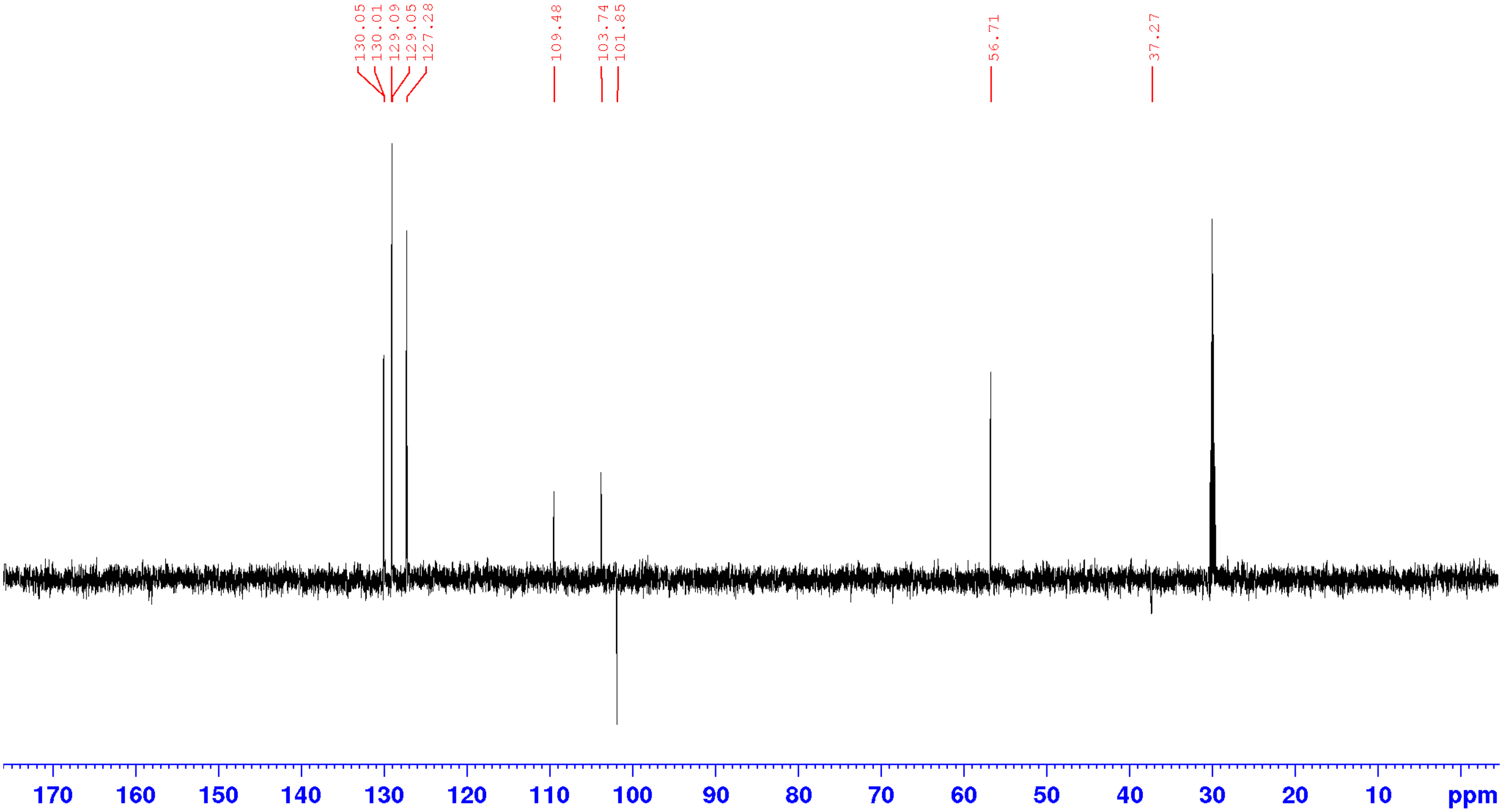

Figure S8. COSY spectrum of **1** measured at 500 MHz in acetone-d_6

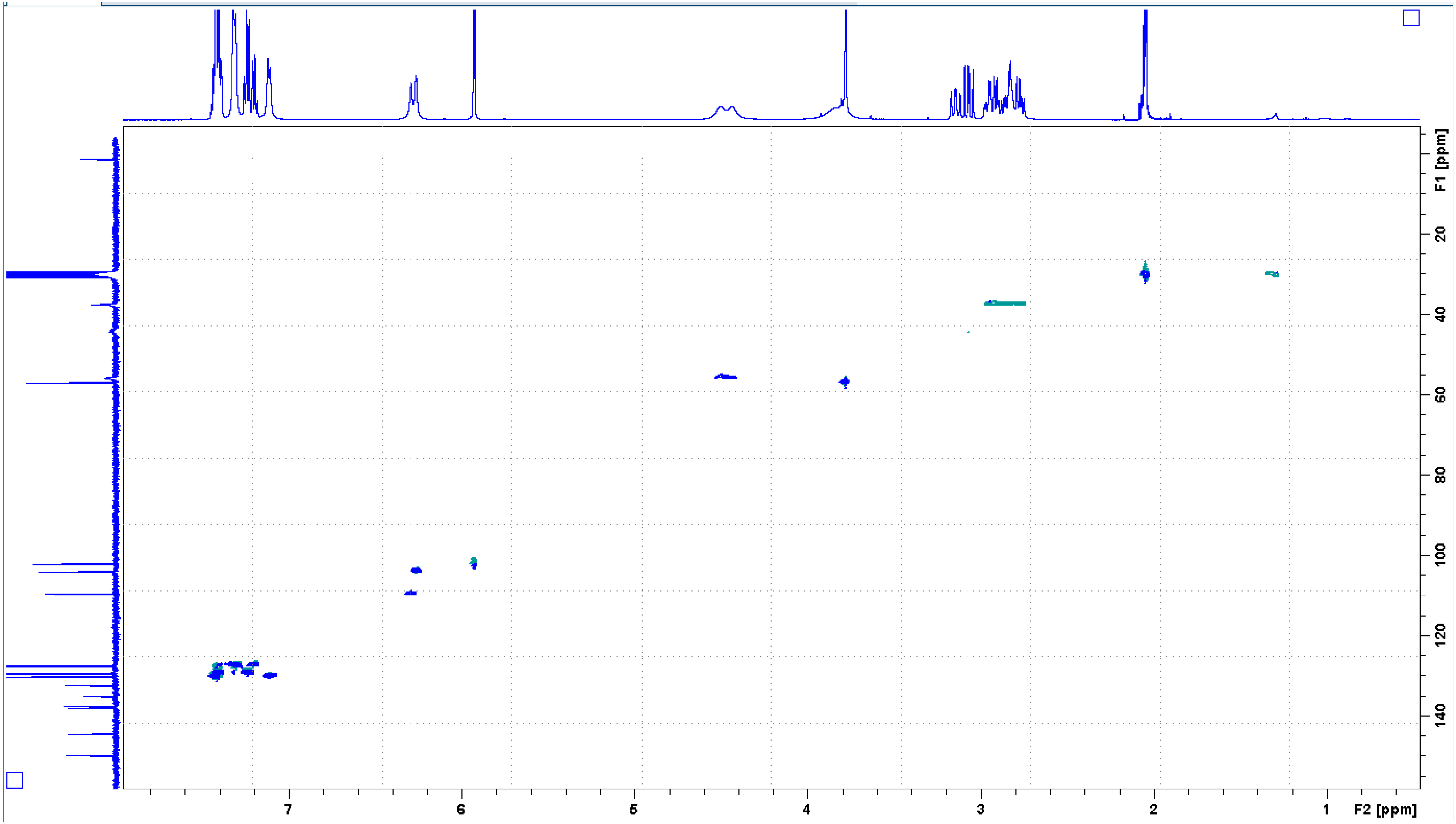

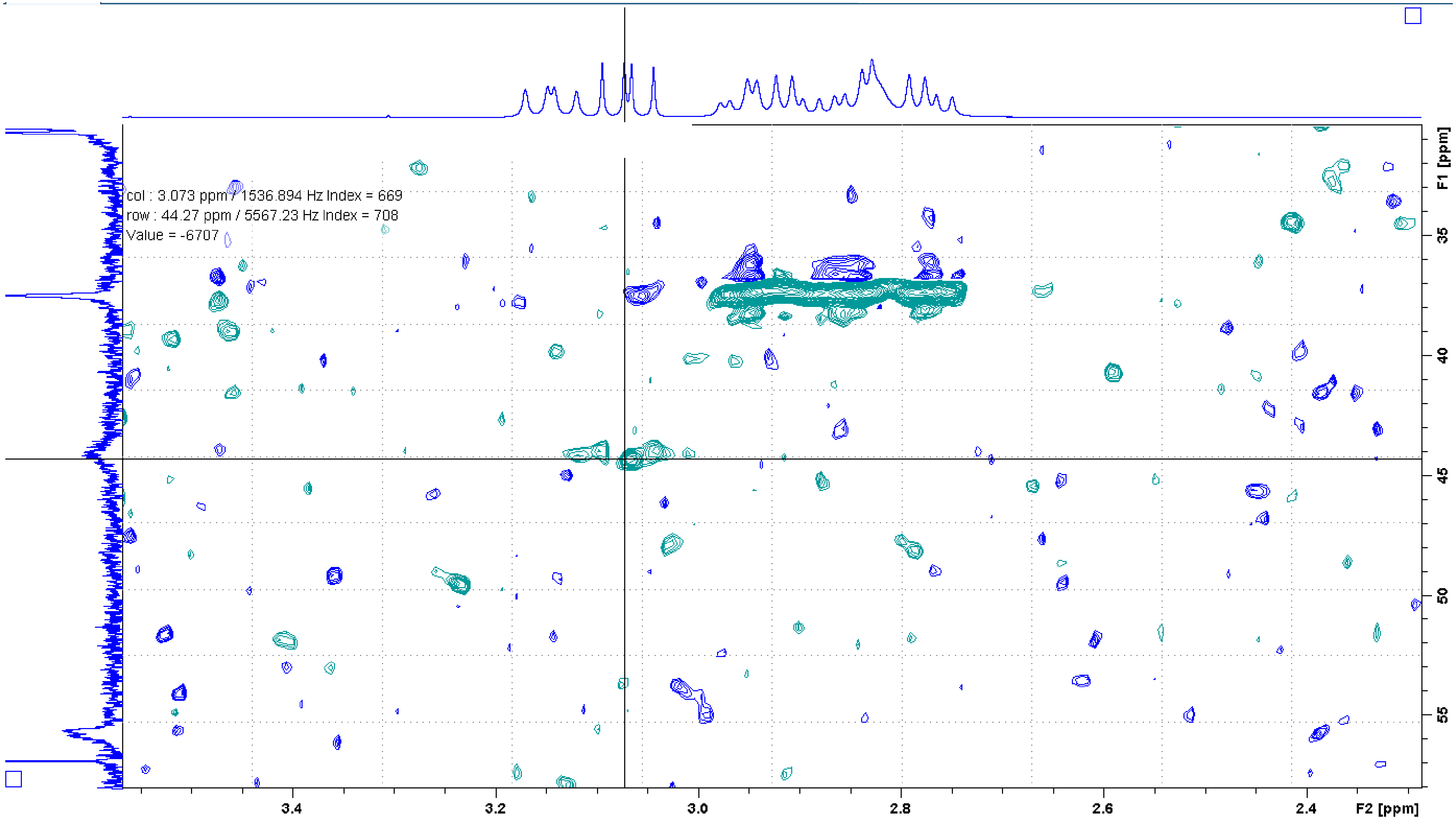

col : 3.073 ppm / 1536.894 Hz Index = 669
row : 44.27 ppm / 5567.23 Hz Index = 708
Value = -6707
F1 [ppm]
35
40
45
50
55
3.4
3.2
3.0
2.8
2.6
2.4
F2 [ppm]

Figure S9. HMBC spectrum of **1** measured at 500 MHz in acetone-d_6

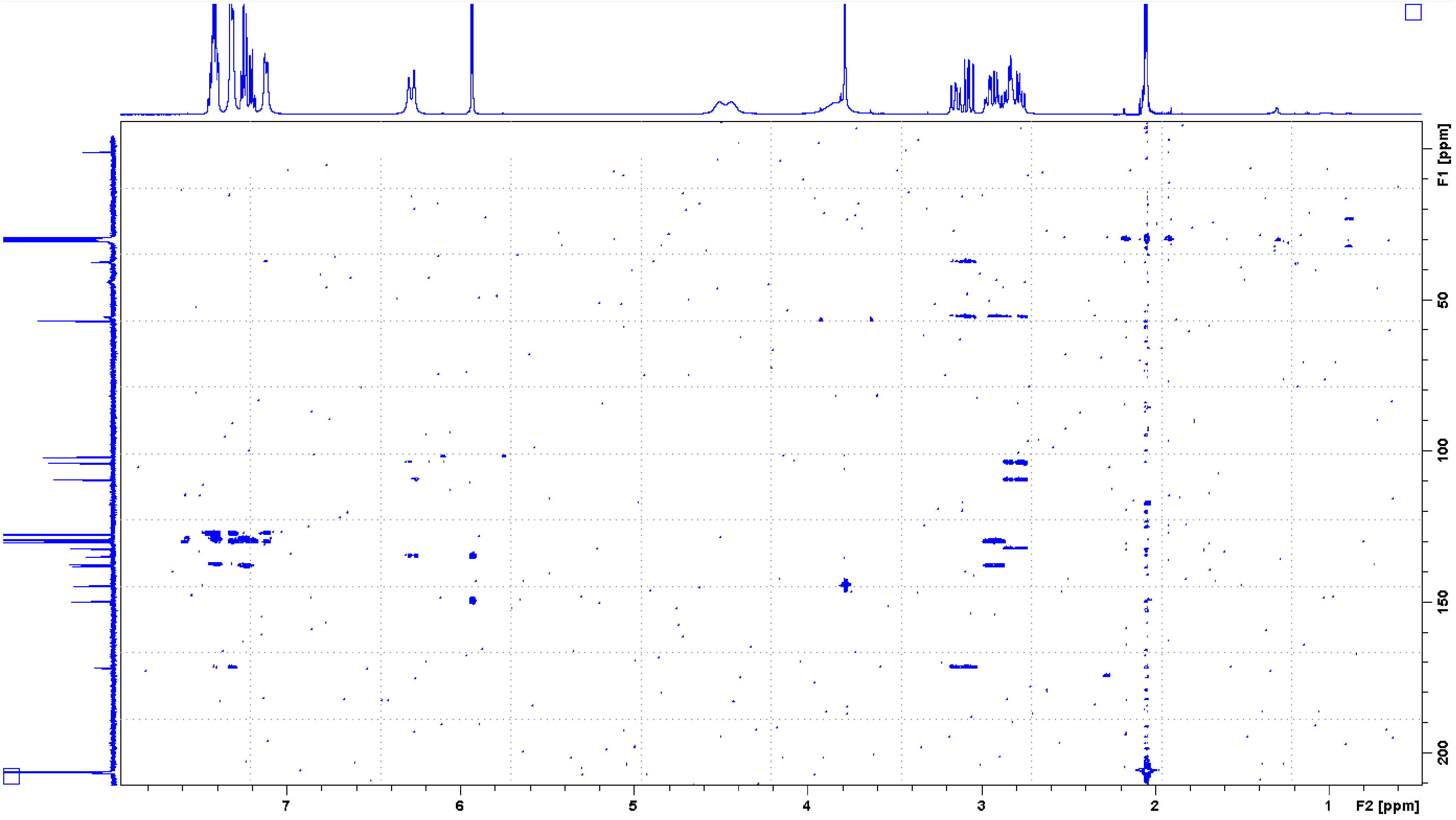

Figure S10. HRESIMS data for helvamide C **(2)**

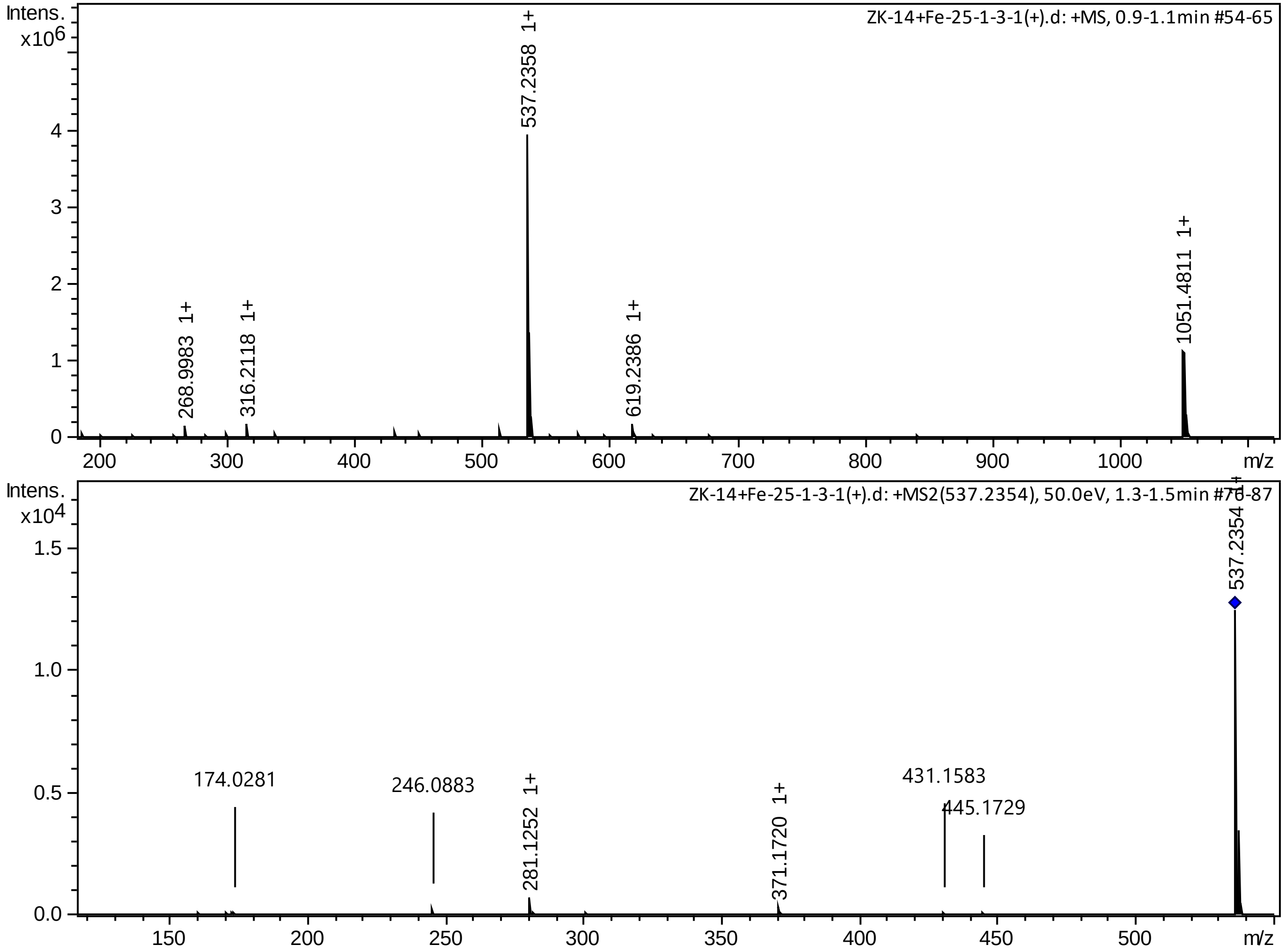

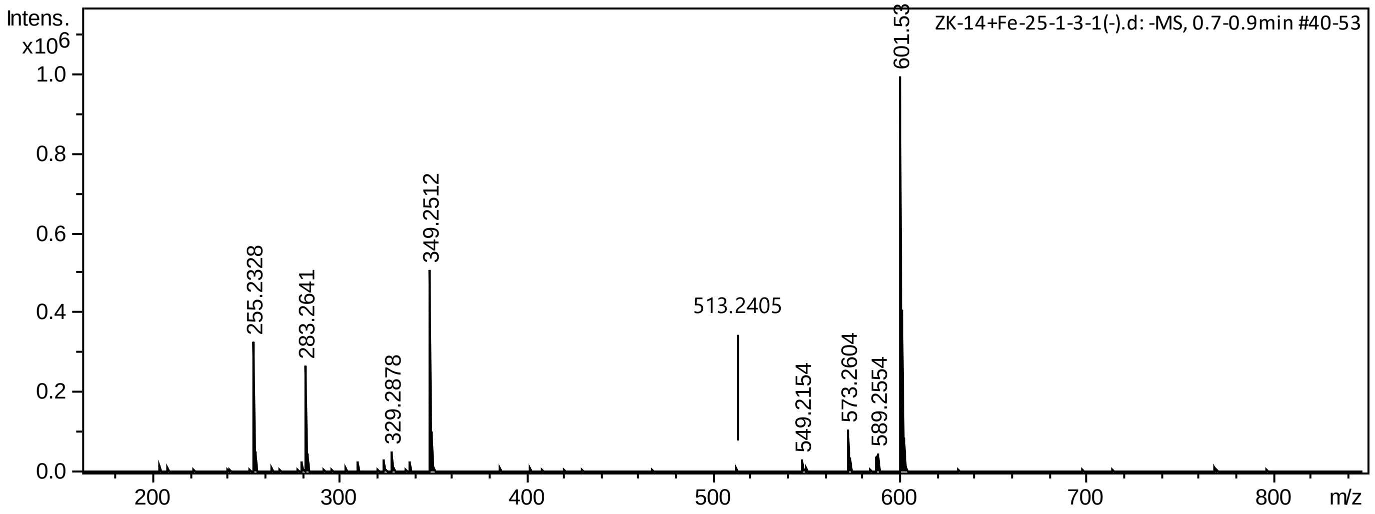

Intens.
x10^6
ZK-14+Fe-25-1-3-1(-).d: -MS, 0.7-0.9min #40-53
601.53
349.2512
255.2328
283.2641
329.2878
513.2405
549.2154
573.2604
589.2554
m/z

Figure S11. Fragmentation of the cationized molecule at *m/z* 537 in CID MS (tandem mass spectrometry) for helvamide C **(2)**

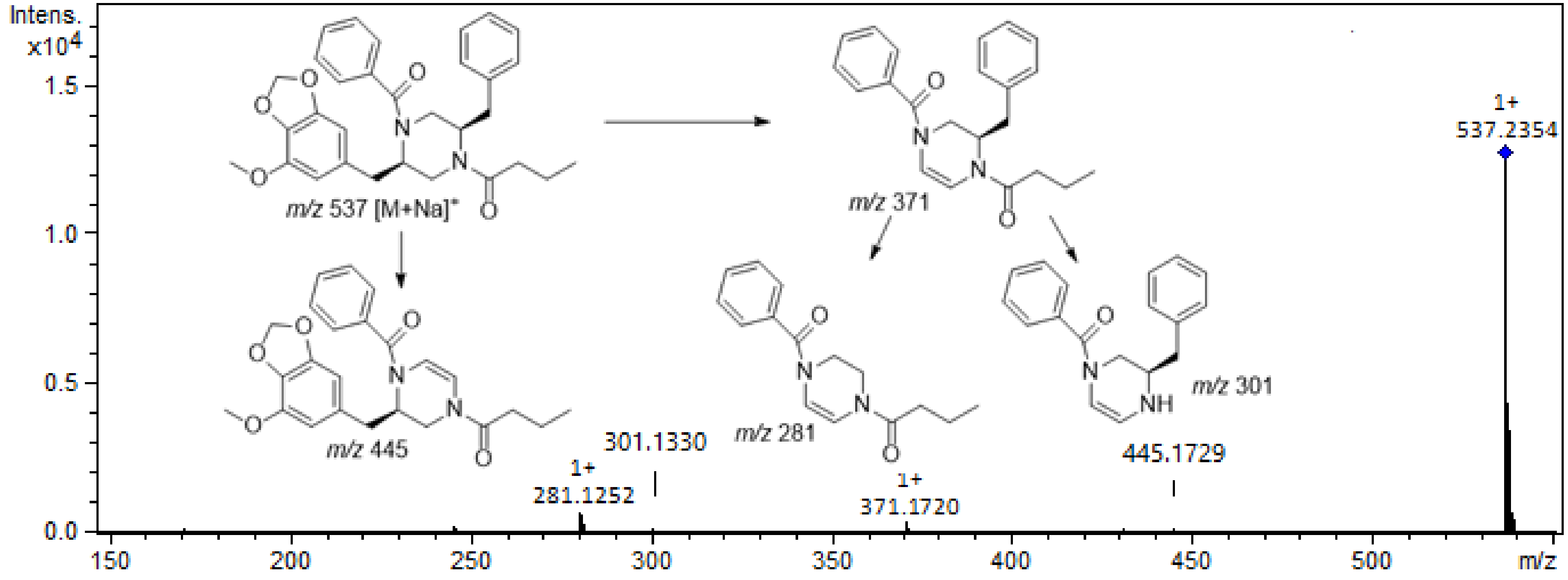

Figure S12. Fragmentation of the cationized molecule at *m/z* 537 in CID MS (tandem mass spectrometry) HRESIMS data for helvamide C **(2)**

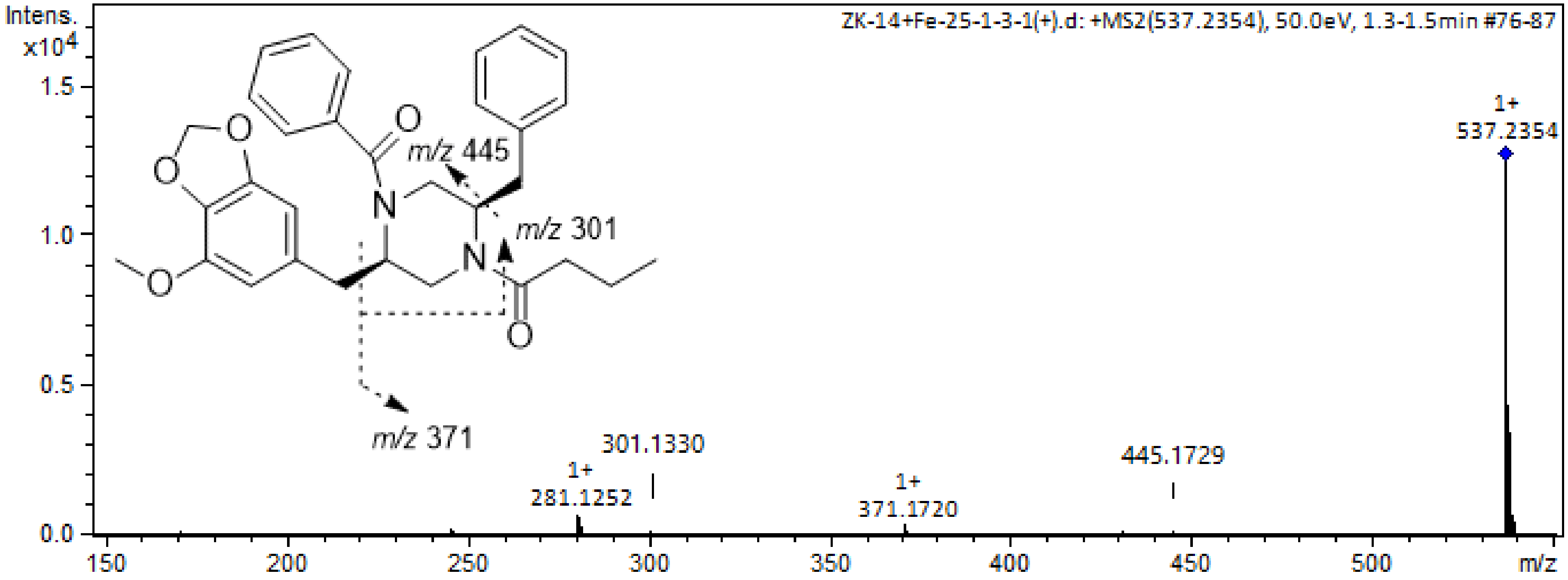

Figure S13. UV data for **2**

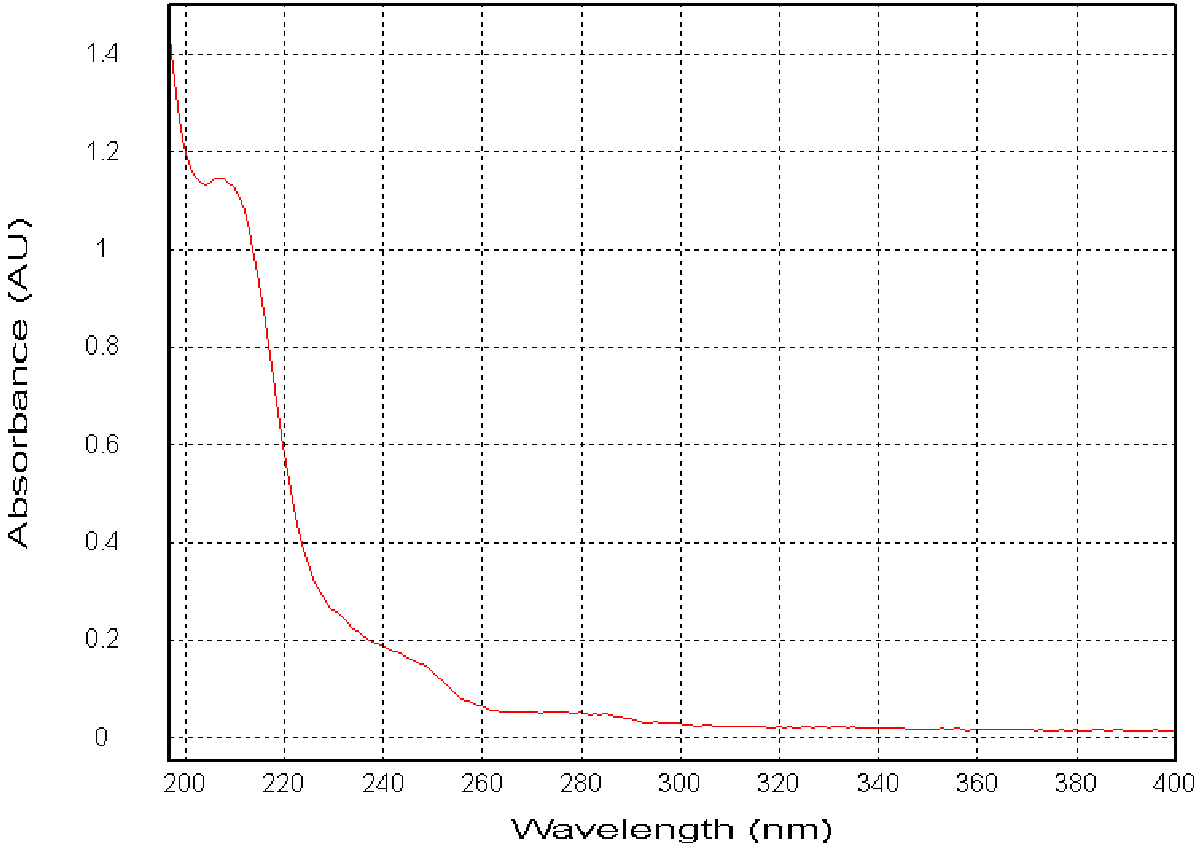

Figure S14. CD data for **2**

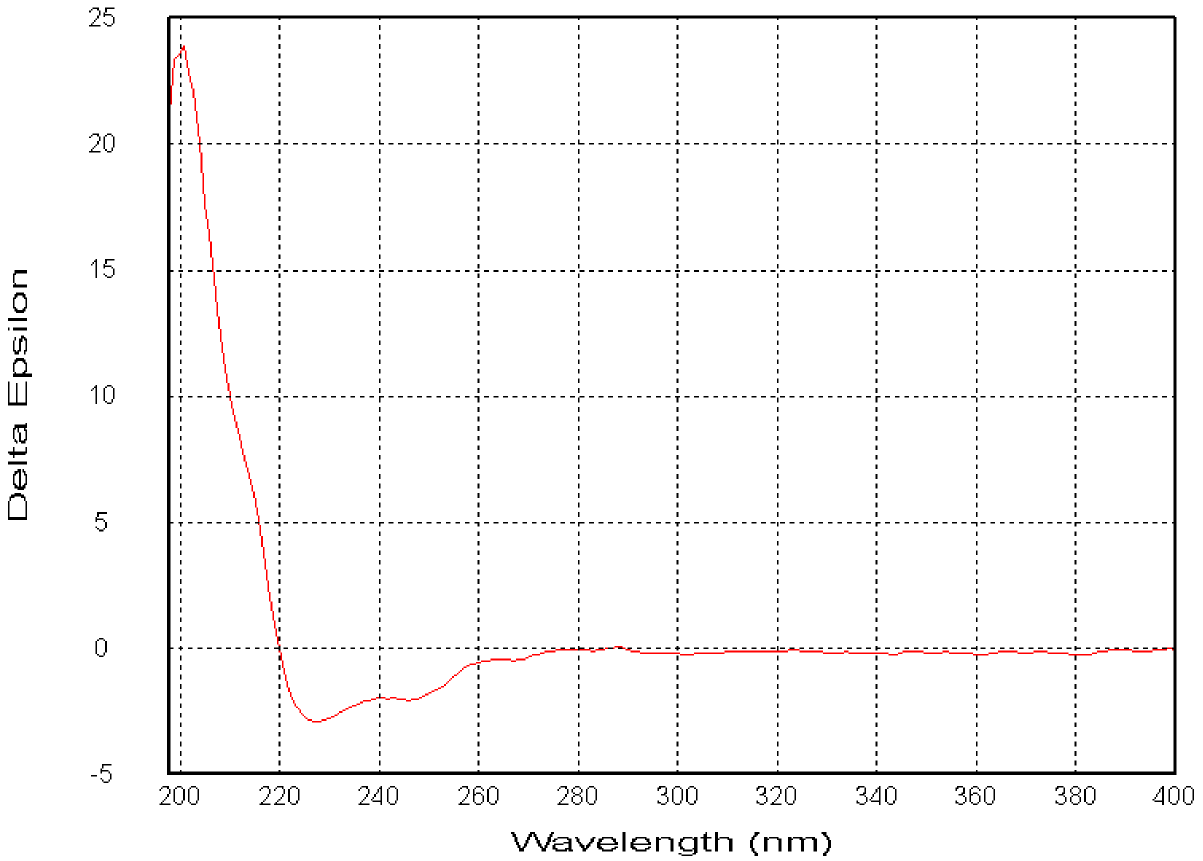

Figure S15. ^{1}H NMR spectrum of **2** measured at 700 MHz in acetone-d_6

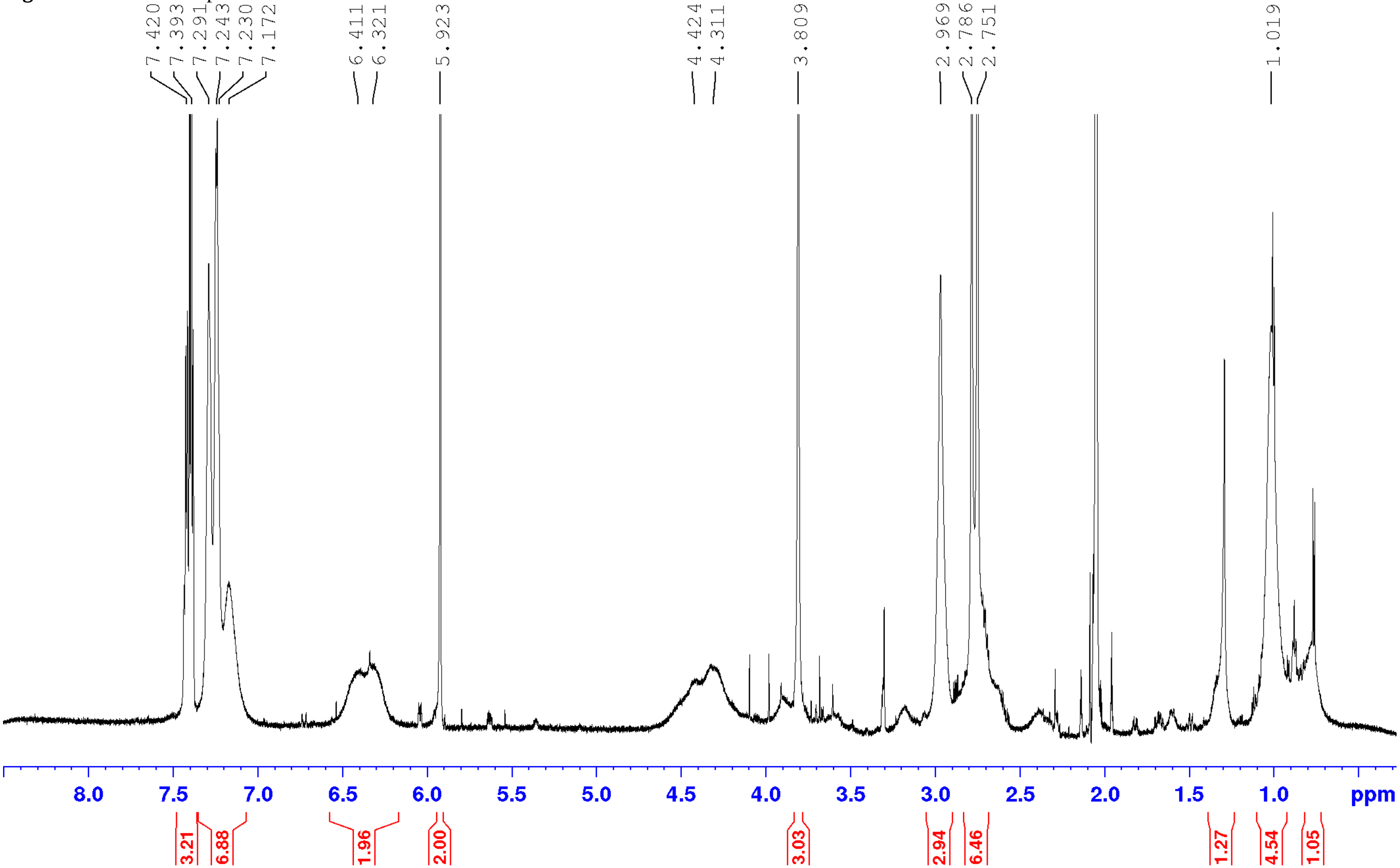

Figure S16. ^{13}C NMR spectrum of **2** measured at 175MHz in acetone-d_6

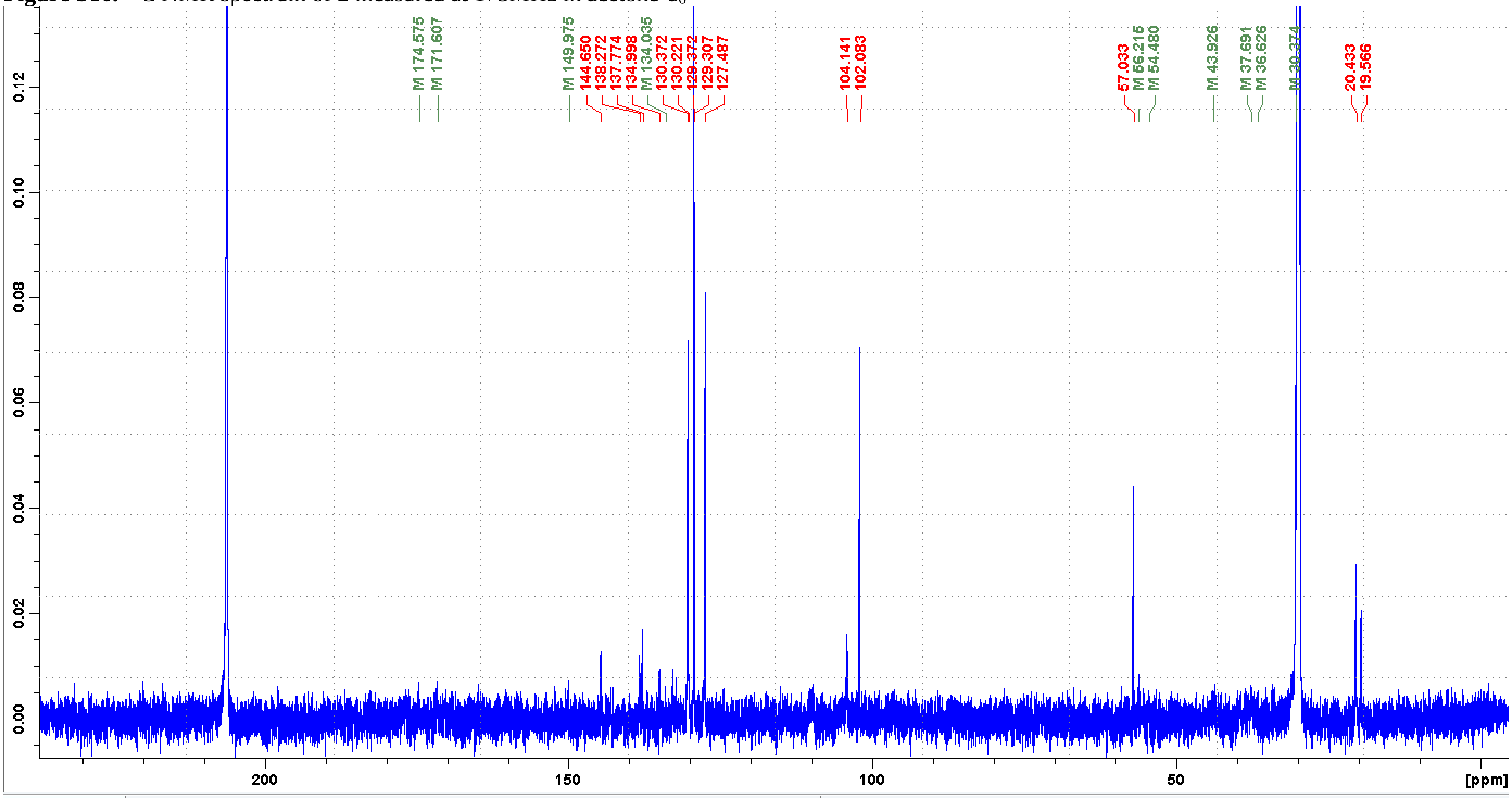

Figure S17. DEPT-135 spectrum of **2** measured at 175 MHz in acetone-d_6

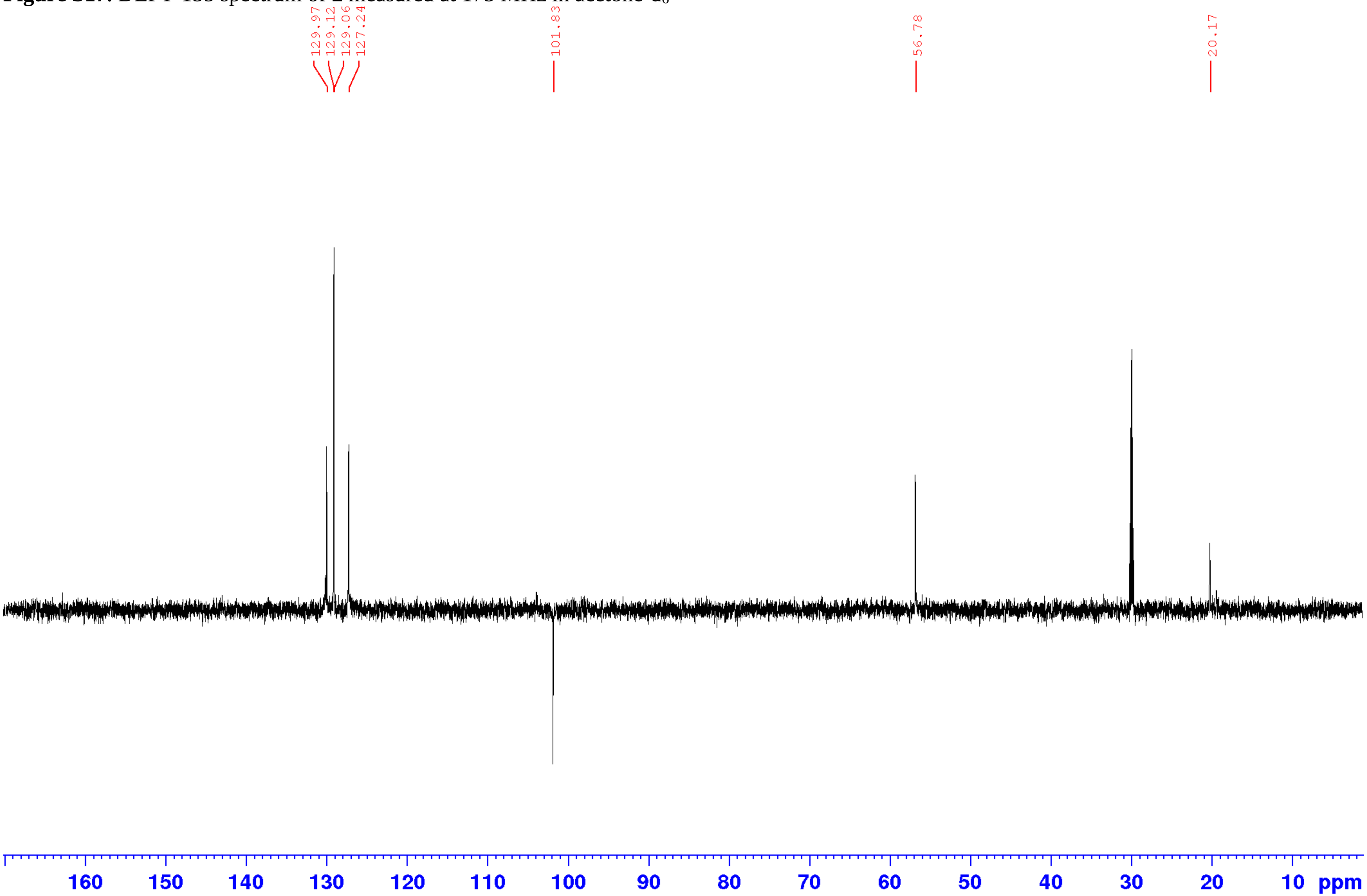

Table S2. ^{13}C and ^{1}H NMR spectroscopic data for saroclazin A (**3**)

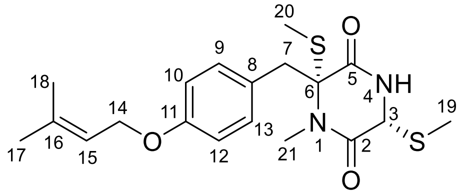

No	3			
	δ_C, type	δ_H, mult. (*J* in Hz)	HMBC	COSY
1	-	-		
2	166.2, C	-		
3	59.3, CH	4.57, d (2.8)	166.2	7.90
4	-	7.90, s	166.1	4.57
5	166.1, C	-		
6	75.9, C	-		
7	42.5, CH_2	3.55, d (14.3)<br>3.20, d (14.3)	75.9; 128.3; 132.3; 166.1	
8	128.3, C	-		
9	132.4, CH	7.12, d (8.6)	42.5, 116.2, 132.5, 159.9	3.55, 6.84
10	116.1, CH	6.84, d (8.6)	116.2, 128.3, 159.9	7.12
11	159.9, C	-		
12	116.1, CH	6.84, d (8.6)	116.2, 128.3, 159.9	7.12
13	132.4, CH	7.12, d (8.6)	42.5, 116.2, 132.5, 159.9	3.55, 6.84
14	66.1, CH_2	4.54, d (4.5) (2H)	121.9, 138.5, 159.9	5.47, 1.76, 1.79
15	121.8, CH	5.46, tt (6.7; 1.2)		4.54, 1.76, 1.79
16	138.4, C	-		
17	26.5, CH_3	1.79, brs	18.9, 121.9, 138.5	
18	18.9, CH_3	1.76, brs	26.5, 121.9, 138.5	
19	16.3, CH_3	2.30, s	59.3	
20	14.3, CH_3	2.22, s	75.9, 31.0	
21	31.0, CH_3	3.18, s	75.9, 166.2	

Chemical shifts were measured at 125 MHz and 500 MHz in acetone-d_6

Figure S18. HRESIMS data for for saroclazin A (**3**)

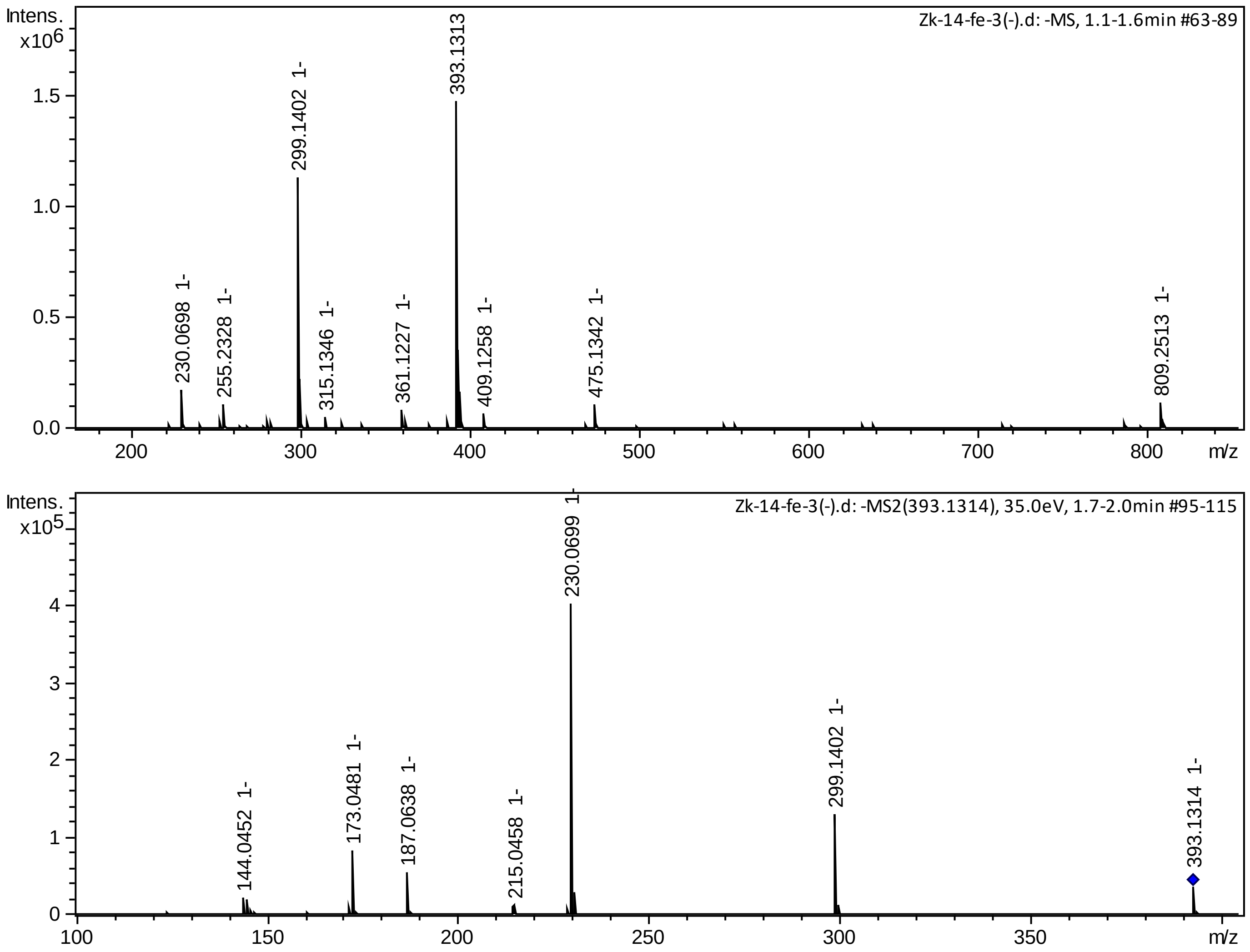

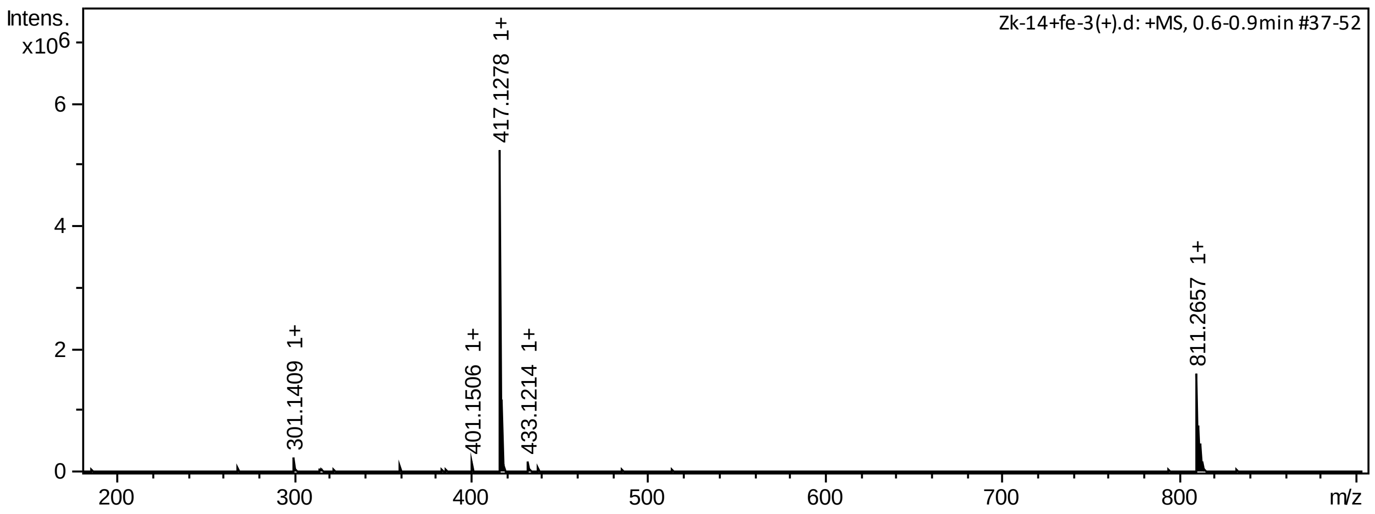

Intens.
x10^6
Zk-14+fe-3(+).d: +MS, 0.6-0.9min #37-52
417.1278 1+
811.2657 1+
301.1409 1+
401.1506 1+
433.1214 1+
m/z

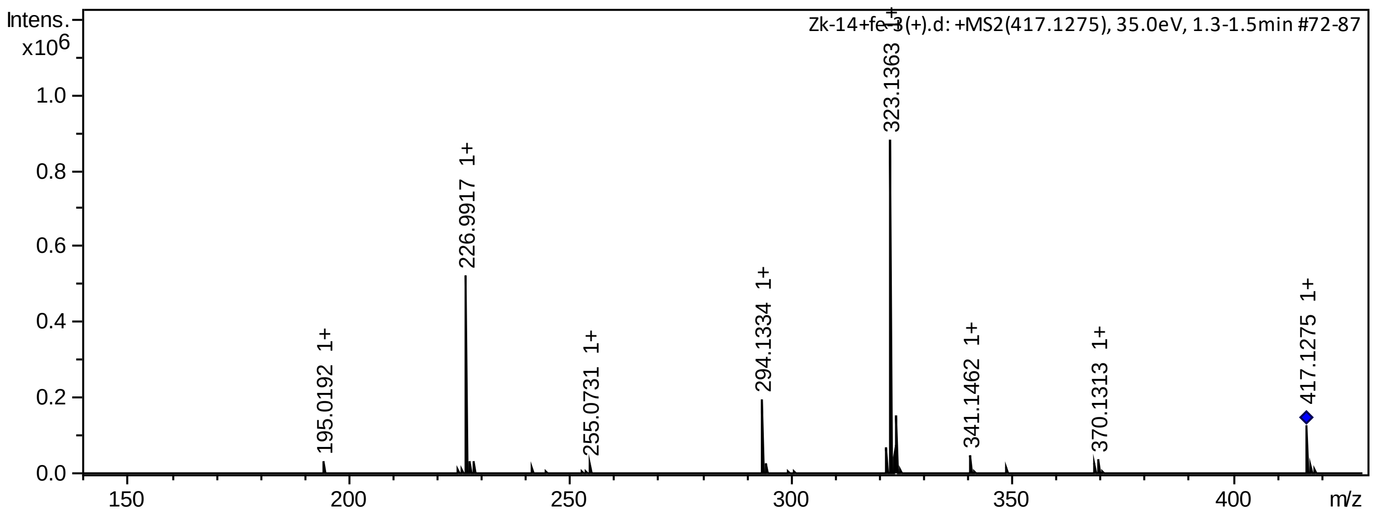

Intens.
x10^6
Zk-14+fe-3(+).d: +MS2(417.1275), 35.0eV, 1.3-1.5min #72-87
323.1363 1+
226.9917 1+
294.1334 1+
195.0192 1+
255.0731 1+
341.1462 1+
370.1313 1+
417.1275 1+
m/z

Figure S19. ^{1}H NMR spectrum of **3** measured at 500 MHz in acetone-d_6

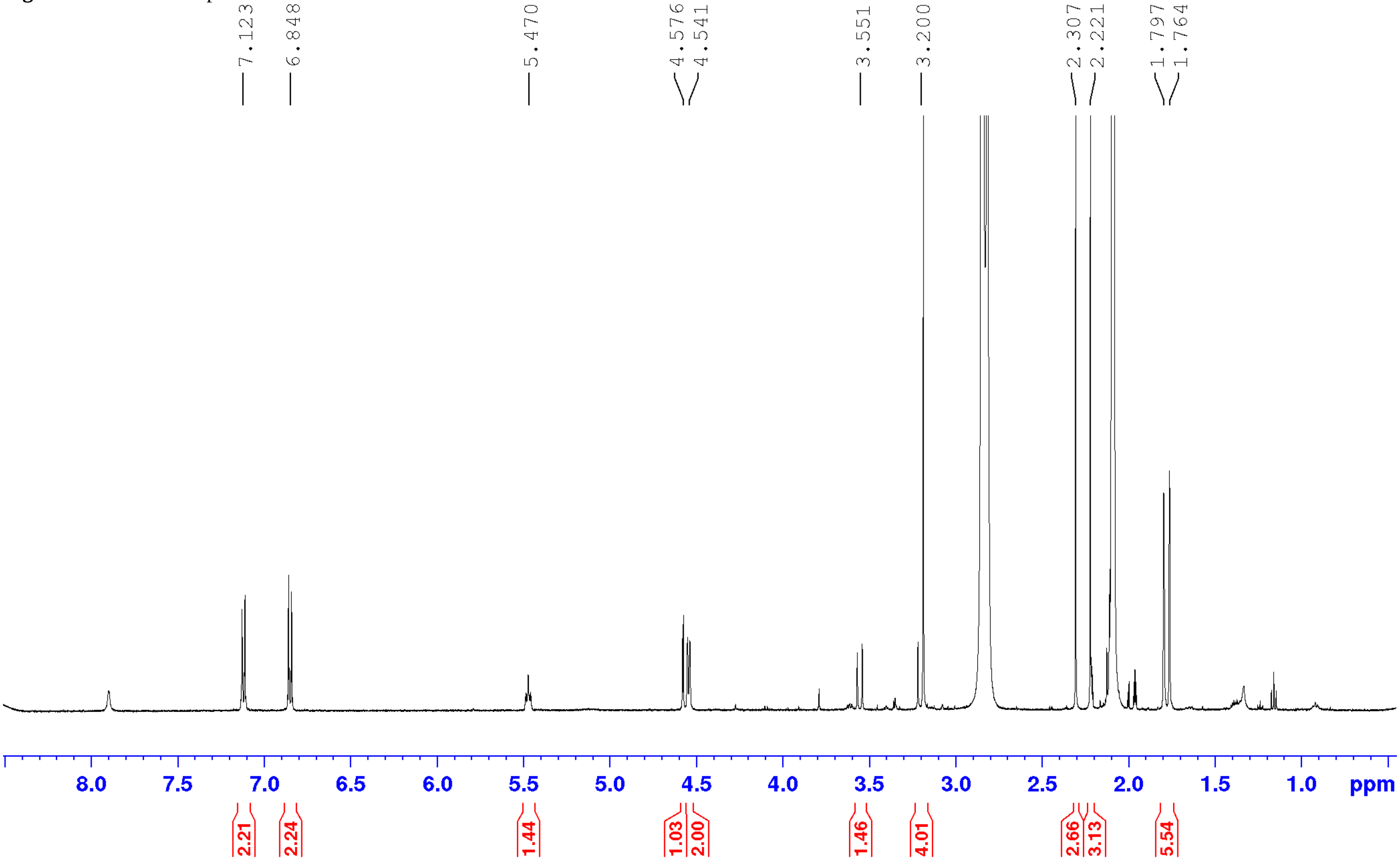

Figure S20. ^{13}C NMR spectrum of 3 measured at 500 MHz in acetone-d_6

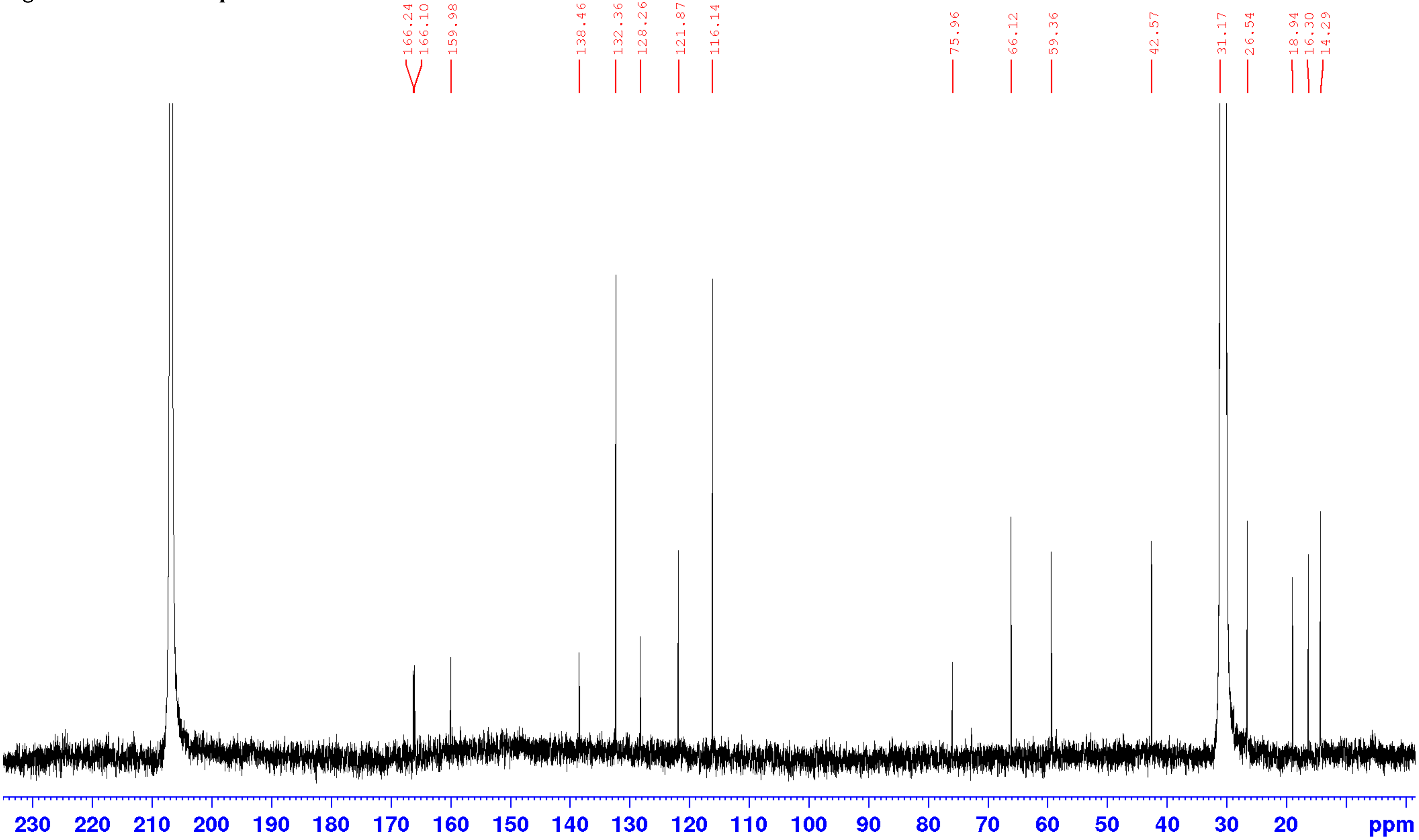

Table S3. ^{13}C and ^{1}H NMR spectroscopic data for (4S,5R,7S)-4,11-dihydroxy-guaia-1(2),9(10)-dien (**4**).

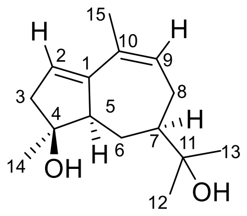

**No**	**4**			
	**δ_C, type**	**δ_H, mult. (*J* in Hz)**	**HMBC**	**COSY**
1	149.3, C			
2	123.8, C	5.54, brs	47.7, 57.1, 82.3, 135.3, 149.3	2.47; 2.36
3	47.7, CH_2	2.47, d (16.7)<br>2.36, dd (16.7, 2.6)	57.1, 82.3, 123.3, 149.3	5.54
4	82.3, C			
5	57.1, CH	2.87, dd (13.0, 3.0)	30.7, 82.3, 123.8, 149.3	1.74, 1.49
6	30.7, CH_2	1.74, m<br>1.49, m	28.2, 50.5, 57.1, 73.6, 82.3, 149.3	2.87, 1.78
7	50.5, CH	1.78, m	28.1, 30.7, 73.6, 130.4	1.49, 2.23
8	28.1, CH_2	2.20-2.26, brm	30.7, 50.5, 73.6, 130.4, 149.3	5.65, 1.78
9	130.4, CH	5.65, q (4.6)	22.4, 28.1	1.81, 2.23
10	135.3, C			
11	73.6, C			
12	26.6, CH_3	1.13. s	28.7, 50.5, 73.6	1.18
13	28.7, CH_3	1.18, s	26.6, 50.5, 73.6	1.13
14	24.8, CH_3	1.28, s	47.6, 57.1, 82.3	
15	22.4, CH_3	1,81, brs	130.4, 135.3, 149.3	5.65
4-OH		3.45, s	24.8, 47.7, 57.1, 82.3	1.28
11-OH		3.13, s	26.6, 28.7, 50.5, 73.6	1.13,

Chemical shifts were measured at 125 MHz and 500 MHz in acetone-d_6

Figure S21. HRESIMS data for **4**

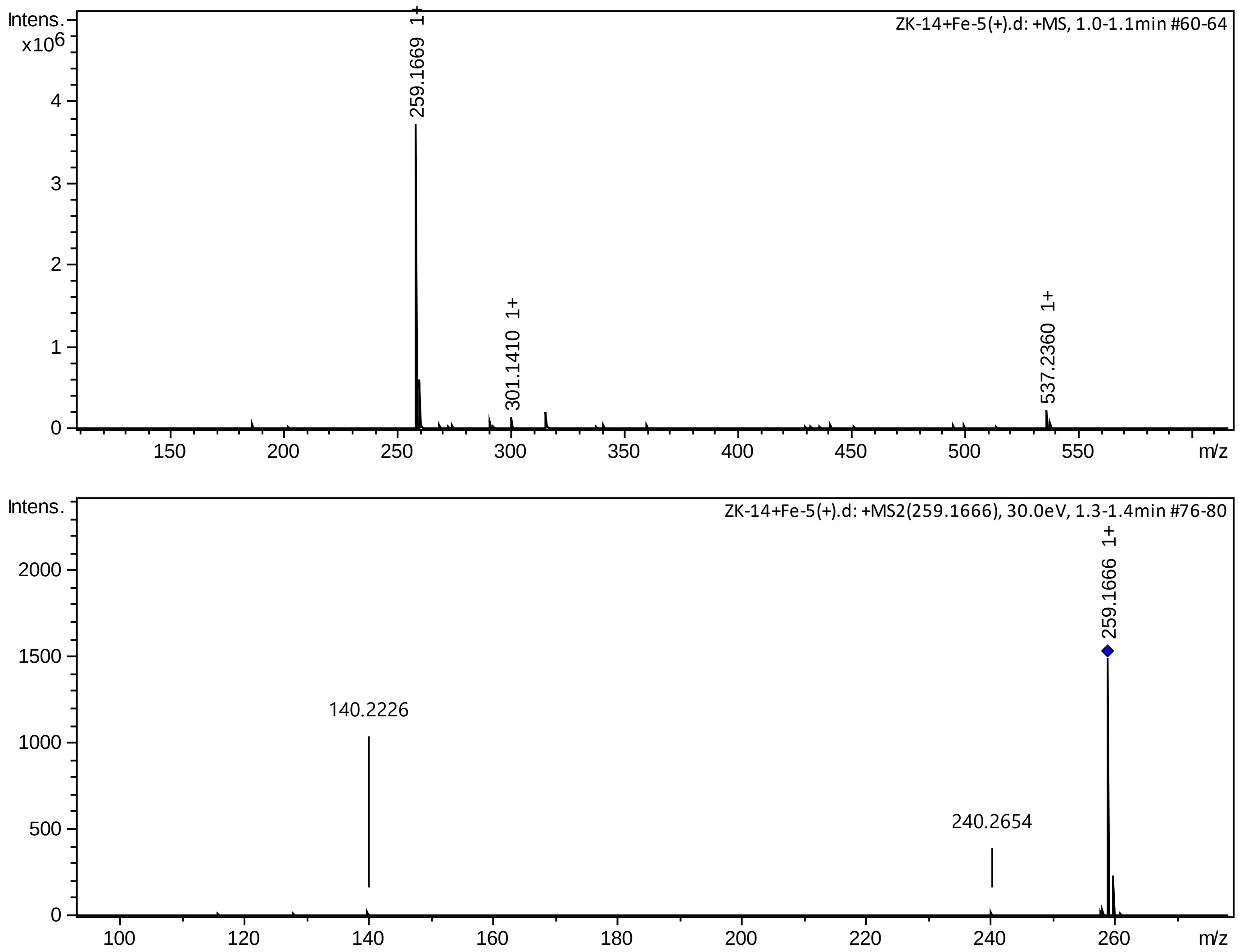

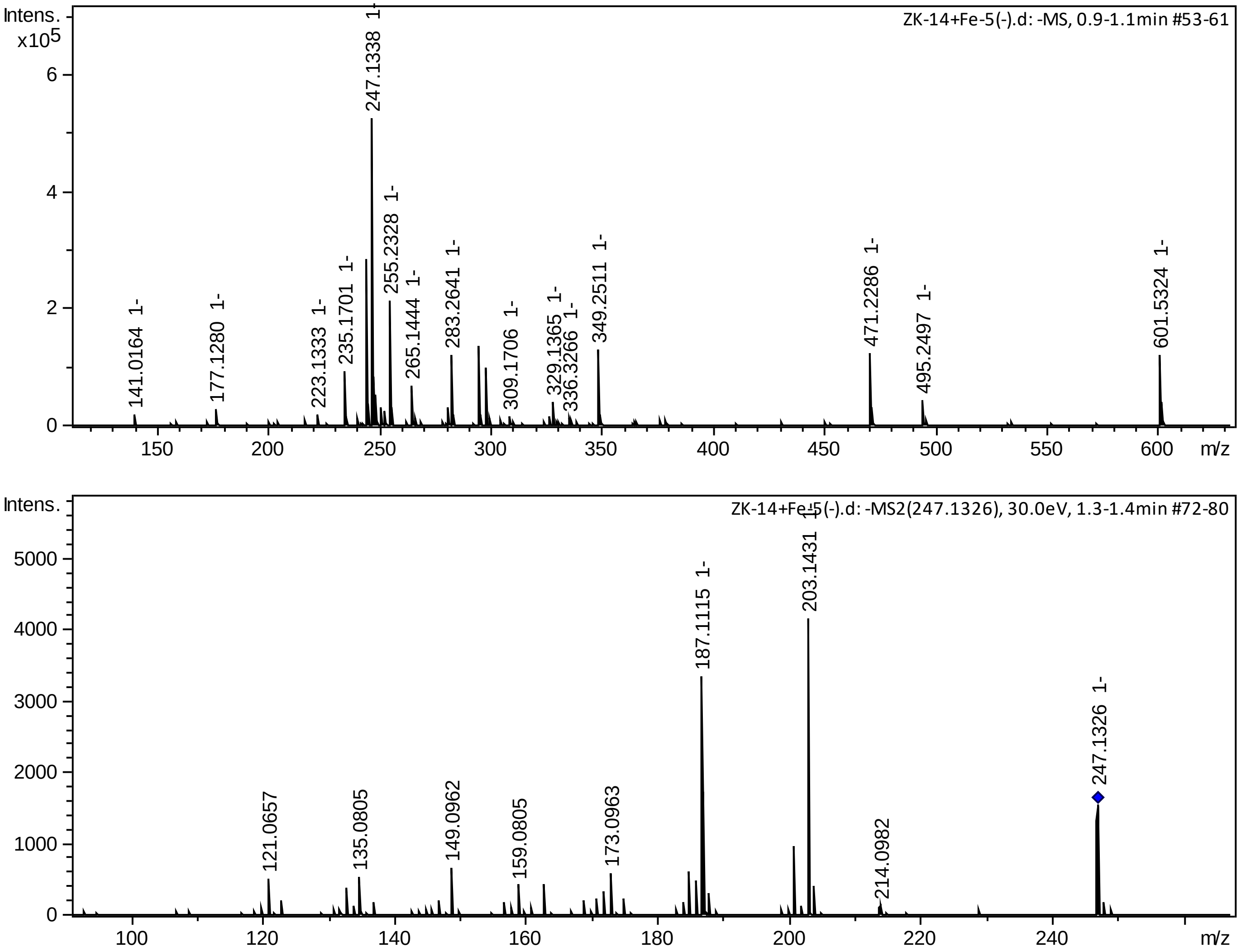
ZK-14+Fe-5(-).d: -MS, 0.9-1.1min #53-61
Intens. x10^5
141.0164 1-
177.1280 1-
223.1333 1-
235.1701 1-
247.1338 1-
255.2328 1-
265.1444 1-
283.2641 1-
309.1706 1-
329.1365 1-
336.3266 1-
349.2511 1-
471.2286 1-
495.2497 1-
601.5324 1-
m/z
ZK-14+Fe-5(-).d: -MS2(247.1326), 30.0eV, 1.3-1.4min #72-80
Intens.
121.0657
135.0805
149.0962
159.0805
173.0963
187.1115 1-
203.1431 1-
214.0982
247.1326 1-
m/z

Figure S22. ^{1}H NMR spectrum of **4** measured at 500 MHz in acetone-d_6

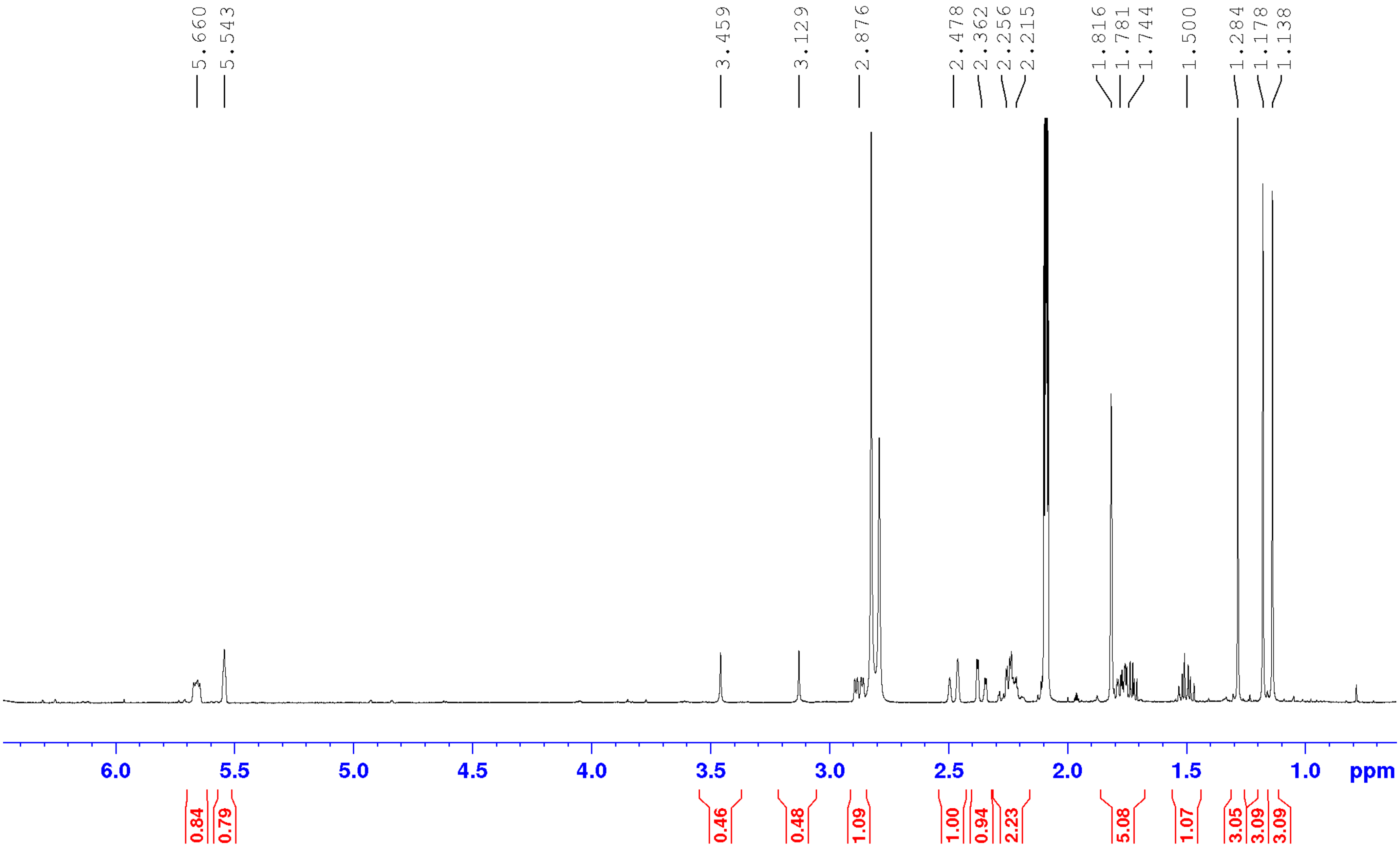

Figure S23. ^{13}C NMR spectrum of **4** measured at 125 MHz in acetone-d_6

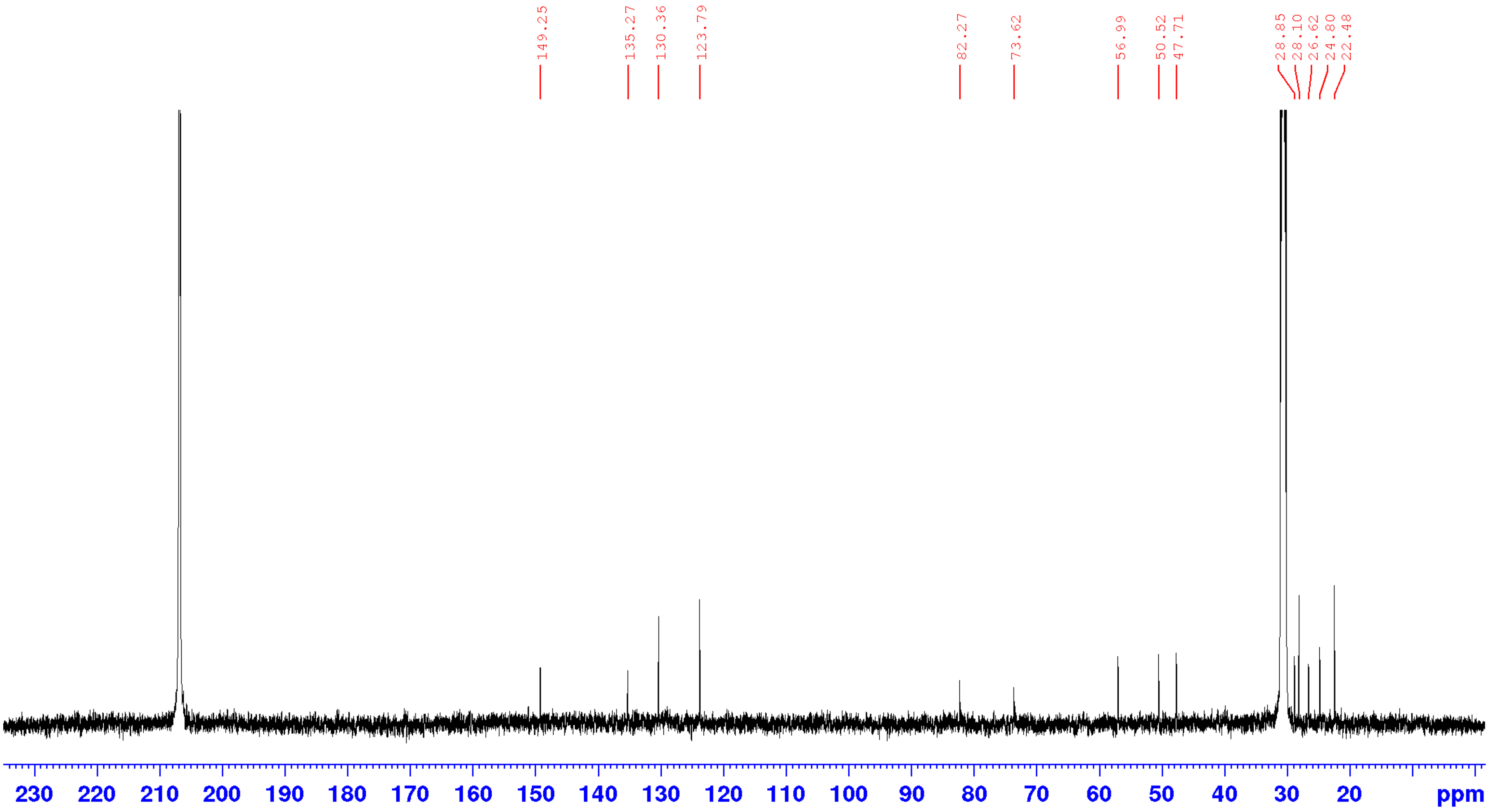

Figure S24. DEPT spectrum of **4** in acetone-d_6

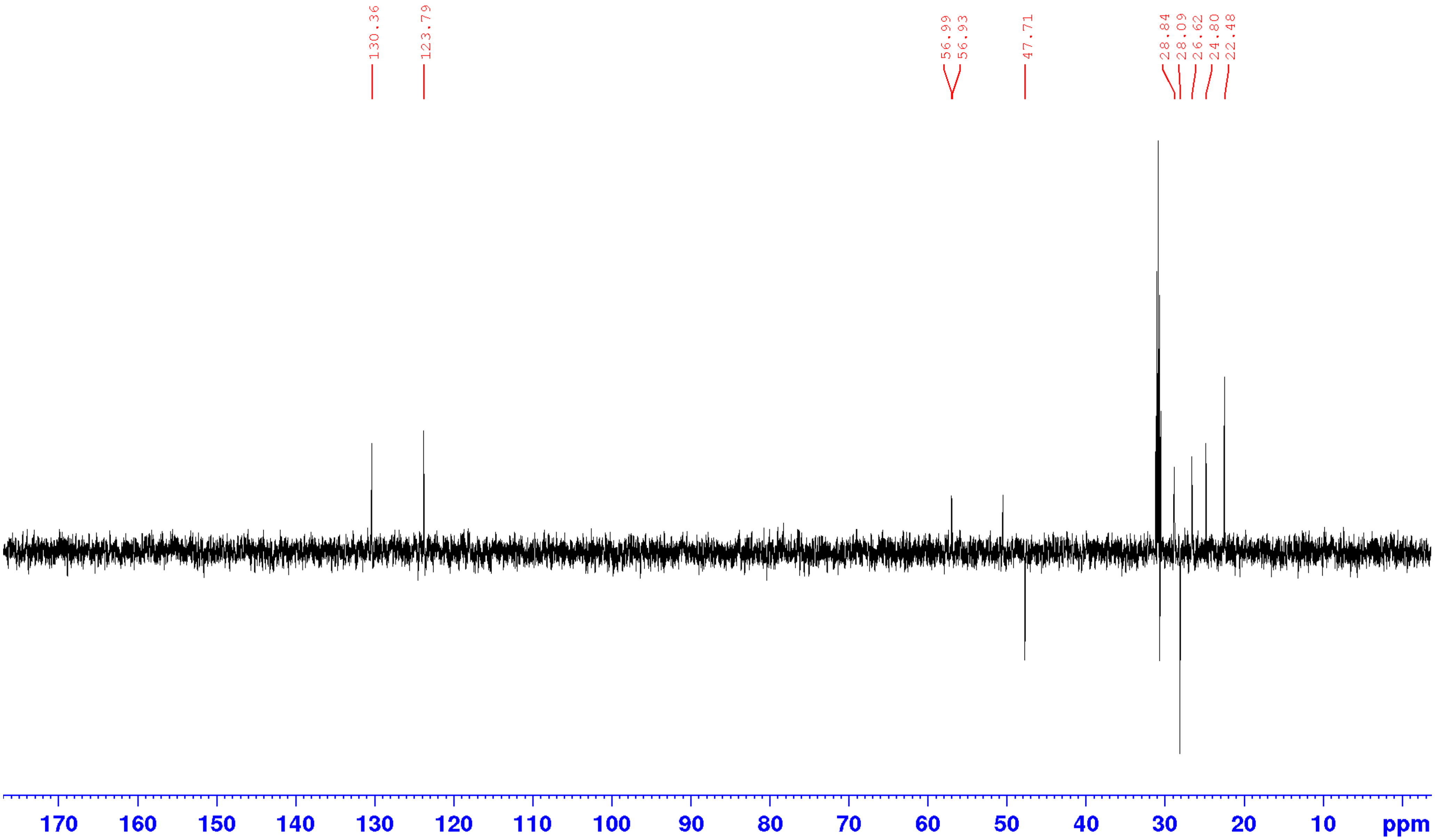

Figure S24. X-ray data of 1

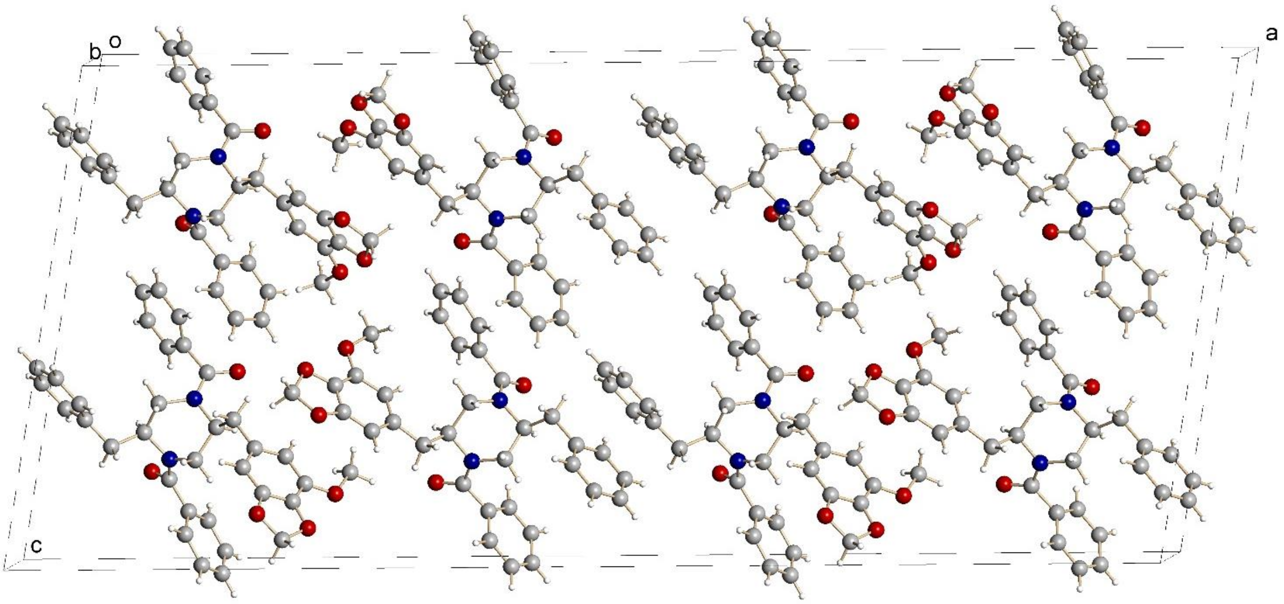

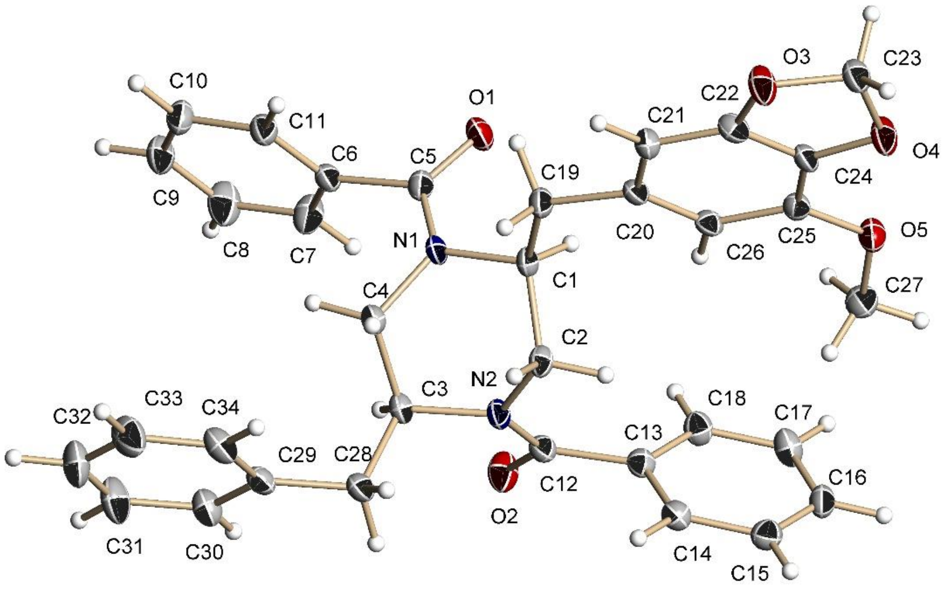

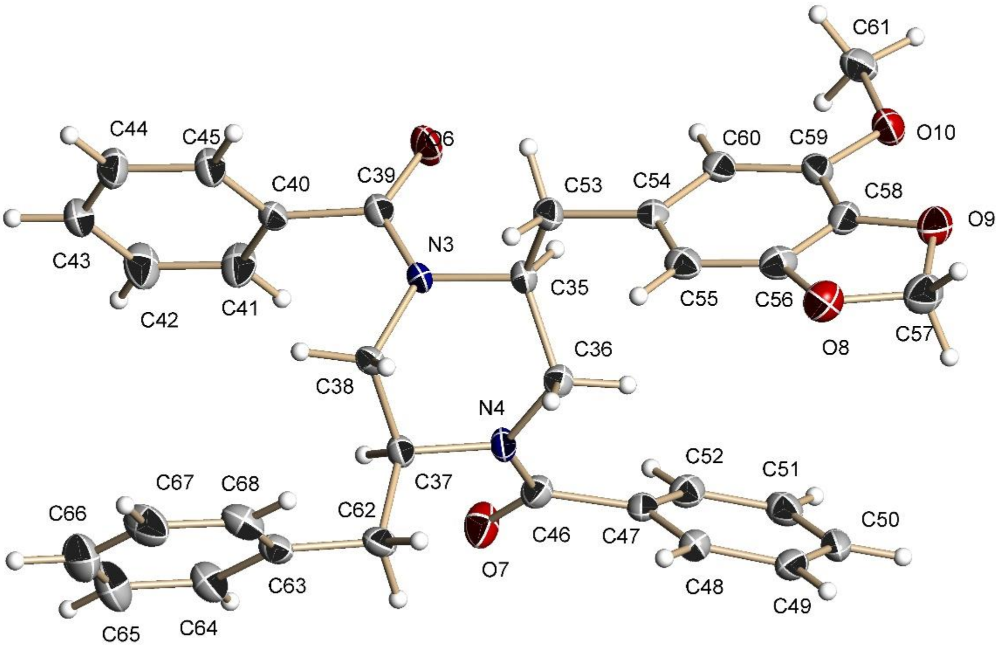
C61
C44
C45
O6
C60
C59
O10
C39
C53
C54
C58
O9
C40
N3
C43
C35
C55
C56
C42
C41
C57
O8
C36
C38
N4
C52
C51
C67
C68
C37
C66
C62
C50
C46
C47
C63
O7
C48
C49
C65
C64

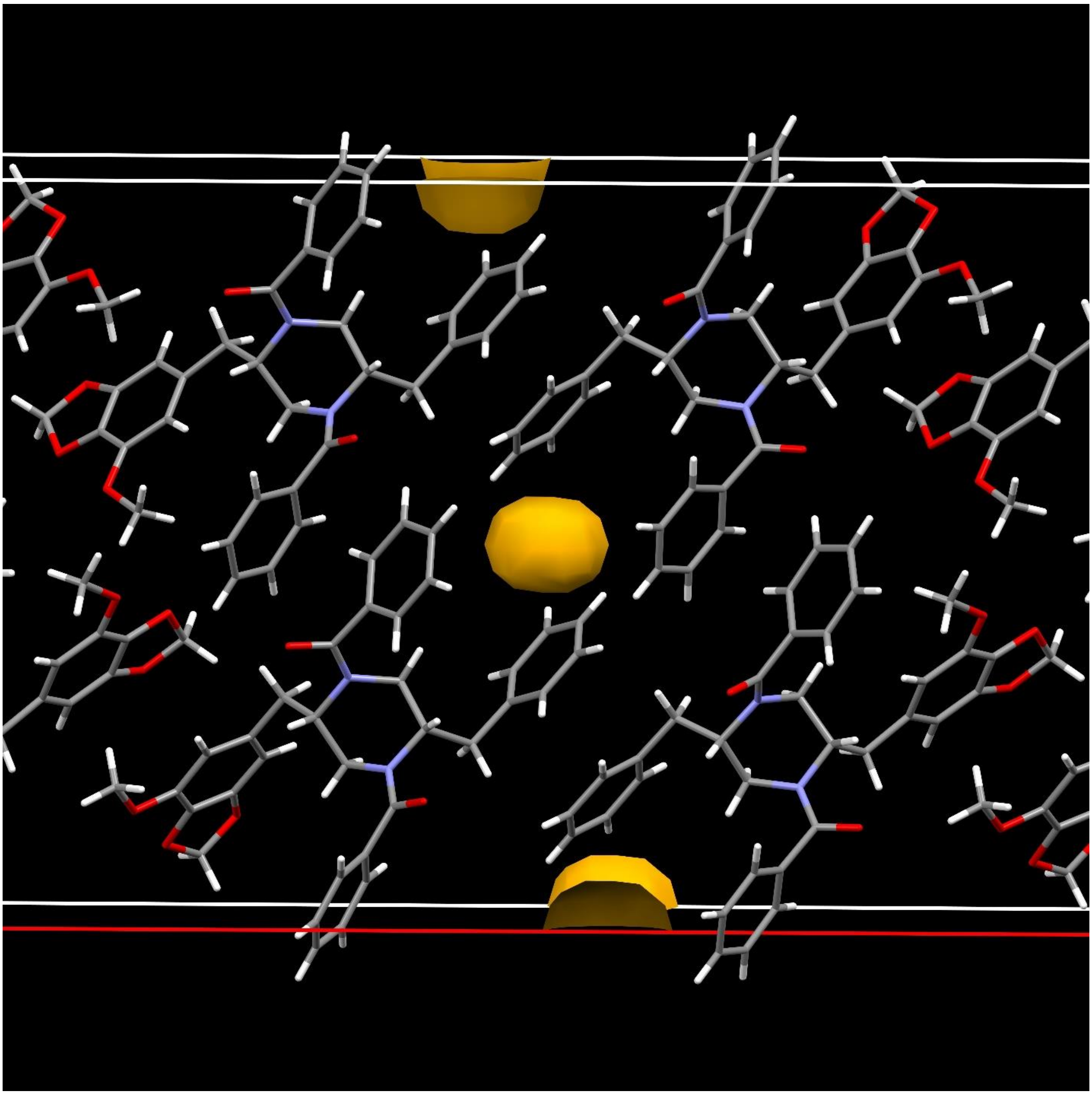

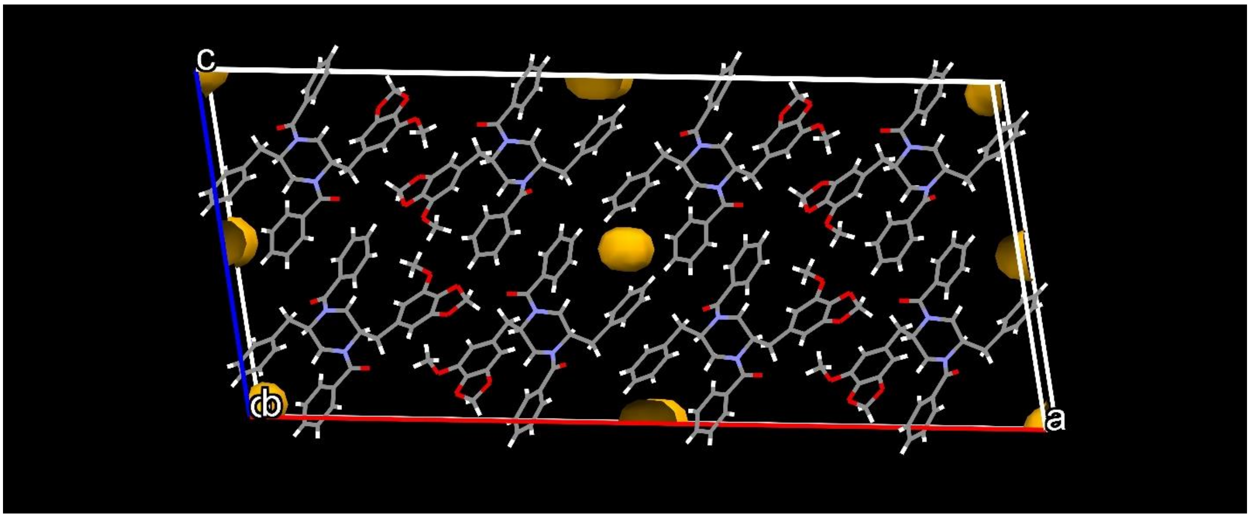
c
cb
a

Table S4. Crystal data and structure refinement for Tib2023_04b_1_0m.

Identification code	Tib2023_04b_1_0m	
Empirical formula	C34 H32 N2 O5	
Formula weight	548.61	
Temperature	100(2) K	
Wavelength	0.71073 E	
Crystal system	Monoclinic	
Space group	C2	
Unit cell dimensions	a = 46.095(4) E	α= 90°.
	b = 6.1121(6) E	β= 99.780(5)°.
	c = 20.2447(19) E	γ = 90°.
Volume	5620.8(9) E^3	
Z	8	
Density (calculated)	1.297 Mg/m^3	
Absorption coefficient	0.087 mm^{-1}	
F(000)	2320	
Crystal size	0.480 x 0.130 x 0.110 mm^3	
Theta range for data collection	1.907 to 27.127°.	
Index ranges	-58<=h<=51, -7<=k<=7, -25<=l<=25	
Reflections collected	44378	
Independent reflections	12335 [R(int) = 0.0374]	
Completeness to theta = 25.242°	99.9 %	
Absorption correction	None	
Refinement method	Full-matrix least-squares on F^2	
Data / restraints / parameters	12335 / 1 / 741	
Goodness-of-fit on F^2	1.029	
Final R indices [I>2sigma(I)]	R1 = 0.0424, wR2 = 0.0957	
R indices (all data)	R1 = 0.0597, wR2 = 0.1050	
Absolute structure parameter	-0.1(3)	
Extinction coefficient	n/a	
Largest diff. peak and hole	0.214 and -0.212 e.E^{-3}	

Table S5. Atomic coordinates (x 10^4) and equivalent isotropic displacement parameters (E^2x 10^3) for Tib2023_04b_1_0m. U(eq) is defined as one third of the trace of the orthogonalized U^{ij} tensor.

	x	y	z	U(eq)
O(1)	8446(1)	3915(3)	8553(1)	28(1)
O(2)	9040(1)	1844(3)	6682(1)	29(1)
O(3)	7491(1)	13329(3)	6994(1)	30(1)
O(4)	7278(1)	10997(3)	6148(1)	30(1)
O(5)	7538(1)	6994(3)	5812(1)	25(1)
N(1)	8762(1)	6009(3)	8091(1)	20(1)
N(2)	8910(1)	5296(3)	6903(1)	20(1)
C(1)	8531(1)	6585(4)	7523(1)	19(1)
C(2)	8678(1)	6940(4)	6906(1)	19(1)
C(3)	9162(1)	5545(4)	7448(1)	19(1)
C(4)	9058(1)	6730(4)	8033(1)	20(1)
C(5)	8696(1)	4638(4)	8569(1)	21(1)
C(6)	8941(1)	4015(4)	9125(1)	20(1)
C(7)	9082(1)	2023(5)	9099(1)	31(1)
C(8)	9298(1)	1379(5)	9627(2)	36(1)
C(9)	9366(1)	2701(5)	10185(1)	31(1)
C(10)	9223(1)	4658(5)	10217(1)	27(1)
C(11)	9012(1)	5345(5)	9683(1)	24(1)
C(12)	8874(1)	3428(4)	6543(1)	19(1)
C(13)	8633(1)	3347(4)	5943(1)	20(1)
C(14)	8622(1)	4854(4)	5424(1)	22(1)
C(15)	8414(1)	4638(5)	4845(1)	24(1)
C(16)	8217(1)	2916(4)	4784(1)	24(1)
C(17)	8222(1)	1433(4)	5303(1)	27(1)
C(18)	8429(1)	1641(4)	5879(1)	24(1)
C(19)	8363(1)	8601(4)	7702(1)	22(1)
C(20)	8078(1)	9159(4)	7234(1)	19(1)
C(21)	7936(1)	11106(4)	7367(1)	22(1)
C(22)	7668(1)	11510(4)	6978(1)	22(1)
C(23)	7222(1)	12817(4)	6558(1)	25(1)
C(24)	7541(1)	10133(4)	6475(1)	21(1)
C(25)	7677(1)	8242(4)	6328(1)	20(1)
C(26)	7951(1)	7766(4)	6723(1)	20(1)
C(27)	7708(1)	5306(5)	5567(1)	27(1)
C(28)	9410(1)	6816(5)	7198(1)	23(1)
C(29)	9664(1)	7274(4)	7752(1)	22(1)

C(30)	9877(1)	5683(5)	7947(1)	28(1)
C(31)	10109(1)	6076(5)	8463(2)	37(1)
C(32)	10127(1)	8055(6)	8801(2)	39(1)
C(33)	9917(1)	9655(5)	8616(2)	36(1)
C(34)	9688(1)	9268(5)	8091(2)	29(1)
O(6)	8425(1)	-1296(4)	3667(1)	35(1)
O(7)	9055(1)	-2913(3)	1658(1)	35(1)
O(8)	7904(1)	8747(3)	1062(1)	33(1)
O(9)	7477(1)	6739(3)	730(1)	30(1)
O(10)	7350(1)	2684(3)	1316(1)	29(1)
N(3)	8726(1)	831(4)	3179(1)	20(1)
N(4)	8868(1)	272(4)	1972(1)	22(1)
C(35)	8489(1)	1419(4)	2625(1)	20(1)
C(36)	8623(1)	1786(4)	1989(1)	21(1)
C(37)	9121(1)	538(4)	2515(1)	21(1)
C(38)	9016(1)	1666(4)	3108(1)	20(1)
C(39)	8672(1)	-546(4)	3663(1)	22(1)
C(40)	8924(1)	-1169(4)	4203(1)	20(1)
C(41)	9088(1)	-3009(5)	4130(1)	34(1)
C(42)	9309(1)	-3674(5)	4640(2)	38(1)
C(43)	9365(1)	-2494(5)	5227(1)	30(1)
C(44)	9202(1)	-652(5)	5306(1)	31(1)
C(45)	8980(1)	7(5)	4791(1)	27(1)
C(46)	8872(1)	-1428(4)	1540(1)	23(1)
C(47)	8659(1)	-1398(4)	894(1)	22(1)
C(48)	8631(1)	449(4)	486(1)	23(1)
C(49)	8445(1)	403(5)	-132(1)	28(1)
C(50)	8288(1)	-1472(5)	-339(1)	28(1)
C(51)	8320(1)	-3324(5)	59(1)	28(1)
C(52)	8508(1)	-3296(5)	670(1)	25(1)
C(53)	8320(1)	3416(4)	2818(1)	24(1)
C(54)	8084(1)	4220(4)	2264(1)	22(1)
C(55)	8131(1)	6168(4)	1933(1)	25(1)
C(56)	7915(1)	6844(5)	1430(1)	26(1)
C(57)	7656(1)	8505(5)	540(1)	34(1)
C(58)	7662(1)	5662(5)	1235(1)	25(1)
C(59)	7609(1)	3744(4)	1546(1)	24(1)
C(60)	7825(1)	3030(4)	2077(1)	23(1)
C(61)	7278(1)	864(5)	1702(1)	30(1)
C(62)	9364(1)	1877(5)	2266(1)	25(1)
C(63)	9622(1)	2270(4)	2815(1)	24(1)

C(64)	9830(1)	625(5)	2995(1)	31(1)
C(65)	10065(1)	941(5)	3512(2)	41(1)
C(66)	10096(1)	2899(6)	3861(2)	42(1)
C(67)	9892(1)	4539(5)	3687(2)	39(1)
C(68)	9659(1)	4234(5)	3165(2)	32(1)

Table S6. Bond lengths [E] and angles [°] for Tib2023_04b_1_0m.

O(1)-C(5)	1.231(3)
O(2)-C(12)	1.239(3)
O(3)-C(22)	1.383(3)
O(3)-C(23)	1.428(3)
O(4)-C(24)	1.383(3)
O(4)-C(23)	1.437(3)
O(5)-C(25)	1.363(3)
O(5)-C(27)	1.435(3)
N(1)-C(5)	1.353(3)
N(1)-C(4)	1.455(3)
N(1)-C(1)	1.471(3)
N(2)-C(12)	1.349(3)
N(2)-C(2)	1.466(3)
N(2)-C(3)	1.470(3)
C(1)-C(19)	1.533(3)
C(1)-C(2)	1.535(3)
C(1)-H(1)	1.0000
C(2)-H(2A)	0.9900
C(2)-H(2B)	0.9900
C(3)-C(4)	1.533(3)
C(3)-C(28)	1.537(3)
C(3)-H(3)	1.0000
C(4)-H(4A)	0.9900
C(4)-H(4B)	0.9900
C(5)-C(6)	1.501(3)
C(6)-C(11)	1.385(3)
C(6)-C(7)	1.387(4)
C(7)-C(8)	1.386(4)
C(7)-H(7)	0.9500
C(8)-C(9)	1.382(4)
C(8)-H(8)	0.9500
C(9)-C(10)	1.372(4)
C(9)-H(9)	0.9500
C(10)-C(11)	1.391(4)
C(10)-H(10)	0.9500
C(11)-H(11)	0.9500
C(12)-C(13)	1.501(3)
C(13)-C(14)	1.392(3)
C(13)-C(18)	1.393(3)

C(14)-C(15)	1.387(4)
C(14)-H(14)	0.9500
C(15)-C(16)	1.384(4)
C(15)-H(15)	0.9500
C(16)-C(17)	1.383(4)
C(16)-H(16)	0.9500
C(17)-C(18)	1.382(3)
C(17)-H(17)	0.9500
C(18)-H(18)	0.9500
C(19)-C(20)	1.520(3)
C(19)-H(19A)	0.9900
C(19)-H(19B)	0.9900
C(20)-C(26)	1.391(3)
C(20)-C(21)	1.405(3)
C(21)-C(22)	1.371(3)
C(21)-H(21)	0.9500
C(22)-C(24)	1.373(4)
C(23)-H(23A)	0.9900
C(23)-H(23B)	0.9900
C(24)-C(25)	1.371(4)
C(25)-C(26)	1.405(3)
C(26)-H(26)	0.9500
C(27)-H(27A)	0.9800
C(27)-H(27B)	0.9800
C(27)-H(27C)	0.9800
C(28)-C(29)	1.504(3)
C(28)-H(28A)	0.9900
C(28)-H(28B)	0.9900
C(29)-C(30)	1.391(4)
C(29)-C(34)	1.394(4)
C(30)-C(31)	1.384(4)
C(30)-H(30)	0.9500
C(31)-C(32)	1.385(4)
C(31)-H(31)	0.9500
C(32)-C(33)	1.383(4)
C(32)-H(32)	0.9500
C(33)-C(34)	1.382(4)
C(33)-H(33)	0.9500
C(34)-H(34)	0.9500
O(6)-C(39)	1.231(3)
O(7)-C(46)	1.235(3)

O(8)-C(56)	1.377(3)
O(8)-C(57)	1.429(3)
O(9)-C(58)	1.384(3)
O(9)-C(57)	1.449(3)
O(10)-C(59)	1.368(3)
O(10)-C(61)	1.430(3)
N(3)-C(39)	1.346(3)
N(3)-C(38)	1.458(3)
N(3)-C(35)	1.471(3)
N(4)-C(46)	1.360(3)
N(4)-C(36)	1.466(3)
N(4)-C(37)	1.469(3)
C(35)-C(53)	1.534(3)
C(35)-C(36)	1.536(3)
C(35)-H(35)	1.0000
C(36)-H(36A)	0.9900
C(36)-H(36B)	0.9900
C(37)-C(38)	1.533(3)
C(37)-C(62)	1.541(3)
C(37)-H(37)	1.0000
C(38)-H(38A)	0.9900
C(38)-H(38B)	0.9900
C(39)-C(40)	1.502(3)
C(40)-C(45)	1.377(4)
C(40)-C(41)	1.378(4)
C(41)-C(42)	1.381(4)
C(41)-H(41)	0.9500
C(42)-C(43)	1.378(4)
C(42)-H(42)	0.9500
C(43)-C(44)	1.377(4)
C(43)-H(43)	0.9500
C(44)-C(45)	1.391(4)
C(44)-H(44)	0.9500
C(45)-H(45)	0.9500
C(46)-C(47)	1.497(4)
C(47)-C(52)	1.388(4)
C(47)-C(48)	1.392(4)
C(48)-C(49)	1.390(4)
C(48)-H(48)	0.9500
C(49)-C(50)	1.382(4)
C(49)-H(49)	0.9500

C(50)-C(51)	1.383(4)
C(50)-H(50)	0.9500
C(51)-C(52)	1.386(4)
C(51)-H(51)	0.9500
C(52)-H(52)	0.9500
C(53)-C(54)	1.506(3)
C(53)-H(53A)	0.9900
C(53)-H(53B)	0.9900
C(54)-C(60)	1.394(4)
C(54)-C(55)	1.401(4)
C(55)-C(56)	1.362(4)
C(55)-H(55)	0.9500
C(56)-C(58)	1.371(4)
C(57)-H(57A)	0.9900
C(57)-H(57B)	0.9900
C(58)-C(59)	1.372(4)
C(59)-C(60)	1.405(4)
C(60)-H(60)	0.9500
C(61)-H(61A)	0.9800
C(61)-H(61B)	0.9800
C(61)-H(61C)	0.9800
C(62)-C(63)	1.502(4)
C(62)-H(62A)	0.9900
C(62)-H(62B)	0.9900
C(63)-C(68)	1.389(4)
C(63)-C(64)	1.394(4)
C(64)-C(65)	1.387(4)
C(64)-H(64)	0.9500
C(65)-C(66)	1.384(4)
C(65)-H(65)	0.9500
C(66)-C(67)	1.378(4)
C(66)-H(66)	0.9500
C(67)-C(68)	1.386(4)
C(67)-H(67)	0.9500
C(68)-H(68)	0.9500

C(22)-O(3)-C(23)	105.24(19)
C(24)-O(4)-C(23)	104.33(19)
C(25)-O(5)-C(27)	116.88(19)
C(5)-N(1)-C(4)	124.9(2)
C(5)-N(1)-C(1)	119.1(2)

C(4)-N(1)-C(1)	115.29(19)
C(12)-N(2)-C(2)	124.0(2)
C(12)-N(2)-C(3)	120.2(2)
C(2)-N(2)-C(3)	114.14(19)
N(1)-C(1)-C(19)	109.61(19)
N(1)-C(1)-C(2)	107.96(18)
C(19)-C(1)-C(2)	113.0(2)
N(1)-C(1)-H(1)	108.7
C(19)-C(1)-H(1)	108.7
C(2)-C(1)-H(1)	108.7
N(2)-C(2)-C(1)	109.2(2)
N(2)-C(2)-H(2A)	109.8
C(1)-C(2)-H(2A)	109.8
N(2)-C(2)-H(2B)	109.8
C(1)-C(2)-H(2B)	109.8
H(2A)-C(2)-H(2B)	108.3
N(2)-C(3)-C(4)	108.43(19)
N(2)-C(3)-C(28)	110.02(19)
C(4)-C(3)-C(28)	111.4(2)
N(2)-C(3)-H(3)	109.0
C(4)-C(3)-H(3)	109.0
C(28)-C(3)-H(3)	109.0
N(1)-C(4)-C(3)	109.9(2)
N(1)-C(4)-H(4A)	109.7
C(3)-C(4)-H(4A)	109.7
N(1)-C(4)-H(4B)	109.7
C(3)-C(4)-H(4B)	109.7
H(4A)-C(4)-H(4B)	108.2
O(1)-C(5)-N(1)	121.8(2)
O(1)-C(5)-C(6)	120.7(2)
N(1)-C(5)-C(6)	117.6(2)
C(11)-C(6)-C(7)	120.0(2)
C(11)-C(6)-C(5)	120.4(2)
C(7)-C(6)-C(5)	119.5(2)
C(8)-C(7)-C(6)	120.0(3)
C(8)-C(7)-H(7)	120.0
C(6)-C(7)-H(7)	120.0
C(9)-C(8)-C(7)	119.9(3)
C(9)-C(8)-H(8)	120.0
C(7)-C(8)-H(8)	120.0
C(10)-C(9)-C(8)	120.2(3)

C(10)-C(9)-H(9)	119.9
C(8)-C(9)-H(9)	119.9
C(9)-C(10)-C(11)	120.4(3)
C(9)-C(10)-H(10)	119.8
C(11)-C(10)-H(10)	119.8
C(6)-C(11)-C(10)	119.6(2)
C(6)-C(11)-H(11)	120.2
C(10)-C(11)-H(11)	120.2
O(2)-C(12)-N(2)	121.6(2)
O(2)-C(12)-C(13)	120.3(2)
N(2)-C(12)-C(13)	118.1(2)
C(14)-C(13)-C(18)	119.2(2)
C(14)-C(13)-C(12)	121.1(2)
C(18)-C(13)-C(12)	119.6(2)
C(15)-C(14)-C(13)	120.5(2)
C(15)-C(14)-H(14)	119.8
C(13)-C(14)-H(14)	119.8
C(16)-C(15)-C(14)	119.7(2)
C(16)-C(15)-H(15)	120.2
C(14)-C(15)-H(15)	120.2
C(17)-C(16)-C(15)	120.4(2)
C(17)-C(16)-H(16)	119.8
C(15)-C(16)-H(16)	119.8
C(18)-C(17)-C(16)	120.0(2)
C(18)-C(17)-H(17)	120.0
C(16)-C(17)-H(17)	120.0
C(17)-C(18)-C(13)	120.3(2)
C(17)-C(18)-H(18)	119.8
C(13)-C(18)-H(18)	119.8
C(20)-C(19)-C(1)	116.8(2)
C(20)-C(19)-H(19A)	108.1
C(1)-C(19)-H(19A)	108.1
C(20)-C(19)-H(19B)	108.1
C(1)-C(19)-H(19B)	108.1
H(19A)-C(19)-H(19B)	107.3
C(26)-C(20)-C(21)	120.6(2)
C(26)-C(20)-C(19)	122.4(2)
C(21)-C(20)-C(19)	116.8(2)
C(22)-C(21)-C(20)	116.5(2)
C(22)-C(21)-H(21)	121.8
C(20)-C(21)-H(21)	121.8

C(21)-C(22)-C(24)	123.1(2)
C(21)-C(22)-O(3)	127.5(2)
C(24)-C(22)-O(3)	109.3(2)
O(3)-C(23)-O(4)	107.76(19)
O(3)-C(23)-H(23A)	110.2
O(4)-C(23)-H(23A)	110.2
O(3)-C(23)-H(23B)	110.2
O(4)-C(23)-H(23B)	110.2
H(23A)-C(23)-H(23B)	108.5
C(25)-C(24)-C(22)	121.6(2)
C(25)-C(24)-O(4)	127.9(2)
C(22)-C(24)-O(4)	110.4(2)
O(5)-C(25)-C(24)	117.6(2)
O(5)-C(25)-C(26)	125.5(2)
C(24)-C(25)-C(26)	116.8(2)
C(20)-C(26)-C(25)	121.4(2)
C(20)-C(26)-H(26)	119.3
C(25)-C(26)-H(26)	119.3
O(5)-C(27)-H(27A)	109.5
O(5)-C(27)-H(27B)	109.5
H(27A)-C(27)-H(27B)	109.5
O(5)-C(27)-H(27C)	109.5
H(27A)-C(27)-H(27C)	109.5
H(27B)-C(27)-H(27C)	109.5
C(29)-C(28)-C(3)	112.0(2)
C(29)-C(28)-H(28A)	109.2
C(3)-C(28)-H(28A)	109.2
C(29)-C(28)-H(28B)	109.2
C(3)-C(28)-H(28B)	109.2
H(28A)-C(28)-H(28B)	107.9
C(30)-C(29)-C(34)	118.5(3)
C(30)-C(29)-C(28)	120.3(2)
C(34)-C(29)-C(28)	121.2(2)
C(31)-C(30)-C(29)	120.7(3)
C(31)-C(30)-H(30)	119.6
C(29)-C(30)-H(30)	119.6
C(30)-C(31)-C(32)	119.9(3)
C(30)-C(31)-H(31)	120.1
C(32)-C(31)-H(31)	120.1
C(33)-C(32)-C(31)	120.2(3)
C(33)-C(32)-H(32)	119.9

C(31)-C(32)-H(32)	119.9
C(34)-C(33)-C(32)	119.7(3)
C(34)-C(33)-H(33)	120.2
C(32)-C(33)-H(33)	120.2
C(33)-C(34)-C(29)	121.0(3)
C(33)-C(34)-H(34)	119.5
C(29)-C(34)-H(34)	119.5
C(56)-O(8)-C(57)	105.3(2)
C(58)-O(9)-C(57)	104.0(2)
C(59)-O(10)-C(61)	116.8(2)
C(39)-N(3)-C(38)	125.1(2)
C(39)-N(3)-C(35)	120.1(2)
C(38)-N(3)-C(35)	114.43(19)
C(46)-N(4)-C(36)	126.4(2)
C(46)-N(4)-C(37)	118.0(2)
C(36)-N(4)-C(37)	115.4(2)
N(3)-C(35)-C(53)	110.2(2)
N(3)-C(35)-C(36)	108.85(19)
C(53)-C(35)-C(36)	113.0(2)
N(3)-C(35)-H(35)	108.2
C(53)-C(35)-H(35)	108.2
C(36)-C(35)-H(35)	108.2
N(4)-C(36)-C(35)	110.3(2)
N(4)-C(36)-H(36A)	109.6
C(35)-C(36)-H(36A)	109.6
N(4)-C(36)-H(36B)	109.6
C(35)-C(36)-H(36B)	109.6
H(36A)-C(36)-H(36B)	108.1
N(4)-C(37)-C(38)	108.66(19)
N(4)-C(37)-C(62)	110.2(2)
C(38)-C(37)-C(62)	111.2(2)
N(4)-C(37)-H(37)	108.9
C(38)-C(37)-H(37)	108.9
C(62)-C(37)-H(37)	108.9
N(3)-C(38)-C(37)	109.6(2)
N(3)-C(38)-H(38A)	109.7
C(37)-C(38)-H(38A)	109.7
N(3)-C(38)-H(38B)	109.7
C(37)-C(38)-H(38B)	109.7
H(38A)-C(38)-H(38B)	108.2
O(6)-C(39)-N(3)	121.6(2)

O(6)-C(39)-C(40)	120.2(2)
N(3)-C(39)-C(40)	118.3(2)
C(45)-C(40)-C(41)	119.5(2)
C(45)-C(40)-C(39)	120.4(2)
C(41)-C(40)-C(39)	119.9(2)
C(40)-C(41)-C(42)	120.5(3)
C(40)-C(41)-H(41)	119.7
C(42)-C(41)-H(41)	119.7
C(43)-C(42)-C(41)	119.9(3)
C(43)-C(42)-H(42)	120.0
C(41)-C(42)-H(42)	120.0
C(44)-C(43)-C(42)	120.0(3)
C(44)-C(43)-H(43)	120.0
C(42)-C(43)-H(43)	120.0
C(43)-C(44)-C(45)	119.8(3)
C(43)-C(44)-H(44)	120.1
C(45)-C(44)-H(44)	120.1
C(40)-C(45)-C(44)	120.2(3)
C(40)-C(45)-H(45)	119.9
C(44)-C(45)-H(45)	119.9
O(7)-C(46)-N(4)	121.4(2)
O(7)-C(46)-C(47)	120.2(2)
N(4)-C(46)-C(47)	118.3(2)
C(52)-C(47)-C(48)	119.5(2)
C(52)-C(47)-C(46)	119.5(2)
C(48)-C(47)-C(46)	120.7(2)
C(49)-C(48)-C(47)	120.0(3)
C(49)-C(48)-H(48)	120.0
C(47)-C(48)-H(48)	120.0
C(50)-C(49)-C(48)	120.0(3)
C(50)-C(49)-H(49)	120.0
C(48)-C(49)-H(49)	120.0
C(49)-C(50)-C(51)	120.3(3)
C(49)-C(50)-H(50)	119.9
C(51)-C(50)-H(50)	119.9
C(50)-C(51)-C(52)	119.9(3)
C(50)-C(51)-H(51)	120.1
C(52)-C(51)-H(51)	120.1
C(51)-C(52)-C(47)	120.4(3)
C(51)-C(52)-H(52)	119.8
C(47)-C(52)-H(52)	119.8

C(54)-C(53)-C(35)	113.8(2)
C(54)-C(53)-H(53A)	108.8
C(35)-C(53)-H(53A)	108.8
C(54)-C(53)-H(53B)	108.8
C(35)-C(53)-H(53B)	108.8
H(53A)-C(53)-H(53B)	107.7
C(60)-C(54)-C(55)	120.5(2)
C(60)-C(54)-C(53)	120.5(2)
C(55)-C(54)-C(53)	118.9(2)
C(56)-C(55)-C(54)	117.6(2)
C(56)-C(55)-H(55)	121.2
C(54)-C(55)-H(55)	121.2
C(55)-C(56)-C(58)	122.2(3)
C(55)-C(56)-O(8)	128.0(3)
C(58)-C(56)-O(8)	109.8(2)
O(8)-C(57)-O(9)	107.5(2)
O(8)-C(57)-H(57A)	110.2
O(9)-C(57)-H(57A)	110.2
O(8)-C(57)-H(57B)	110.2
O(9)-C(57)-H(57B)	110.2
H(57A)-C(57)-H(57B)	108.5
C(56)-C(58)-C(59)	121.8(2)
C(56)-C(58)-O(9)	110.5(2)
C(59)-C(58)-O(9)	127.6(2)
O(10)-C(59)-C(58)	117.4(2)
O(10)-C(59)-C(60)	125.3(2)
C(58)-C(59)-C(60)	117.2(2)
C(54)-C(60)-C(59)	120.6(3)
C(54)-C(60)-H(60)	119.7
C(59)-C(60)-H(60)	119.7
O(10)-C(61)-H(61A)	109.5
O(10)-C(61)-H(61B)	109.5
H(61A)-C(61)-H(61B)	109.5
O(10)-C(61)-H(61C)	109.5
H(61A)-C(61)-H(61C)	109.5
H(61B)-C(61)-H(61C)	109.5
C(63)-C(62)-C(37)	111.9(2)
C(63)-C(62)-H(62A)	109.2
C(37)-C(62)-H(62A)	109.2
C(63)-C(62)-H(62B)	109.2
C(37)-C(62)-H(62B)	109.2

H(62A)-C(62)-H(62B)	107.9
C(68)-C(63)-C(64)	118.1(3)
C(68)-C(63)-C(62)	121.9(2)
C(64)-C(63)-C(62)	120.0(2)
C(65)-C(64)-C(63)	120.7(3)
C(65)-C(64)-H(64)	119.6
C(63)-C(64)-H(64)	119.6
C(66)-C(65)-C(64)	120.3(3)
C(66)-C(65)-H(65)	119.8
C(64)-C(65)-H(65)	119.8
C(67)-C(66)-C(65)	119.5(3)
C(67)-C(66)-H(66)	120.2
C(65)-C(66)-H(66)	120.2
C(66)-C(67)-C(68)	120.2(3)
C(66)-C(67)-H(67)	119.9
C(68)-C(67)-H(67)	119.9
C(67)-C(68)-C(63)	121.2(3)
C(67)-C(68)-H(68)	119.4
C(63)-C(68)-H(68)	119.4

Symmetry transformations used to generate equivalent atoms:

Table S7. Anisotropic displacement parameters (E^2x 10^3) for Tib2023_04b_1_0m.

The anisotropic displacement factor exponent takes the form: $-2p^2[h^2 a^{*2}U^{11} + ... + 2 h k a^* b^* U^{12}]$

	U^{11}	U^{22}	U^{33}	U^{23}	U^{13}	U^{12}
O(1)	16(1)	39(1)	28(1)	6(1)	1(1)	-4(1)
O(2)	28(1)	25(1)	32(1)	-3(1)	-4(1)	9(1)
O(3)	20(1)	31(1)	36(1)	-8(1)	-4(1)	8(1)
O(4)	18(1)	34(1)	34(1)	-8(1)	-5(1)	7(1)
O(5)	20(1)	27(1)	25(1)	-5(1)	-1(1)	2(1)
N(1)	11(1)	27(1)	20(1)	2(1)	-1(1)	1(1)
N(2)	14(1)	23(1)	20(1)	0(1)	-1(1)	3(1)
C(1)	14(1)	24(1)	18(1)	0(1)	-2(1)	1(1)
C(2)	18(1)	19(1)	19(1)	-1(1)	-2(1)	4(1)
C(3)	14(1)	20(1)	22(1)	0(1)	-1(1)	0(1)
C(4)	15(1)	24(1)	21(1)	-1(1)	1(1)	-2(1)
C(5)	19(1)	23(1)	19(1)	-3(1)	0(1)	2(1)
C(6)	15(1)	24(1)	20(1)	2(1)	2(1)	0(1)
C(7)	31(2)	30(2)	27(2)	-7(1)	-4(1)	8(1)
C(8)	32(2)	32(2)	40(2)	-2(1)	-5(1)	12(1)
C(9)	23(2)	37(2)	28(2)	4(1)	-4(1)	4(1)
C(10)	23(2)	33(2)	22(2)	-5(1)	-2(1)	1(1)
C(11)	19(1)	27(1)	24(1)	-2(1)	1(1)	3(1)
C(12)	17(1)	21(1)	21(1)	-1(1)	4(1)	1(1)
C(13)	18(1)	20(1)	22(1)	-2(1)	3(1)	3(1)
C(14)	20(1)	23(1)	24(2)	1(1)	4(1)	0(1)
C(15)	24(1)	28(1)	22(1)	4(1)	4(1)	4(1)
C(16)	19(1)	29(2)	22(1)	-5(1)	-2(1)	3(1)
C(17)	20(1)	24(1)	34(2)	0(1)	-2(1)	-2(1)
C(18)	22(1)	23(1)	26(1)	4(1)	0(1)	1(1)
C(19)	17(1)	29(1)	20(1)	-4(1)	-1(1)	3(1)
C(20)	14(1)	23(1)	19(1)	2(1)	3(1)	2(1)
C(21)	18(1)	25(1)	21(1)	-1(1)	0(1)	-1(1)
C(22)	19(1)	22(1)	27(2)	0(1)	6(1)	2(1)
C(23)	18(1)	27(1)	29(2)	-3(1)	1(1)	2(1)
C(24)	13(1)	27(1)	22(1)	3(1)	2(1)	0(1)
C(25)	18(1)	22(1)	19(1)	2(1)	3(1)	-2(1)
C(26)	16(1)	22(1)	22(1)	1(1)	5(1)	4(1)
C(27)	27(2)	27(2)	27(2)	-6(1)	4(1)	0(1)
C(28)	16(1)	30(1)	24(1)	2(1)	3(1)	2(1)
C(29)	15(1)	27(2)	25(1)	3(1)	5(1)	-2(1)

C(30)	21(1)	27(2)	34(2)	-2(1)	0(1)	0(1)
C(31)	24(2)	37(2)	45(2)	1(1)	-9(1)	4(1)
C(32)	26(2)	44(2)	40(2)	-4(2)	-10(1)	-5(1)
C(33)	29(2)	35(2)	44(2)	-12(1)	3(2)	-9(1)
C(34)	19(1)	26(2)	40(2)	-1(1)	5(1)	0(1)
O(6)	19(1)	49(1)	35(1)	15(1)	-2(1)	-10(1)
O(7)	39(1)	33(1)	30(1)	-5(1)	-3(1)	17(1)
O(8)	38(1)	27(1)	33(1)	3(1)	5(1)	1(1)
O(9)	32(1)	30(1)	27(1)	7(1)	3(1)	6(1)
O(10)	22(1)	35(1)	30(1)	4(1)	1(1)	-2(1)
N(3)	13(1)	27(1)	19(1)	1(1)	0(1)	-2(1)
N(4)	18(1)	26(1)	19(1)	-2(1)	-2(1)	4(1)
C(35)	16(1)	22(1)	20(1)	0(1)	-1(1)	0(1)
C(36)	22(1)	22(1)	20(1)	-2(1)	2(1)	4(1)
C(37)	17(1)	23(1)	21(1)	0(1)	0(1)	1(1)
C(38)	15(1)	24(1)	22(1)	-1(1)	3(1)	-4(1)
C(39)	19(1)	23(1)	22(1)	-1(1)	1(1)	1(1)
C(40)	16(1)	24(1)	21(1)	2(1)	3(1)	-3(1)
C(41)	37(2)	36(2)	27(2)	-10(1)	-2(1)	12(1)
C(42)	34(2)	38(2)	38(2)	-6(1)	-2(1)	16(1)
C(43)	22(2)	37(2)	28(2)	3(1)	-1(1)	4(1)
C(44)	28(2)	38(2)	25(2)	-6(1)	-3(1)	3(1)
C(45)	24(2)	29(2)	27(2)	-5(1)	-1(1)	5(1)
C(46)	25(1)	24(1)	20(1)	-1(1)	3(1)	1(1)
C(47)	20(1)	28(1)	18(1)	-2(1)	4(1)	5(1)
C(48)	22(1)	25(1)	21(1)	1(1)	4(1)	0(1)
C(49)	25(2)	37(2)	22(1)	6(1)	6(1)	6(1)
C(50)	19(1)	44(2)	21(1)	-6(1)	2(1)	4(1)
C(51)	23(1)	32(2)	31(2)	-9(1)	6(1)	-3(1)
C(52)	27(2)	24(1)	24(1)	-1(1)	6(1)	3(1)
C(53)	20(1)	29(1)	21(1)	-5(1)	2(1)	0(1)
C(54)	21(1)	25(1)	19(1)	-5(1)	5(1)	6(1)
C(55)	25(1)	25(1)	27(2)	-5(1)	7(1)	1(1)
C(56)	30(2)	23(1)	26(2)	-1(1)	9(1)	3(1)
C(57)	38(2)	35(2)	31(2)	6(1)	7(1)	4(1)
C(58)	26(2)	29(2)	20(1)	0(1)	4(1)	9(1)
C(59)	19(1)	28(1)	24(1)	-2(1)	4(1)	2(1)
C(60)	21(1)	26(1)	23(1)	0(1)	6(1)	4(1)
C(61)	23(1)	34(2)	33(2)	5(1)	8(1)	0(1)
C(62)	19(1)	30(1)	27(1)	5(1)	7(1)	4(1)
C(63)	19(1)	26(2)	26(2)	3(1)	7(1)	-1(1)

C(64)	24(2)	28(2)	39(2)	-5(1)	3(1)	1(1)
C(65)	27(2)	38(2)	53(2)	3(2)	-5(2)	3(1)
C(66)	32(2)	50(2)	42(2)	-6(2)	-4(2)	-8(2)
C(67)	30(2)	39(2)	48(2)	-14(2)	7(2)	-10(1)
C(68)	24(2)	30(2)	44(2)	-1(1)	11(1)	-1(1)

Table S8. Hydrogen coordinates (x 10^4) and isotropic displacement parameters (E^2x 10^3) for Tib2023_04b_1_0m.

	x	y	z	U(eq)
H(1)	8391	5331	7432	23
H(2A)	8530	6811	6492	23
H(2B)	8765	8426	6919	23
H(3)	9237	4062	7601	23
H(4A)	9194	6413	8455	24
H(4B)	9057	8330	7956	24
H(7)	9032	1100	8719	37
H(8)	9398	30	9605	43
H(9)	9513	2254	10548	37
H(10)	9268	5548	10605	33
H(11)	8917	6718	9701	28
H(14)	8757	6040	5467	27
H(15)	8408	5668	4491	29
H(16)	8077	2750	4385	29
H(17)	8083	272	5262	32
H(18)	8433	616	6233	29
H(19A)	8316	8384	8157	27
H(19B)	8496	9881	7722	27
H(21)	8022	12090	7709	26
H(23A)	7154	14095	6274	30
H(23B)	7068	12432	6824	30
H(26)	8051	6465	6638	24
H(27A)	7886	5946	5443	41
H(27B)	7591	4612	5174	41
H(27C)	7764	4207	5918	41
H(28A)	9481	5959	6842	28
H(28B)	9331	8219	6999	28
H(30)	9863	4311	7723	34
H(31)	10256	4991	8585	44
H(32)	10284	8313	9162	46
H(33)	9929	11013	8847	44
H(34)	9546	10378	7960	34
H(35)	8349	159	2544	24
H(36A)	8470	1555	1588	26
H(36B)	8694	3312	1979	26

H(37)	9200	-939	2660	25
H(38A)	9158	1382	3525	24
H(38B)	9005	3266	3033	24
H(41)	9050	-3826	3725	41
H(42)	9421	-4944	4585	45
H(43)	9517	-2952	5579	36
H(44)	9242	166	5710	37
H(45)	8867	1272	4846	33
H(48)	8738	1740	629	27
H(49)	8427	1660	-412	33
H(50)	8157	-1487	-756	34
H(51)	8213	-4614	-86	34
H(52)	8535	-4582	937	30
H(53A)	8228	3034	3211	28
H(53B)	8461	4619	2954	28
H(55)	8307	6988	2055	30
H(57A)	7721	8156	111	41
H(57B)	7540	9879	482	41
H(60)	7794	1726	2311	28
H(61A)	7428	-281	1706	45
H(61B)	7085	285	1501	45
H(61C)	7273	1340	2162	45
H(62A)	9432	1083	1894	30
H(62B)	9283	3301	2091	30
H(64)	9811	-727	2760	37
H(65)	10206	-192	3628	49
H(66)	10257	3111	4217	51
H(67)	9912	5883	3926	46
H(68)	9523	5387	3044	38

Figure S26. The large-amplitude motions (LAM), proceeding in *R*,*R*-**1**.

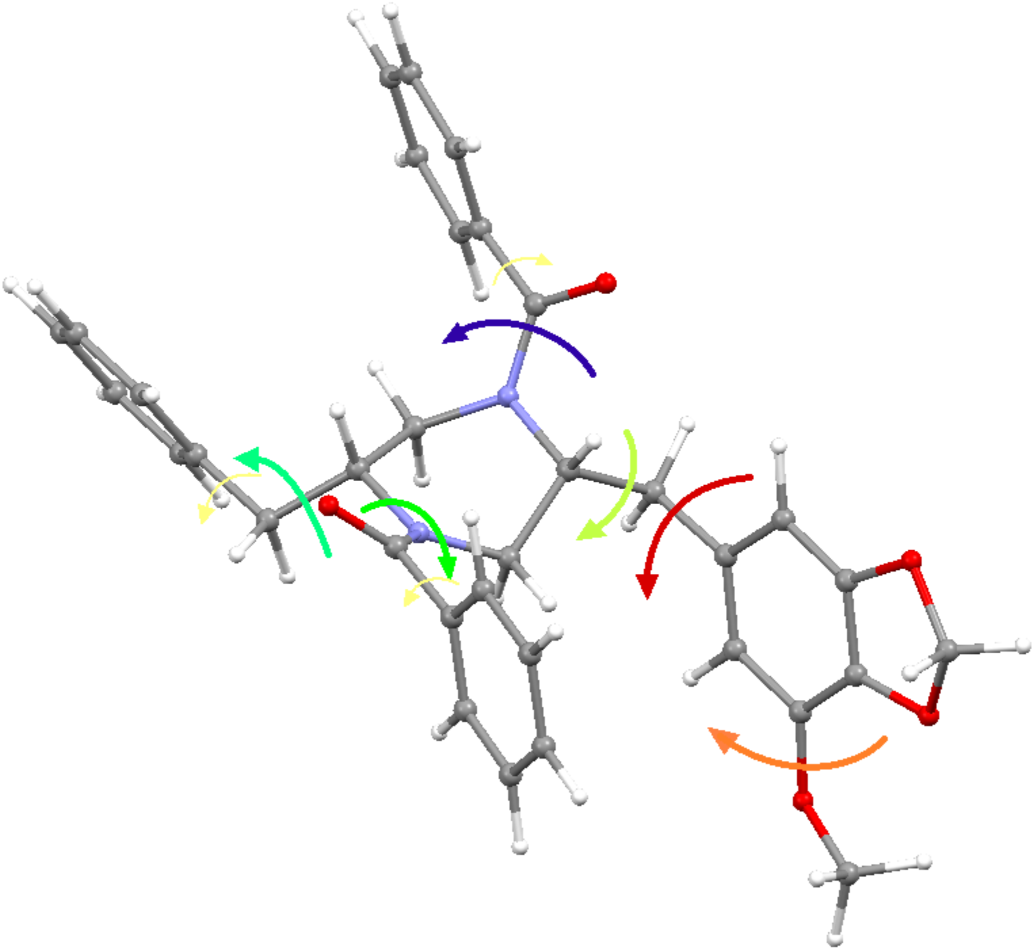

Figure S27. The most stable conformations of *R*,*R*-**1**. Corresponding statistical weights are presented in square brackets.

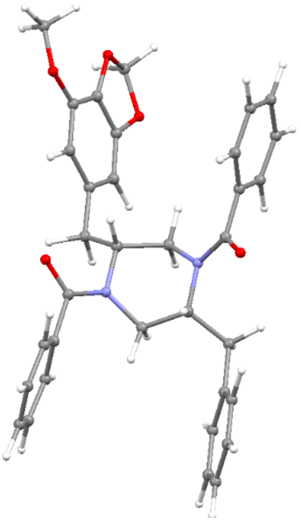

R,R - **1** - i1c ; [0.09]

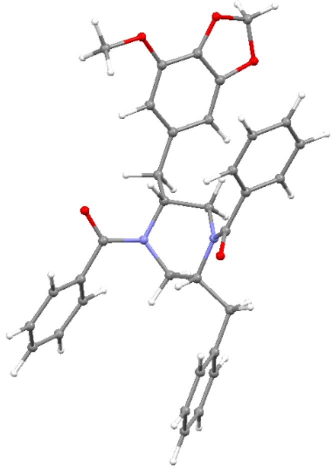

R,R - **1** - i1t ; [0.10]

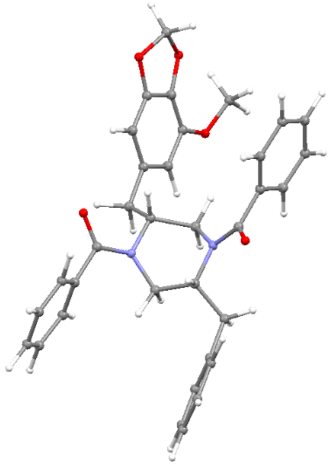

R,R - **1** - i1rc ; [0.14]

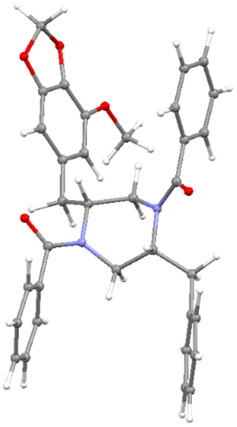

R,R - **1** - i1rt ; [0.13]

Figure S28. The conformational rearrangement, proceeding due to inversion of the piperazine ring.

The calculated total amount of the i2-type conformations is less then 0.1%.

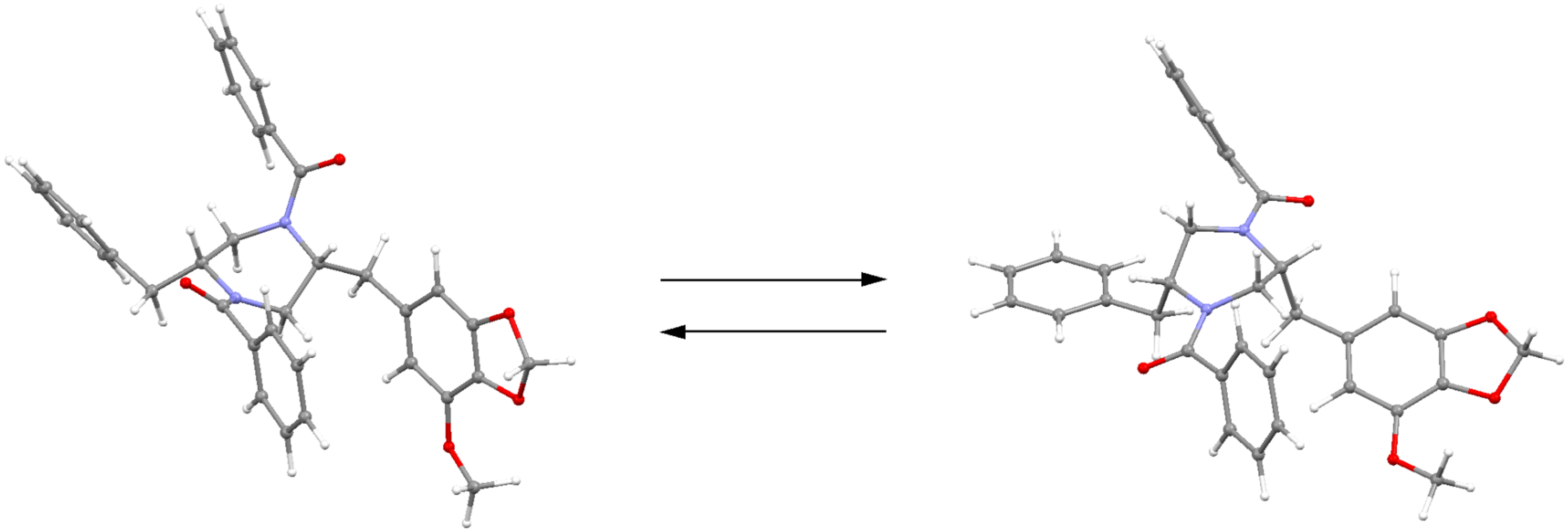

R,R - **1** - i1rc

R,R - **1** - i2rc

Figure S29. The most stable conformations of *R*,*R*-**2a** and *S*,*S*-**2a,** *R*,*R*-**2b** and *S*,*S*-**2b**.

Corresponding statistical weights are presented in square brackets.

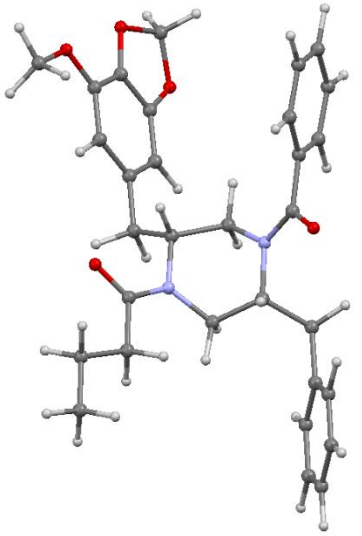

R,R -2_1_1 [0.29]

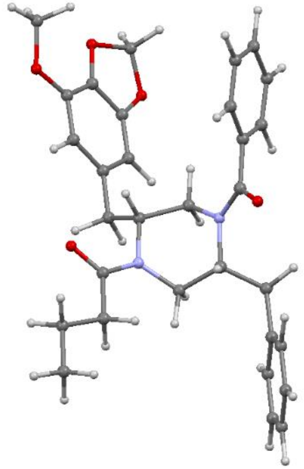

R,R -2_1_2 [0.20]

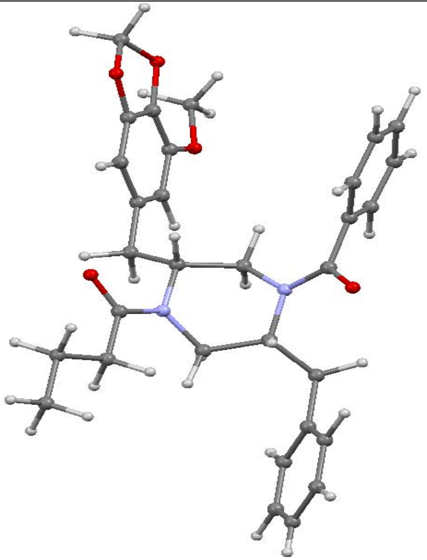

R,R-2_1_3 [0.23]

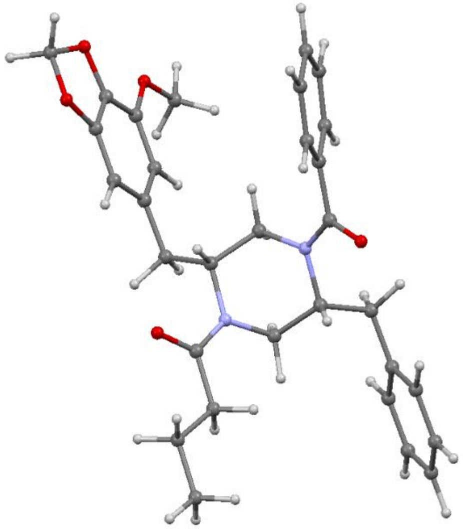

R,R -2_1_4 [0.28]

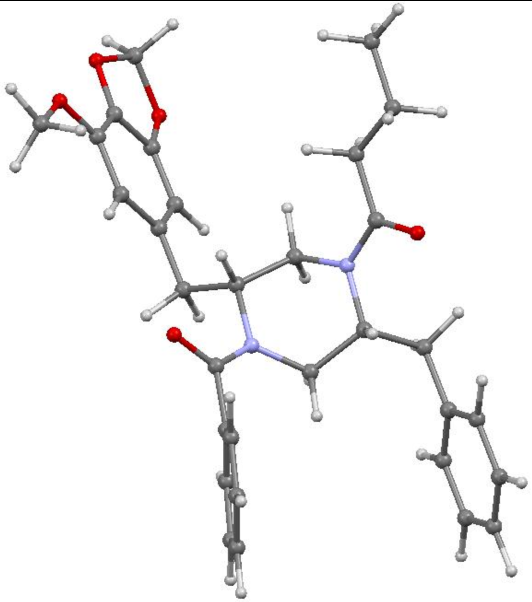<br>*S,S*-2_2_1 [0.44]	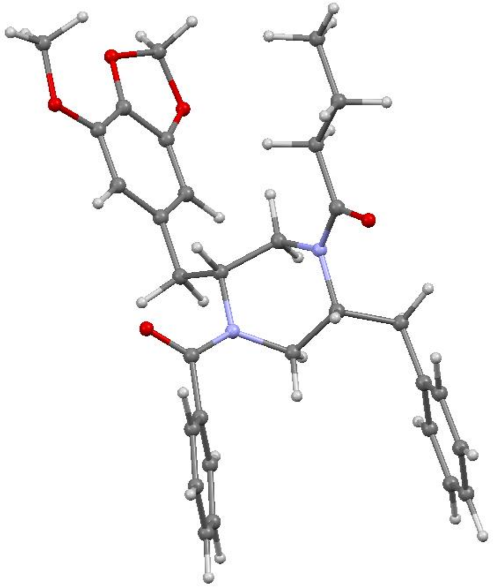<br>*S,S*-2_2_2 [0.22]
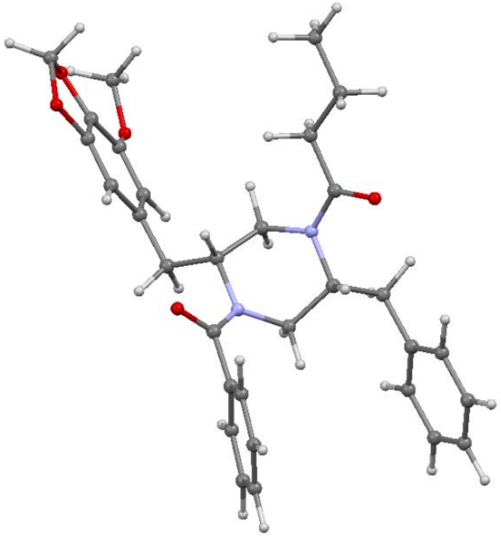<br>*S,S*-2_2_3 [0.27]	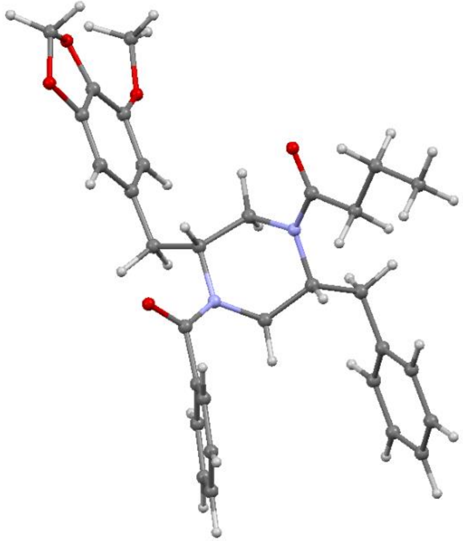<br>*S,S*-2_2_4 [0.07]

Figure S30. LC-UV chromatograms of extracts from fungus *Penicillium velutinum* ZK-14 cultivated with metal ions. Chromatograms were recorded at 290 nm.

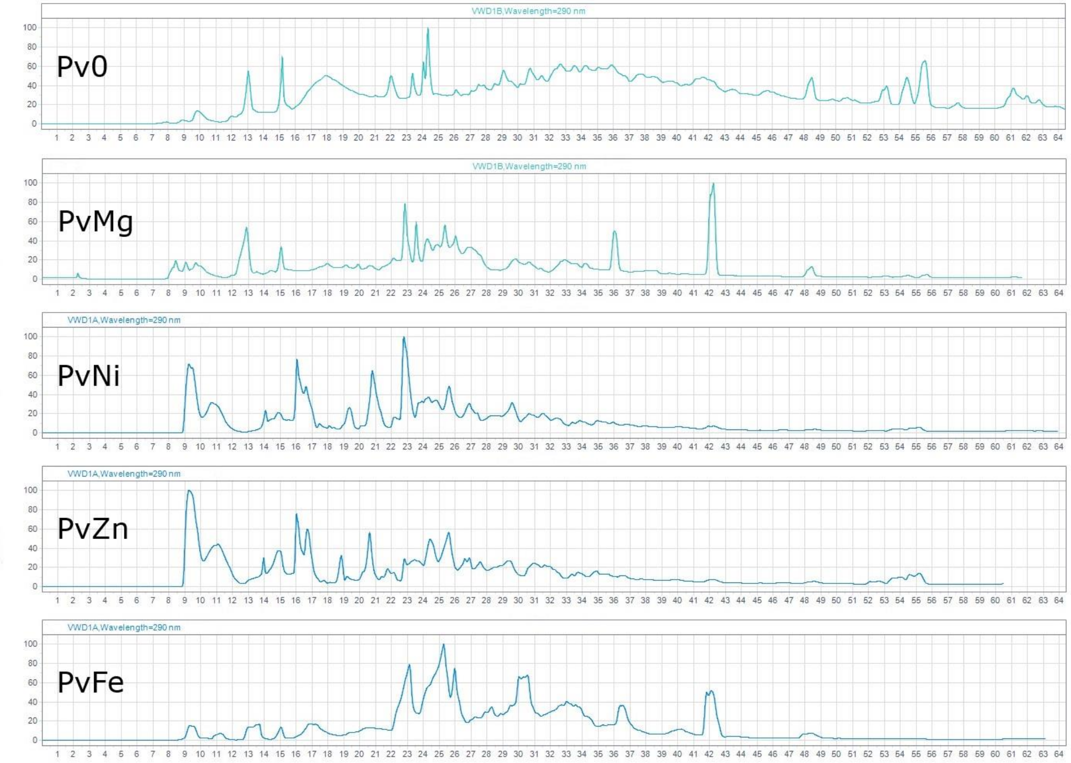

Figure S31. HPLC UV chromatogram of the extract of the fungus *Penicillium velutinum* (ZK-14) cultivated on rise medium (**Pv0**). Chromatograms were recorded at 220 nm (blue) and 290 nm (green).

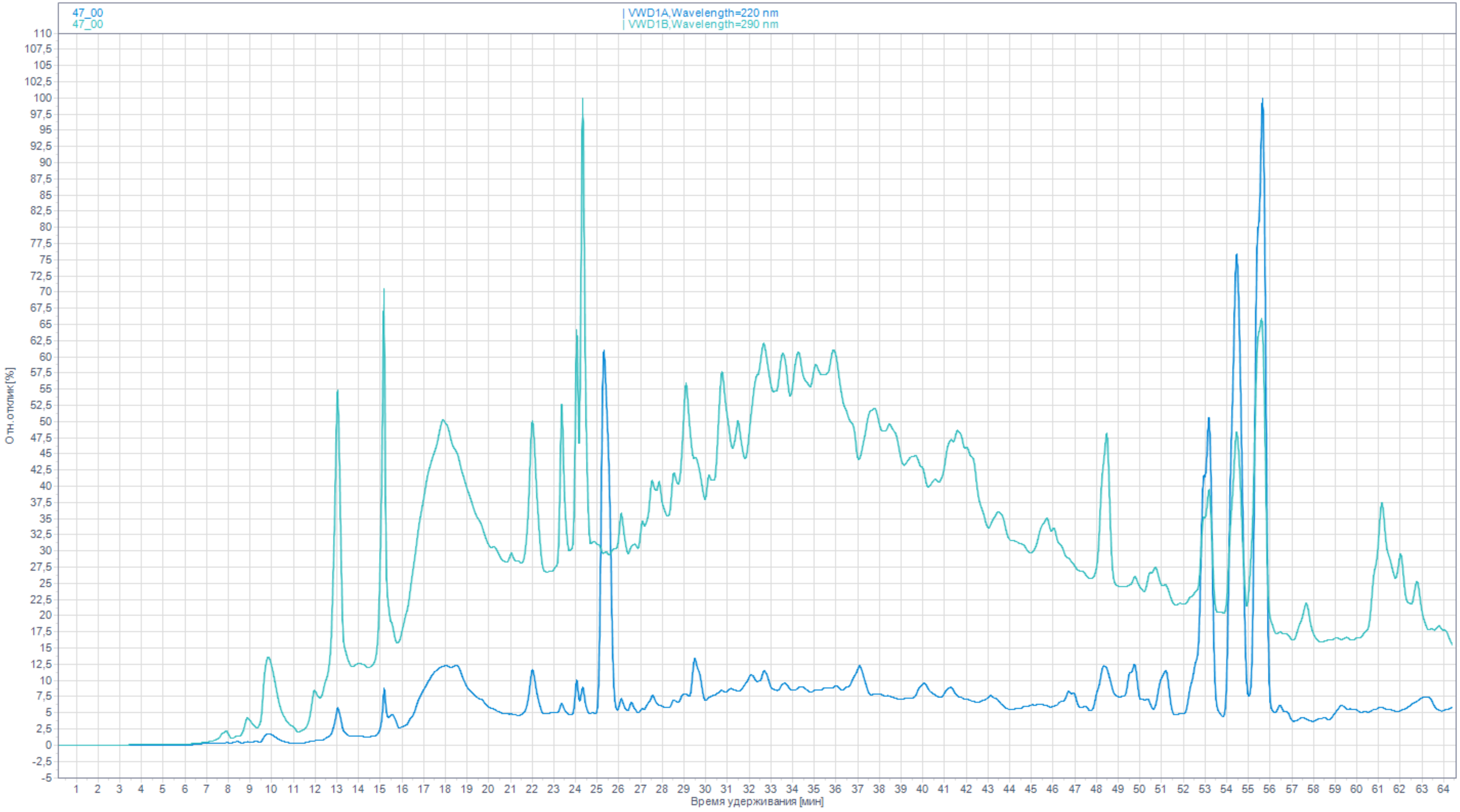

Figure S32. HPLC UV chromatogram of the extract of the fungus *Penicillium velutinum* (ZK-14) cultivated on rise medium with 100 µm Mg^{2+} salt concentration (**PvMg**).
Chromatograms were recorded at 220 nm (blue) and 290 nm (green).

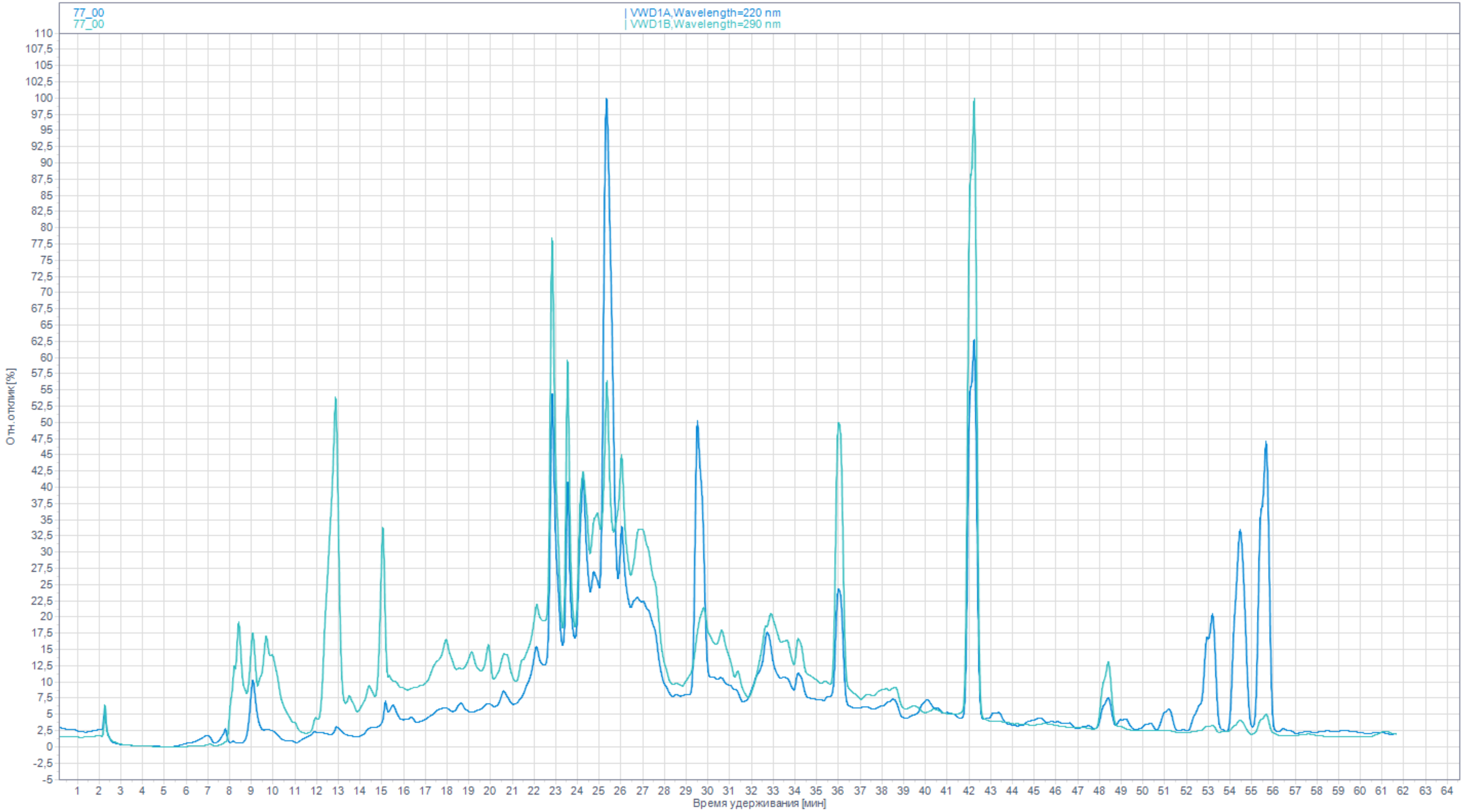

Figure S33. HPLC UV chromatogram of the extract of the fungus *Penicillium velutinum* (ZK-14) cultivated on rise medium with 100 μm Fe^{3+} salt concentration (**PvFe**). Chromatograms were recorded at 220 nm (blue) and 290 nm (green).

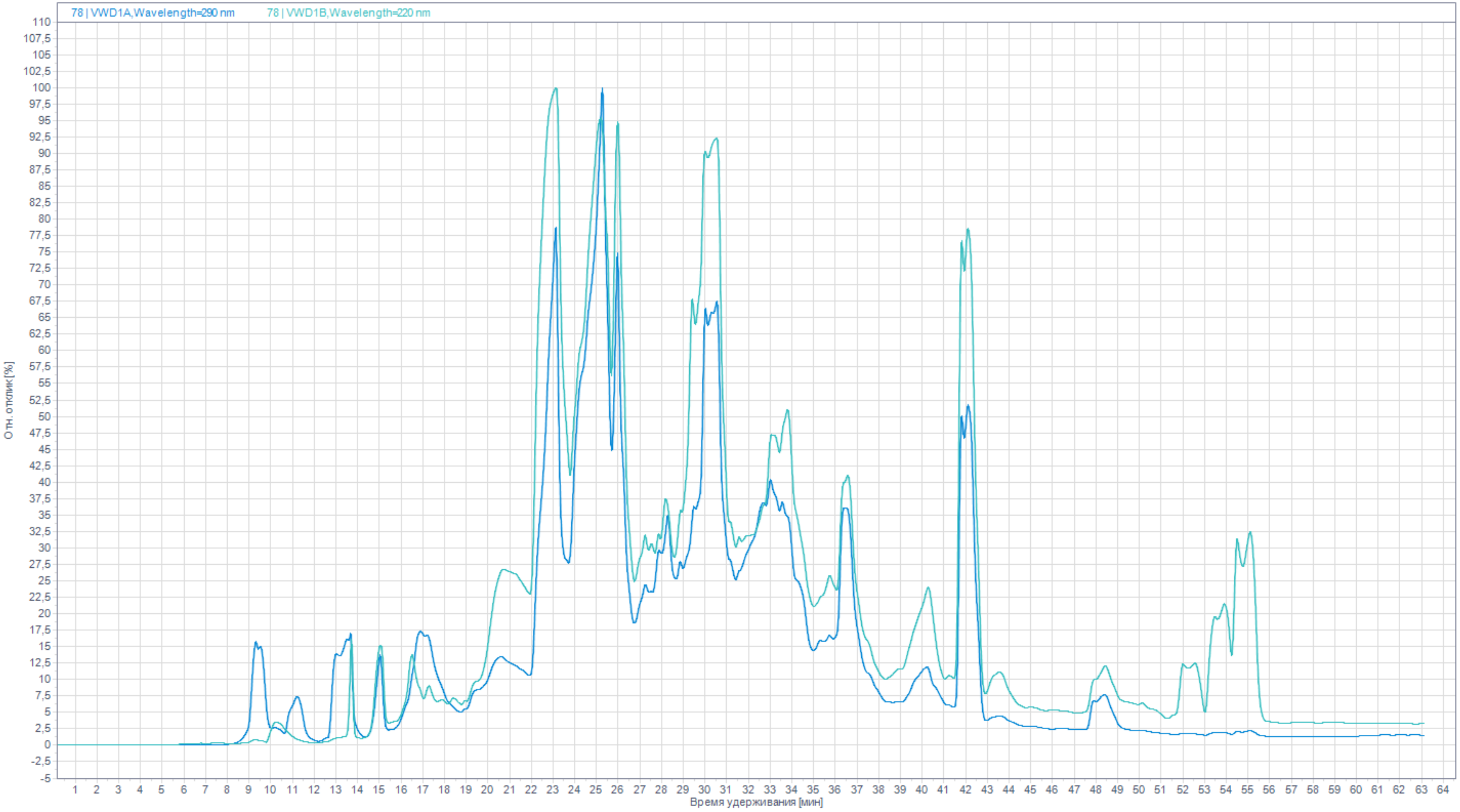

Figure S34. HPLC UV chromatogram of the extract of the fungus *Penicillium velutinum* (ZK-14) cultivated on rise medium with 100 µm Zn^{2+} salt concentration (**PvZn**). Chromatograms were recorded at 220 nm (blue) and 290 nm (green).

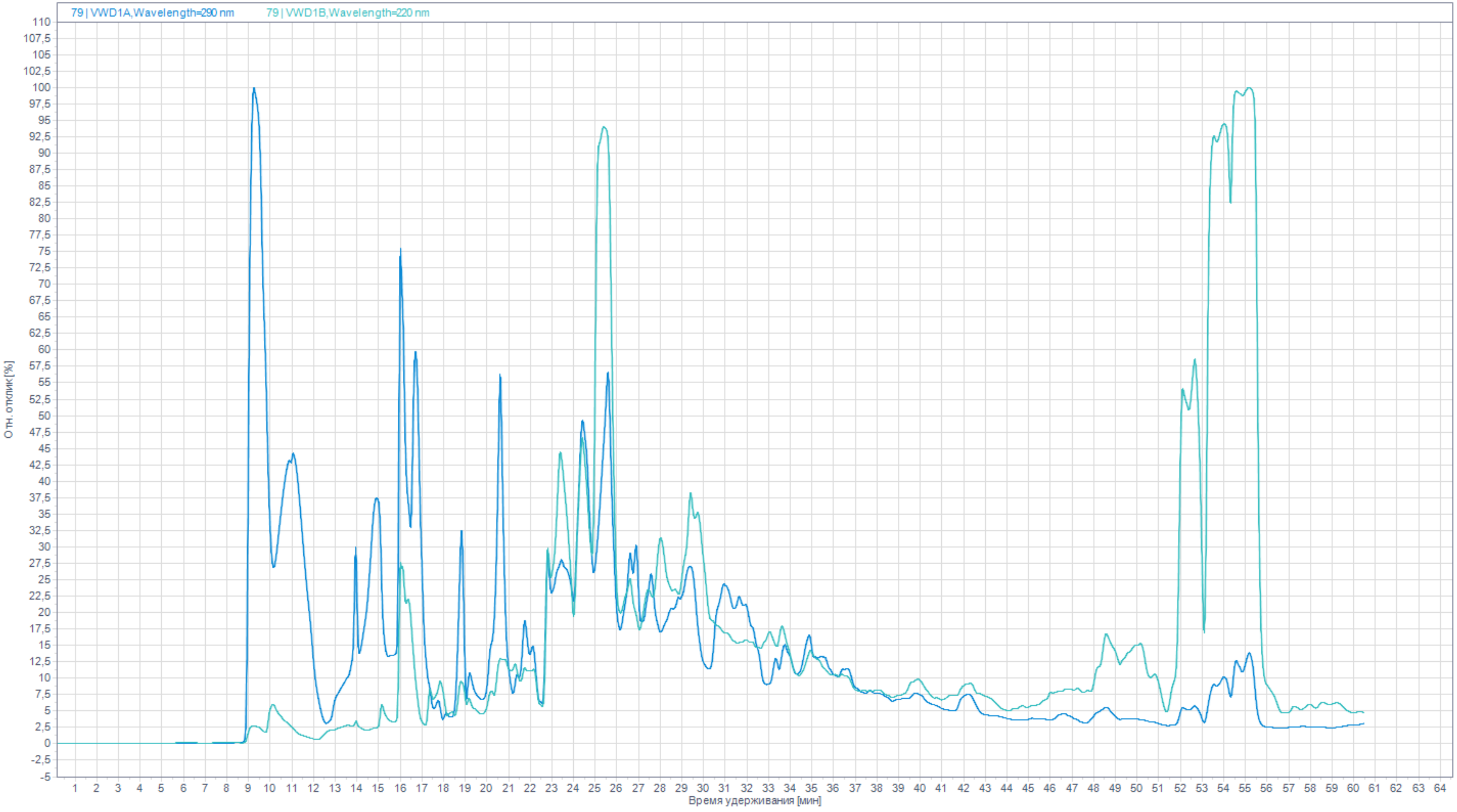

Figure S35. HPLC UV chromatogram of the extract of the fungus *Penicillium velutinum* (ZK-14) cultivated on rise medium with 100 µm Ni^{2+} salt concentration (**PvNi**). Chromatograms were recorded at 220 nm (blue) and 290 nm (green).

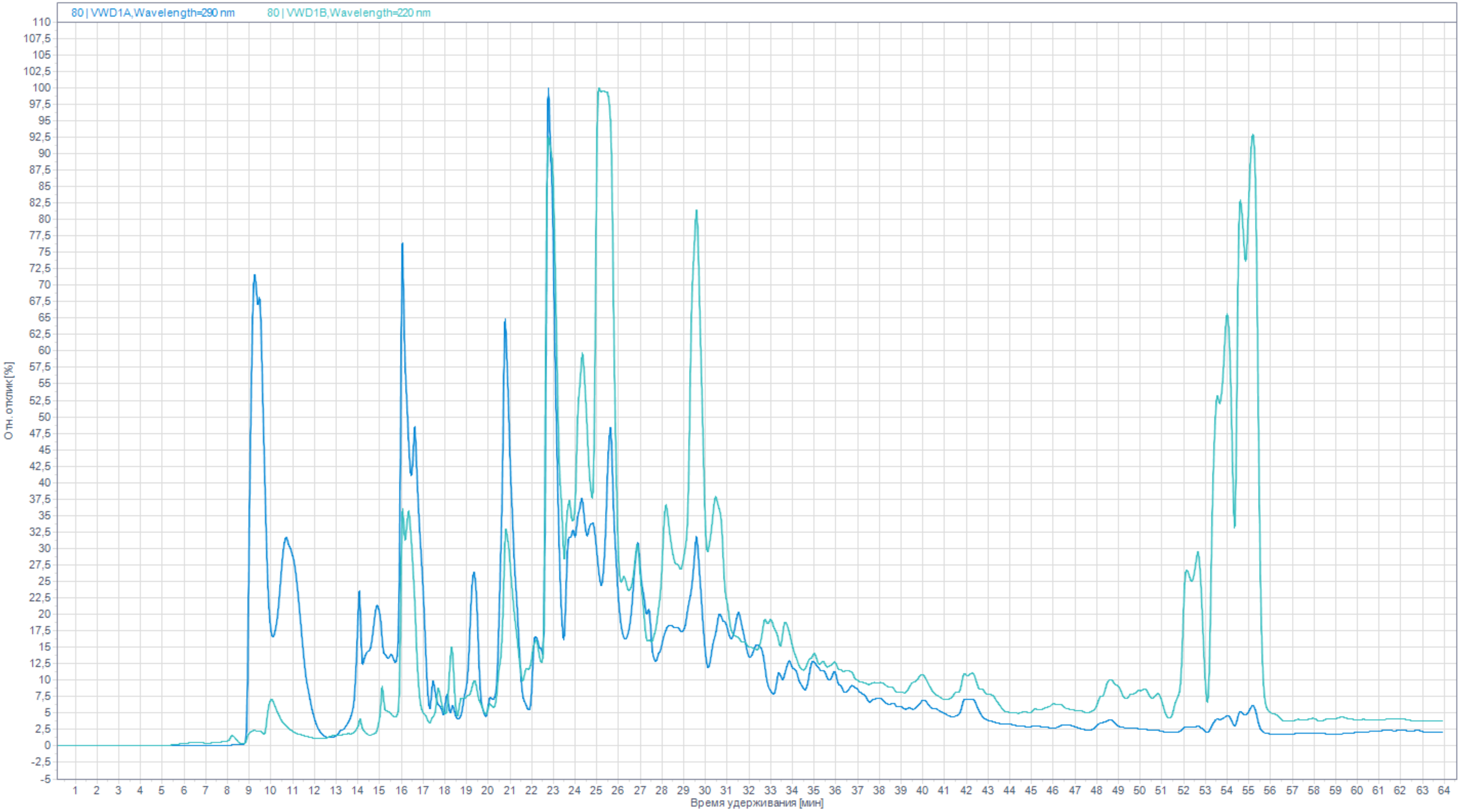

Figure S36. HPLC MS retention time and MS/MS of compound **1**.

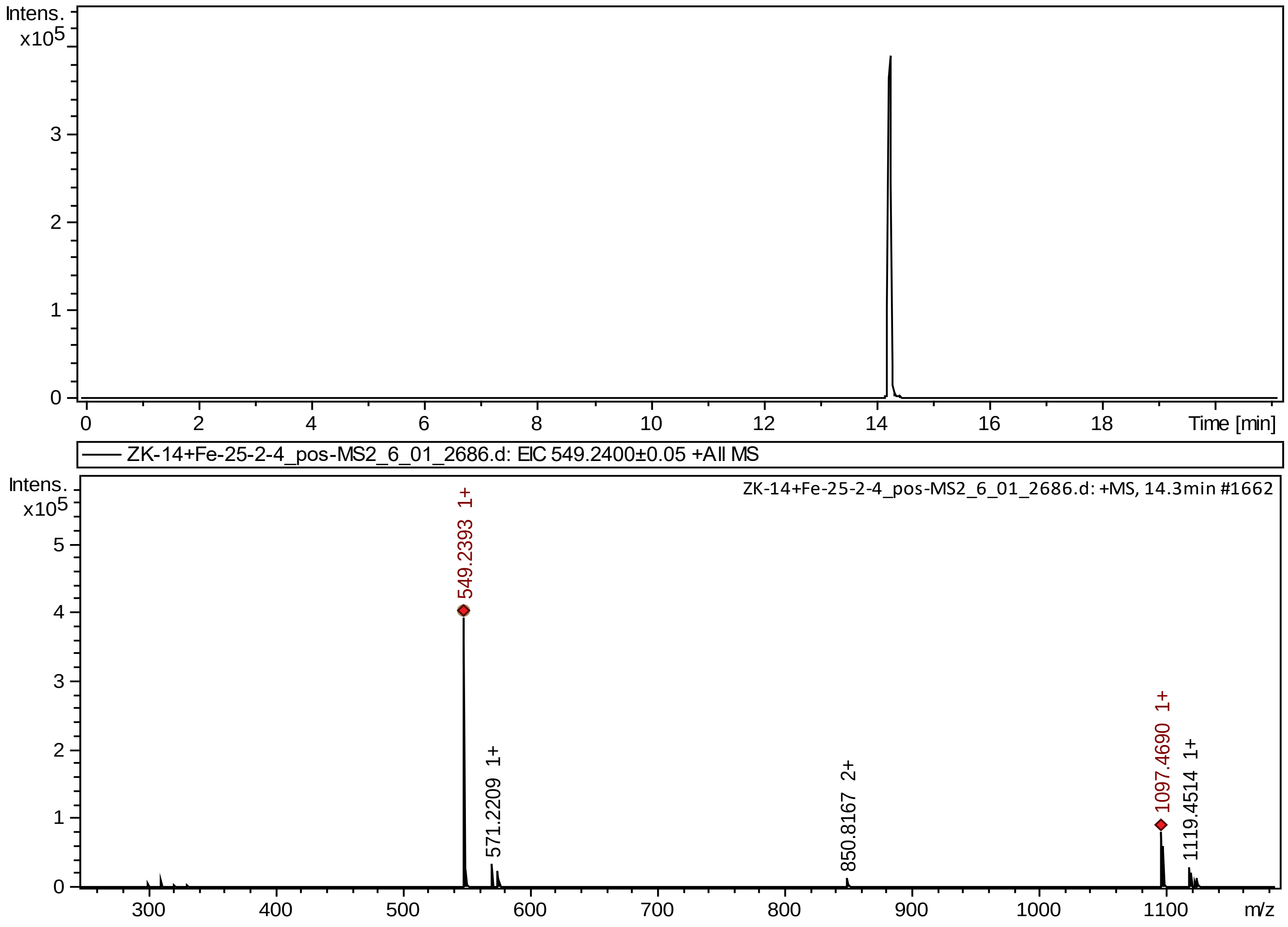

Figure S37. HPLC MS retention time and MS/MS of compound **2**.

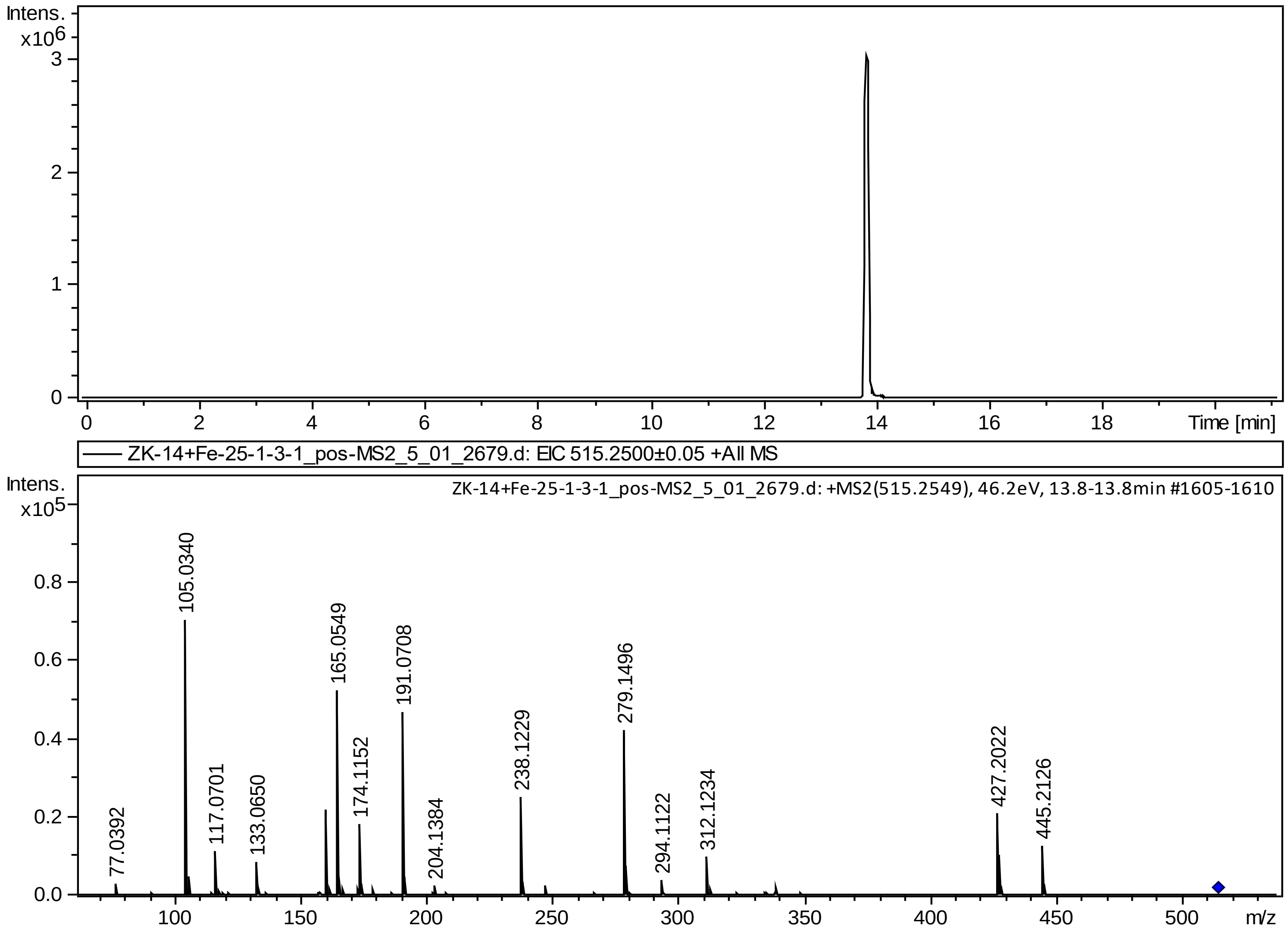

Figure S38. HPLC MS retention time and MS/MS of (4S,5R,7S)-4,11-dihydroxy-guaia-1(2),9(10)-dien **4**.

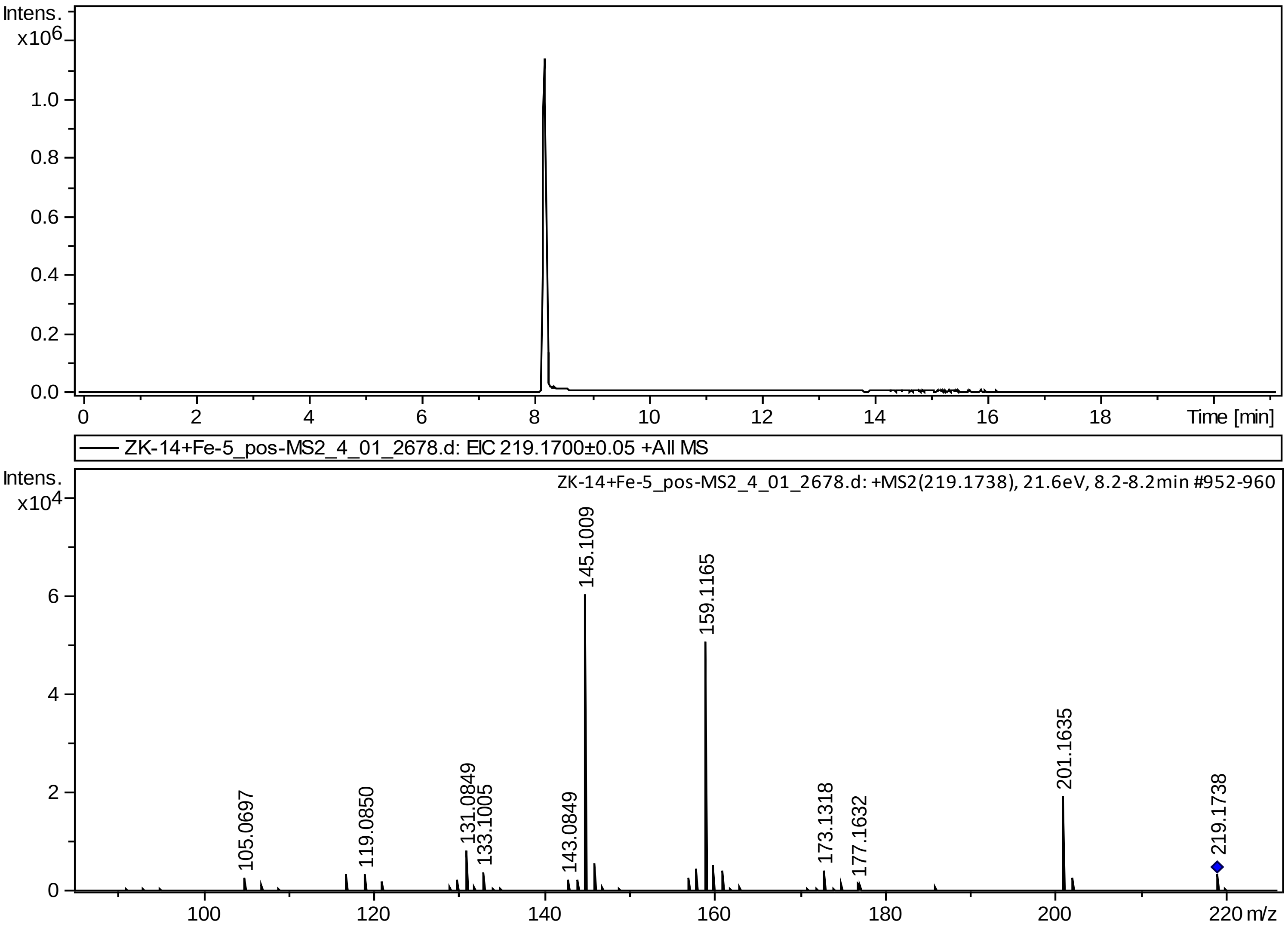

Figure S39. HPLC MS retention time and MS/MS of peak **VIII.**

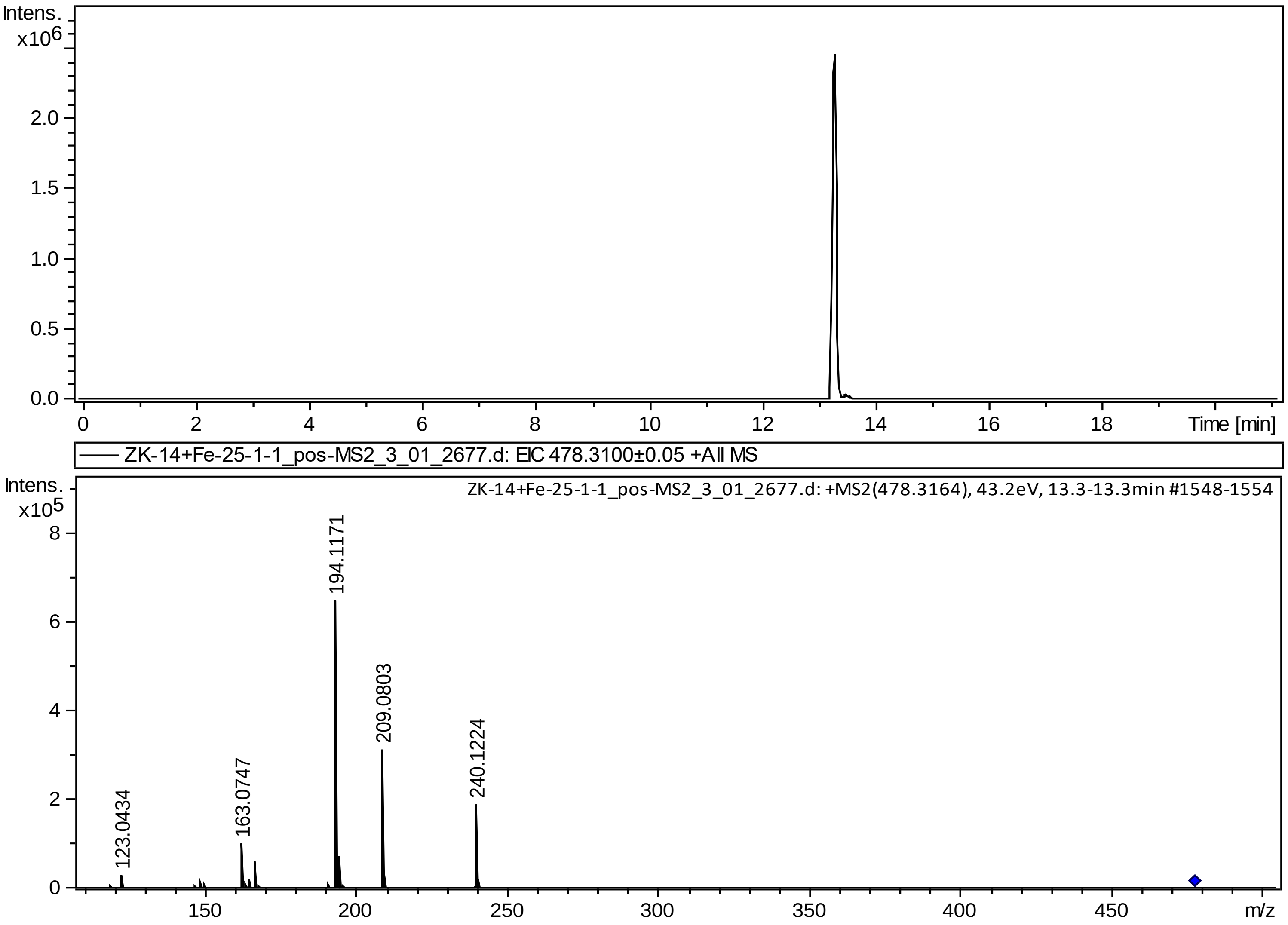

Figure S40. HPLC MS retention time and MS/MS of peak **IXa**.

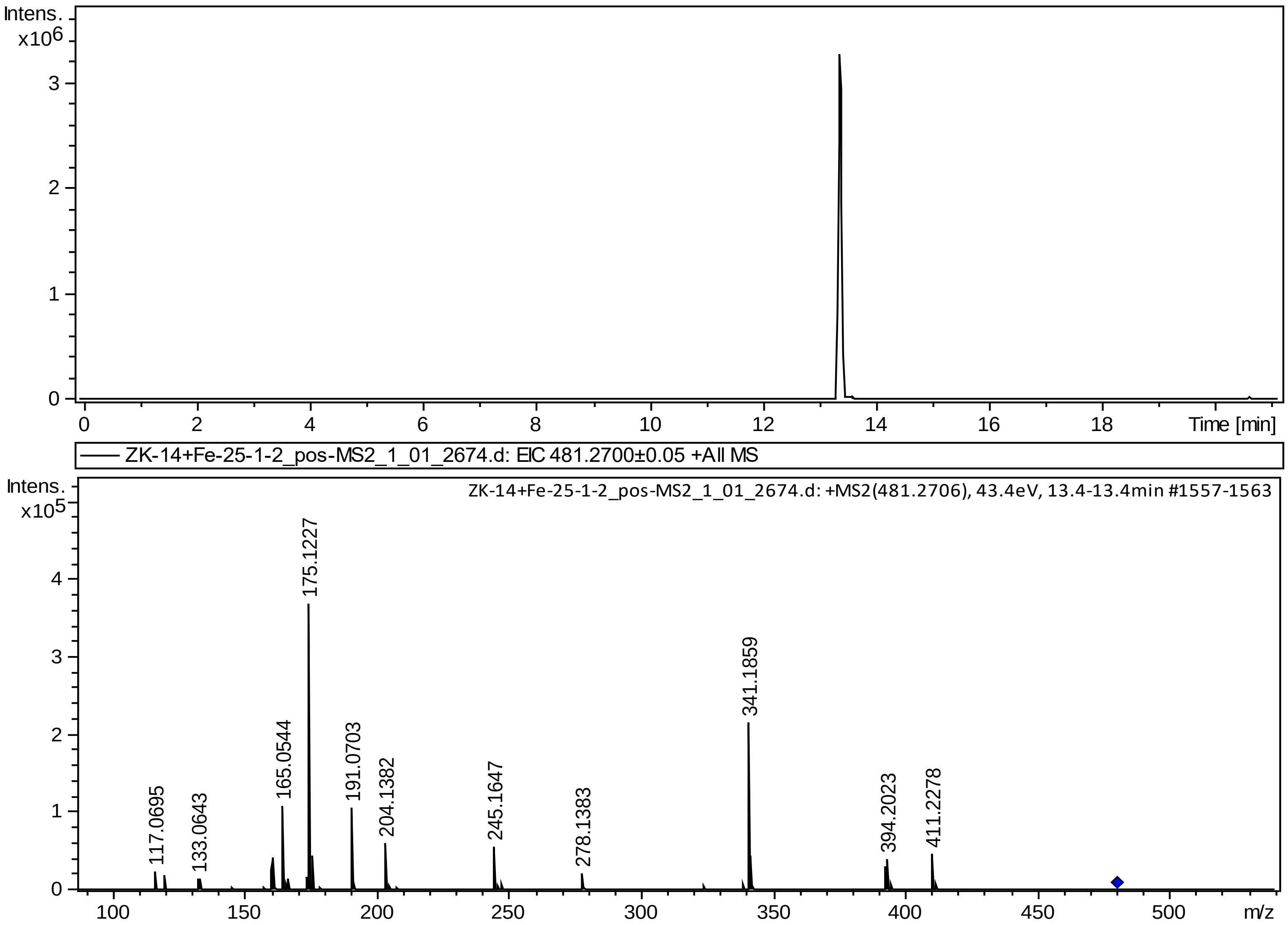

Figure S41. HPLC MS retention time and MS/MS of peak **X**.

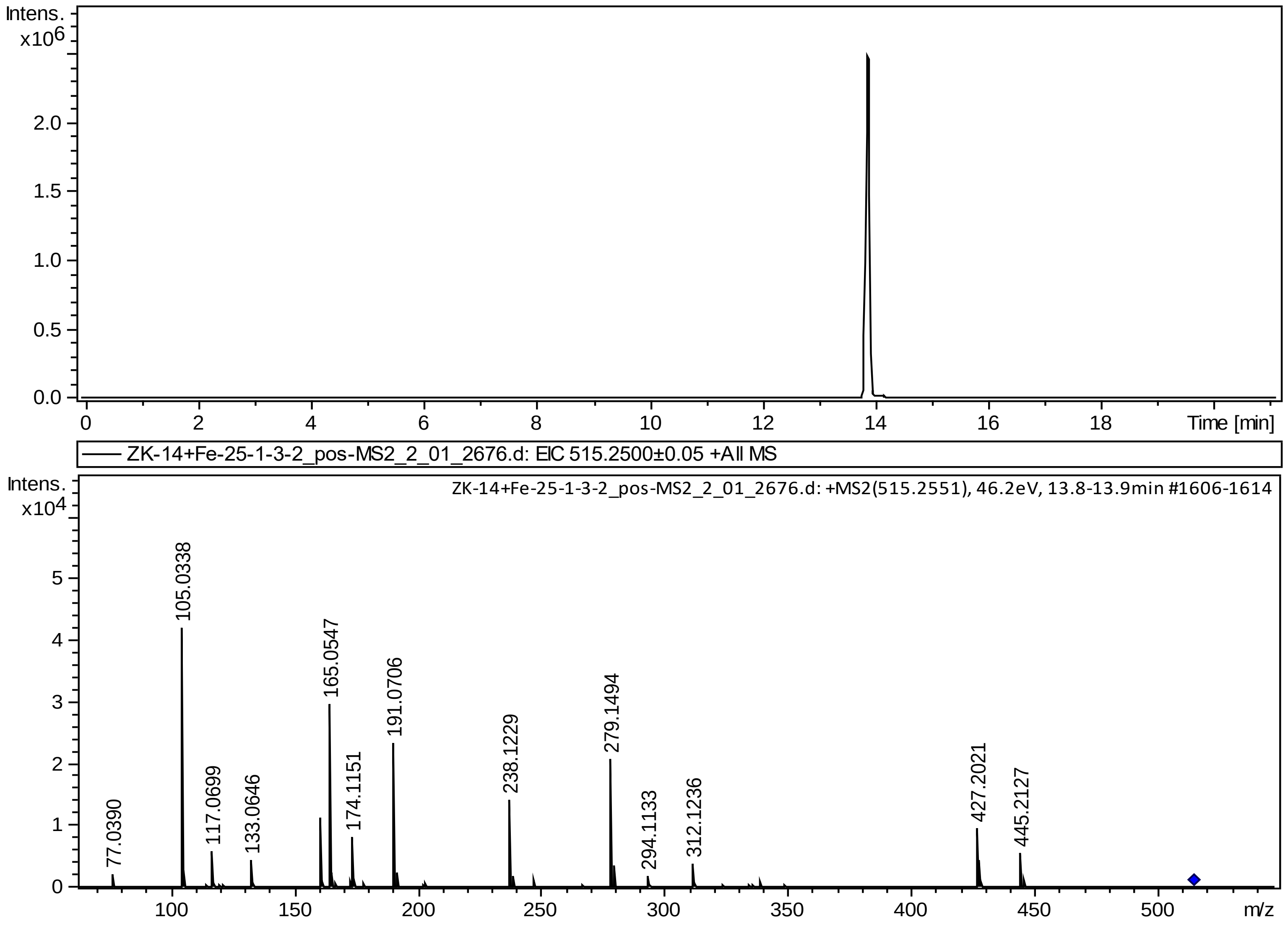

Figure S42. The MZmine processing settings

Mass detection was carried out at the MS1 level and MS2 level with noise level thresholds of 60 and 40, respectively. Chromatograms were made with the ADAP Chromatogram Builder Module [1] with the following parameters: Min group size in # of scans was set to 6, group intensity threshold and Min highest intensity were set to 130 and 300, respectively, *m/z* tolerance was set to 0.05 *m/z*. The chromatogram deconvolution module was used with the ADAP algorithm with signal/noise threshold of 8, min feature height of 300, coefficient/area threshold of 40, peak duration range was set from 0 to 2.0, and RT wavelet range was set from 0 to 0.1. The *m/z* center calculation was set to MEDIAN. The Isotopics peaks grouper module was used with an *m/z* tolerance of 5 ppm, retention time tolerance of 0.1 min, the monotonic shape function set to true, a maximum charge of 2 and the representative isotope set to the most intense. Alignment was achieved with the Join aligner function with an *m/z* tolerance of 5 ppm, a weight for *m/z* at 50, a retention time tolerance of 0.1 min, and a weight for RT at 50. The Require same charge state, Require same ID, and the Compare spectra similarity functions were set to false. The aligned feature list was exported by the Export/Submit to "GNPS-FBMN" module with the Merge MS/MS (experimental) function with the following parameters: Select spectra to merge was set to across samples, the *m/z* merge mode was set to weighted average (remove outliers), the intensity merge mode was set to sum intensities, the expected mass deviation was set to 5 ppm, the cosine threshold was set to 70%, the peak count threshold was set to 20%, the isolation window offset (*m/z*) was set to 0, and the isolation window width (*m/z*) was set to 3.

1. Myers, O.D.; Sumner, S.J.; Li, S.; Barnes, S.; Du, X. One Step Forward for Reducing False Positive and False Negative Compound Identifications from Mass Spectrometry Metabolomics Data: New Algorithms for Constructing Extracted Ion Chromatograms and Detecting Chromatographic Peaks. *Analytical Chemistry* **2017**, *89*, 8696-8703.
